# Gain-Sparsity and Symmetry-Forced Rigidity in the Plane

**DOI:** 10.1007/s00454-015-9755-1

**Published:** 2016-02-01

**Authors:** Tibor Jordán, Viktória E. Kaszanitzky, Shin-ichi Tanigawa

**Affiliations:** Department of Operations Research, Eötvös University, and the MTA-ELTE Egerváry Research Group on Combinatorial Optimization, Pázmány Péter sétány 1/C, 1117 Budapest, Hungary; Department of Operations Research, Eötvös University, Pázmány Péter sétány 1/C, 1117 Budapest, Hungary; Department of Mathematics and Statistics, Lancaster University, Lancaster, LA1 4YF UK; Research Institute for Mathematical Sciences, Kyoto University, Sakyo-ku, Kyoto 606-8502 Japan; Centrum Wiskunde & Informatica (CWI), Postbus 94079, 1090 GB Amsterdam, The Netherlands

**Keywords:** Infinitesimal rigidity, Frameworks, Symmetry, Rigidity of graphs, Rigidity matroids, Group-labeled graphs, Frame matroids, Primary 52C25, Secondary 05B35, 68R10

## Abstract

We consider planar bar-and-joint frameworks with discrete point group symmetry in which the joint positions are as generic as possible subject to the symmetry constraint. We provide combinatorial characterizations for symmetry-forced rigidity of such structures with rotation symmetry or dihedral symmetry of order 2*k* with odd *k*, unifying and extending previous work on this subject. We also explore the matroidal background of our results and show that the matroids induced by the row independence of the orbit matrices of the symmetric frameworks are isomorphic to gain sparsity matroids defined on the quotient graph of the framework, whose edges are labeled by elements of the corresponding symmetry group. The proofs are based on new Henneberg type inductive constructions of the gain graphs that correspond to the bases of the matroids in question, which can also be seen as symmetry preserving graph operations in the original graph.

## Introduction

A *d*-dimensional *bar-and-joint framework* (or, simply, a *framework*) is a straight-line realization of a finite simple graph *G* in Euclidean *d*-space. We think of a bar-and-joint framework as a collection of fixed-length bars (corresponding to the edges of *G*) which are connected at their ends by universal joints (corresponding to the vertices of *G*). Frameworks can be used to model various structures with pairwise distance constraints and whose rigidity property is of particular interest in applications ranging from civil engineering [9, 25] and crystallography [27] to sensor network localization [7] and biochemistry [29]. In several applications the model frameworks may have symmetry, which makes it important to explore the impact of symmetry on the flexibility and rigidity of the framework.

In the past 10 years this research area has received an ever increasing attention which has led to rigorous definitions, a clear separation of different directions and a number of new results (see, e.g., [3, 16, 19]). One of the general goals of the research is to extend Laman’s classical theorem on generically rigid planar frameworks (with no symmetry conditions). The works initiated by Ross [17] and Malestein and Theran [11] gave natural extensions of Laman’s theorem to periodic frameworks in the plane, where the ingenious idea is to look at count conditions for quotient graphs with group labelings.

This paper deals with finite bar-and-joint frameworks with point group symmetry in the *symmetry-forced* setting and extends Laman’s classical theorem as well as its matroidal background and algorithmic implications, to planar frameworks with rotational or dihedral symmetry, assuming that the joint positions are as generic as possible subject to the symmetry conditions. In our symmetry-forced setting, a framework is said to be *symmetry-forced flexible* if it has a non-trivial *symmetric* infinitesimal motion. For the generic frameworks that we consider, this is equivalent to the existence of a non-trivial symmetry preserving flex [21], and our main result characterizes symmetric frameworks that admit nontrivial symmetry preserving flexes in terms of simple count conditions of the underlying quotient group-labeled graphs, which can be checked in polynomial time by combinatorial algorithms.

By using the orbit rigidity matrix introduced by Schulze and Whiteley [23], we can reformulate our problem in terms of the generic rank of a matrix in which each row corresponds to an edge orbit and each vertex orbit has two columns. This in turn is equivalent to characterizing independence in a matroid defined on the edge set of the group-labeled quotient graph, in which vertices and edges correspond to vertex and edge orbits, respectively, and which concisely represents the graph structure with the corresponding symmetry. Our main results characterize these matroids in the case of rotation symmetry or dihedral symmetry ${{{\mathcal {D}}}}_{2k}$ of order 2*k* with odd *k*. If the underlying symmetry is cyclic, the matroid turns out to be a (*k*, *l*)-*gain-count matroid*, in which independence is defined by imposing certain sparsity conditions on the edge set of a graph, whose edges are labeled by group elements. In the dihedral case the matroid arises by a related, but more general construction.

Matroids of the former type can be obtained by matroidal operations (e.g. matroid union and Dilworth truncation) from matroids that have been extensively studied in matroid theory and are called frame matroids (or bias matroids) [31, 32]. These matroids, and their relatives, which also play a role in the theory of infinite periodic frameworks [11, 12, 18], have been generalized in a recent paper [24] which unified most of the existing results on symmetric and periodic frameworks, including our cyclic case. However, the matroid of the dihedral case does not fit this general class.

We prove our results by developing Henneberg type inductive constructions for the bases of our matroids and show that these operations preserve the row-independence of the orbit rigidity matrix. This approach, which has been used in many combinatorial characterizations of rigidity theory, leads to the desired result. In our problems, due to the more complex sparsity conditions and the group labeling, we also need some new operations and extended geometric arguments, to handle the symmetry constraints.

The complete answer in the case of dihedral symmetry remains open. However, most of our inductive steps (extending or reducing a symmetric framework or a labeled graph, respectively) are valid also for dihedral groups ${{{\mathcal {D}}}}_{2k}$ with even *k*, and can be used to show that in the even case the irreducible graphs (frameworks), where our reduction operations are not applicable, are very special. Interestingly, the smallest such framework, which is predicted to be rigid by the matroidal count but is flexible is the Bottema mechanism, a well-known mechanism in the kinematics literature (see, e.g., [30]).

For the case when the underlying symmetry is cyclic, the same combinatorial characterizations were also given by Malestein and Theran [12, 13] by a completely different proof approach. The main contributions of this paper are (i) to develop a concise approach to analyze the rigidity of symmetric frameworks based on inductive constructions and (ii) to give the first combinatorial characterization for frameworks with non-cyclic symmetry, which is far more complicated than the cyclic case. After the publication of the technical report [8] of this paper, our formulation and results on inductive constructions were used for analyzing the infinitesimal rigidity of symmetric frameworks [15, 22] and the symmetric-forced rigidity of symmetric frameworks on surfaces [14]. Also the matroid construction given in Sect. 7 was recently generalized in [6].

The structure of the paper is as follows. In the rest of this section we introduce some basic notation. In Sect. 2 we define and investigate gain graphs, which are directed multigraphs with edges labeled by elements of a group. Gain count matroids, defined on gain graphs by sparsity conditions, are introduced in Sect. 3 along with the necessary matroidal background. In Sect. 4 we develop our inductive construction for the bases of a specific gain count matroid by using three operations and a single base graph. In Sect. 5 we recall the basic definitions and results needed to study symmetric frameworks, including the orbit rigidity matrix and the necessary count conditions. In Sect. 6 we prove the first geometric lemmas and use them, together with results of Sect. 4, to complete the characterization of rigid frameworks with cyclic symmetry. In Sect. 7 we prove the inductive construction for the bases of our second matroid by using five operations and four types of base graphs. In this case we need to handle graphs of minimum degree four and hence we need more operations and longer arguments. To make the paper more readable, the lengthy case, when the graph is four-regular, is moved to the end of the paper, to Sect. 9. In Sect. 8 we prove additional geometric lemmas and use them, together with the inductive construction of Sect. 7, to prove the second main result, the characterization of rigid frameworks with dihedral symmetry with odd *k*. We also present frameworks that meet the sparsity requirements but are dependent and flexible when *k* is even. In Sect. 10 we briefly discuss the algorithmic implications and make some further remarks.

In the rest of the introduction, let us introduce notation used throughout the paper.

Let *E* be a finite set. A *partition*${{{\mathcal {P}}}}$ of *E* is a family of nonempty subsets of *E* such that each element of *E* belongs to exactly one member of ${{{\mathcal {P}}}}$. If $E=\emptyset$, the partition of *E* is defined as the empty set. A *subpartition* of *E* is a partition of a subset of *E*.

Let $G=(V,E)$ be an undirected graph. For $v\in V$, let $d_G(v)$ be the degree of *v* in *G* and $N_G(v)$ be the set of neighbors of *v* in *G*. For $F\subseteq E$, $V_G(F)$ denotes the set of endvertices of edges in *F*, and let $G[F]=(V(F), F)$, that is, the graph edge-induced by *F*. If the graph is clear from the context, the subscript *G* may be dropped. For $F\subseteq E$ and $v\in V(F)$, let $d_F(v)=d_{G[F]}(v)$.

A vertex subset $X\subset V(G)$ (resp., an edge subset $X\subset E(G)$) is called a *separator* (resp., a *cut*) if the removal of *X* disconnects *G*. A separator *X* with $|X|=1$ is called a *cut-vertex*. *G* is called *k*-*connected* (resp., *k*-*edge-connected*) if the size of any separator (resp., any cut) is at least *k*. A separator (resp., a cut) is called *nontrivial* if its removal disconnects *G* into at least two nontrivial connected components, where a connected component is called trivial if it consists of a single vertex. *G* is called *essentiallyk*-*connected* (resp., *essentiallyk*-*edge-connected*) if the size of any nontrivial separator (resp., any nontrivial cut) is at least *k*.

For simplicity, some properties of edge-induced subgraphs will be associated with the corresponding edge sets as follows. Let $F\subseteq E$. *F* is called *connected* if *G*[*F*] is connected. A *connected component* of *F* is the edge set of a connected component of *G*[*F*]. *C*(*F*) denotes the partition of *F* into connected components of *F*, and let $c(F)=|C(F)|$. *F* is called a *forest* if it contains no cycle and called a *tree* if it is a connected forest. *F* is called a *spanning tree* of a graph $G=(V,E)$ if *F* is a tree with $F\subseteq E$ and $V(F)=V$.

Let $$G=(V,E)$$ be a directed graph. A *walk* in *G* is a sequence $$W=v_0,e_1,v_1,e_2,v_2,\ldots ,$$$$v_{k-1}, e_k,v_k$$ of vertices and edges such that $$v_{i-1}$$ and $$v_{i}$$ are the endvertices of $$e_i$$ for every $$1\le i\le k$$. We often denote a walk as a sequence of edges implicitly assuming the incidence at each vertex. For two walks *W* and $$W'$$ for which the end vertex of *W* and the starting vertex of $$W'$$ coincide, we denote the concatenation of *W* and $$W'$$ (that is, the walk *W* followed by $$W'$$) by $$W*W'$$. A walk is called *closed* if the starting vertex and the end vertex coincide.

It is sometimes convenient to regard the empty set as a subgroup of a group. Let ${{{\mathcal {D}}}}$ be a dihedral group. For a cyclic subgroup ${{{\mathcal {C}}}}$ of ${{{\mathcal {D}}}}$, $\bar{{{\mathcal {C}}}}$ denotes the maximal cyclic subgroup containing ${{{\mathcal {C}}}}$.

## Gain Graphs

In this section we shall review some basic properties of gain graphs. We refer the reader to [5, 31, 32] for more details.

Let $G=(V,E)$ be a directed graph which may contain multiple edges and loops, and let ${{{\mathcal {S}}}}$ be a group. An ${{{\mathcal {S}}}}$-*gain graph*$(G,\phi )$ is a pair, in which each edge is associated with an element of ${{{\mathcal {S}}}}$ by a *gain function*$\phi :E\rightarrow {{{\mathcal {S}}}}$. The orientation of *G* is, in some sense, arbitrary, and is used only as a reference orientation: the orientation of each edge may be changed, provided that we also modify $\phi$ such that if the edge has gain *g* in one direction then it has gain $g^{-1}$ in the other direction. Therefore we often do not distinguish between *G* and the underlying undirected graph and use notations introduced in Sect. 1, implicitly referring to the underlying graph.

Let *W* be a walk in $(G,\phi )$. The *gain* of *W* is defined as $\phi (W)=\phi (e_1)\cdot \phi (e_2)\cdots \phi (e_k)$ if each edge is oriented in the forward direction through *W*, and for a backward edge $e_i$ we replace $\phi (e_i)$ with $\phi (e_i)^{-1}$ in the product. Note that $\phi (W^{-1})=\phi (W)^{-1}$.

Let $(G,\phi )$ be a gain graph. For $v\in V(G)$ we denote by $\pi _1(G,v)$ the set of closed walks starting at *v*. Similarly, for $X\subseteq E(G)$ and $v\in V(G)$, $\pi _1(X,v)$ denotes the set of closed walks starting at *v* and using only edges of *X*, where $\pi _1(X,v)=\emptyset$ if $v\notin V(X)$.

Let $X\subseteq E(G)$. The subgroup induced by *X* relative to *v* is defined as $\langle X\rangle _{\phi ,v}=\{\phi (W)\mid W\in \pi _1(X,v)\}$. The subscript $\phi$ of $\langle X\rangle _{\phi ,v}$ is sometimes omitted if it is clear from the context. Note that, for any connected $X\subseteq E(G)$ and two vertices $u,v\in V(X)$, $\langle X\rangle _{\psi ,u}$ is conjugate to $\langle X\rangle _{\psi ,v}$ (see, e.g., [5, p. 88] for the proof).

### The Switching Operation

For $v\in V(G)$ and $g\in {{{\mathcal {S}}}}$, a *switching operation atvwithg* changes the gain function $\phi$ on *E*(*G*) as follows.

1 $$\begin{aligned} \phi '(e)= {\left\{ \begin{array}{ll} g\cdot \phi (e)\cdot g^{-1} &{}\, \hbox {if } e \hbox { is a loop incident with } v, \\ g\cdot \phi (e) &{} \,\hbox {if } e \hbox { is a non-loop edge and is directed from } v, \\ \phi (e)\cdot g^{-1} &{}\, \hbox {if } e \hbox { is a non-loop edge and is directed to } v, \\ \phi (e) &{}\, \hbox {otherwise}. \end{array}\right. } \end{aligned}$$

We say that a gain function $\phi '$ on edge set *E*(*G*) is *equivalent* to another gain function $\phi$ on *E*(*G*) if $\phi '$ can be obtained from $\phi$ by a sequence of switching operations.

The following two facts are fundamental (see, e.g., [5, Sect.  2.5.2] or [31, Sect. 5] for the proofs).

#### **Proposition 2.1**

Let $(G,\phi )$ be a gain graph. Let $\phi '$ be the gain function obtained from $\phi$ by a switching operation. Then, for any $X\subseteq E(G)$ and $u\in V(G)$, $\langle X\rangle _{\phi ',u}$ is conjugate to $\langle X\rangle _{\phi ,u}$.

#### **Proposition 2.2**

Let $(G,\phi )$ be a gain graph. Then, for any forest $F\subseteq E(G)$, there is a gain function $\phi '$ equivalent to $\phi$ such that $\phi '(e)=\mathrm{id}$ for every $e\in F$.

### Balanced and Cyclic Sets of Edges

As we shall see, the subgroup $\langle X\rangle _{\psi ,v}$ itself will not be important, when we define our matroids induced by gains. We only need to know whether $\langle X\rangle _{\psi ,v}$ is trivial or not, or whether it is cyclic or not. We now introduce notions to describe these properties.

Let $(G,\phi )$ be a gain graph. An edge subset $F\subseteq E(G)$ is called *balanced* if $\langle F\rangle _{\psi ,v}$ is trivial for every $v\in V(F)$. Note that *F* is balanced if and only if every cycle in *F* is balanced. The latter property is the definition of the balancedness given by Zaslavsky [31].

In the same way, an edge subset $F\subseteq E(G)$ is called *cyclic* if $\langle F\rangle _{\psi ,v}$ is cyclic for every $v\in V(F)$ (note that the terms balanced and cyclic are not exclusive). A gain graph $(G,\phi )$ is called *balanced* (resp. *cyclic*) if *E*(*G*) is balanced (resp. cyclic), respectively.

Proposition 2.2 suggests a simple way to check the above introduced properties of *X*, in analogy with the fact that the cycle space of a graph is spanned by fundamental cycles. For a connected $X\subseteq E(G)$, take a spanning tree *T* of the edge induced graph *G*[*X*]. By Proposition 2.2 we can convert the gain function to an equivalent gain function such that $\phi (e)=\mathrm{id}$ for all $e\in T$. Now consider any closed walk $W\in \pi _1(X,v)$, and denote *W* by $W=v_1v_2, v_2v_3, \ldots , v_kv_{k+1}$, and let $W_i=P_i*\{v_iv_{i+1}\}*P_{i+1}^{-1}$ for $1\le i< k$, where $P_i$ denotes the path from *v* to $v_i$ in *T*. Then observe $\phi (W)=\phi (W_1)\cdot \phi (W_2)\cdots \phi (W_k)$. By $\phi (e)=\mathrm{id}$ for all $e\in T$, we deduce that $\phi (W)$ is a product of elements in $\{\phi (e):e\in X{\setminus } T\}$, implying that $\langle X\rangle _{\phi ,v}\subseteq \langle \phi (e):e\in X{\setminus } T\rangle$, where $\langle \phi (e):e\in X{\setminus } T\rangle$ is the group generated by $\{ \phi (e):e\in X{\setminus } T\}$. Conversely, $\phi (e)$ is contained in $\langle X\rangle _{\phi ,v}$ for all $e\in X{\setminus } T$. Thus, $\langle X\rangle _{\phi ,v}=\langle \phi (e):e\in X{\setminus } T\rangle$. In particular, we proved the following.

#### **Lemma 2.3**

For a connected $X\subseteq E(G)$ and a spanning tree *T* of *G*[*X*], suppose that $\phi (e)=\mathrm{id}$ for all $e\in T$. Then, $\langle X\rangle _{\phi ,v}=\langle \phi (e):e\in X{\setminus } T\rangle$. In particular, the following hold.

(i) *X* is unbalanced if and only if there is an edge in $X{\setminus } T$ whose gain is non-identity.
(ii) *X* is cyclic if and only if all gains of $X{\setminus } T$ are contained in a cyclic subgroup of ${{{\mathcal {S}}}}$.

The following technical lemmas will be used in the proof of our main theorem.

#### **Lemma 2.4**

Let $(G,\phi )$ be an ${{{\mathcal {S}}}}$-gain graph, and *X* and *Y* be connected edge subsets such that the graph $(V(X)\cap V(Y),X\cap Y)$ is connected.

1. If *X* and *Y* are balanced, then $X\cup Y$ is balanced.
2. If *X* is balanced and *Y* is cyclic, then $X\cup Y$ is cyclic.
3. If *X*, *Y* are cyclic and $X\cap Y$ is unbalanced, then $X\cup Y$ is cyclic, provided that for every non-trivial cyclic subgroup ${{{\mathcal {C}}}}$ of ${{{\mathcal {S}}}}$ there is a unique maximal cyclic subgroup $\bar{{{\mathcal {C}}}}$ of ${{{\mathcal {S}}}}$ containing ${{{\mathcal {C}}}}$.

#### *Proof*

Since the graph $(V(X)\cap V(Y), X\cap Y)$ is connected, there is a spanning tree *T* in $G[X\cup Y]$ such that $T\cap X$ is a spanning tree of *G*[*X*], $T\cap Y$ is a spanning tree of *G*[*Y*], and $T\cap X\cap Y$ is a spanning tree of $G[X\cap Y]$. By Proposition 2.2, there is a gain function $\phi '$ equivalent to $\phi$ such that $\phi '(e)=\mathrm{id}$ for each $e\in T$.

If *X* and *Y* are balanced, Lemma 2.3 implies that $\phi '(e)=\mathrm{id}$ for all $e\in X\cup Y$. Thus (1) holds.

If *X* is balanced, then every label in $X\cup Y$ is contained in $\langle Y\rangle _{\phi ',v}$ by Lemma 2.3, and hence $X\cup Y$ is cyclic if *Y* is cyclic. This implies (2).

If *X*, *Y* are cyclic and $X\cap Y$ is unbalanced, then there is an edge $e\in X\cap Y$ for which $\phi '(e)$ is non-identity. Let ${{{\mathcal {C}}}}$ be a cyclic subgroup of ${{{\mathcal {S}}}}$ generated by $\phi '(e)$ and $\bar{{{\mathcal {C}}}}$ be the maximal cyclic subgroup containing ${{{\mathcal {C}}}}$. Since *X* and *Y* are cyclic, Lemma 2.3 implies that $\phi '(e)\in \bar{{{\mathcal {C}}}}$ holds for every $e\in X$ and for every $e\in Y$. Therefore $X\cup Y$ is cyclic. $\square$

#### **Lemma 2.5**

Let $(G,\phi )$ be a gain graph, and *X* and *Y* be connected balanced edge subsets. If the number of connected components of the graph $(V(X)\cap V(Y),X\cap Y)$ is two, then $X\cup Y$ is cyclic.

#### *Proof*

We take a spanning tree *T* of $G[X\cup Y]$ such that $T\cap X$ is a spanning tree of *G*[*X*]. Since the number of connected components of $(V(X)\cap V(Y),X\cap Y)$ is two, $T\cap Y$ consists of two connected components, denoted $T_1$ and $T_2$. $\{V(T_1), V(T_2)\}$ partitions *Y* into three subsets $\{Y_1,Y_2,Y_3\}$ such that $Y_i=\{e\in Y:V(\{e\})\subseteq V(T_i)\}$ for $i=1,2$ and $Y_3=Y{\setminus } (Y_1\cup Y_2)$.

By Proposition 2.2, we can take a gain function $\phi '$ equivalent to $\phi$ such that $\phi '(e)=\mathrm{id}$ for $e\in T$. Since *X* and *Y* are balanced, we have $\phi '(e)=\mathrm{id}$ for $e\in X\cup Y_1\cup Y_2$. Moreover, assuming that every edge in $Y_3$ is oriented toward $V(Y_1)$, we have $\phi '(e)=\phi '(f)$ for all $e,f\in Y_3$, since otherwise $T_1\cup T_2\cup \{e,f\}$ contains an unbalanced cycle, contradicting the fact that *Y* is balanced. Therefore $X\cup Y$ is cyclic. $\square$

## Gain Count Matroids

### Matroids Induced by Submodular Functions

Let *E* be a finite set. A function $\mu :2^E\rightarrow {\mathbb {R}}$ is called *submodular* if $\mu (X)+\mu (Y)\ge \mu (X\cup Y)+\mu (X\cap Y)$ for every $X,Y\subseteq E$. $\mu$ is *monotone* if $\mu (X)\le \mu (Y)$ for any $X\subseteq Y$. A monotone submodular function $\mu :2^E\rightarrow {\mathbb {Z}}$*induces* a matroid on *E*, where $F\subseteq E$ is independent if and only if $|I|\le \mu (I)$ for every nonempty $I\subseteq F$. See e.g. [4, Sect. 13.4]. This matroid is denoted by ${{{\mathcal {M}}}}(\mu )$.

For a monotone submodular function $\mu$, let $\nu =\mu -1$. Then, $\nu$ is monotone submodular and induces the matroid ${{{\mathcal {M}}}}(\nu )$. This matroid is referred to as the *Dilworth truncation* of ${{{\mathcal {M}}}}(\mu )$. Although the details are omitted here, the name of Dilworth truncation is justified from a connection with Dilworth truncation for general matroids, see [4] for more details.

Now we consider the union of two matroids induced by monotone submodular functions $\mu _1$ and $\mu _2$. Since monotonicity and submodularity are both preserved under the sum operation, $\mu _1+\mu _2$ is monotone and submodular. In general, the union of ${{{\mathcal {M}}}}(\mu _1)$ and ${{{\mathcal {M}}}}(\mu _2)$ is not equal to ${{{\mathcal {M}}}}(\mu _1+\mu _2)$. We do have equality in some special cases, for example, when $\mu _1=\mu _2$ or when both $\mu _1$ and $\mu _2$ are nonnegative.

As an example, consider the union of two copies of the graphic matroid of a graph $G=(V,E)$. It is the matroid induced by $f_{2,2}$ defined by $f_{2,2}(F)=2|V(F)|-2$ on $2^E$, as $f_{2,2}/2$ induces the graphic matroid on *G*. The 2-dimensional generic rigidity matroid is the one induced by $f_{2,2}-1$, and hence it is the Dilworth truncation of the union of two copies of the graphic matroid.

In general, for a graph $G=(V,E)$ and two integers *k* and *l* with $k\ge 1$ and $l\le 2k-1$, let

$$\begin{aligned} f_{k,l}(F)=k|V(F)|-l \quad (F\subseteq E). \end{aligned}$$

*G* is called (*k*, *l*)-*sparse* if $|F|\le f_{k,l}(F)$ for any nonempty $F\subseteq E$. The matroid induced by $f_{k,l}$ is called the (*k*, *l*)-*count matroid* on *G*. If $l\ge 0$, ${{{\mathcal {M}}}}(f_{k,l})$ is indeed the one induced by $f_{k,0}$, truncated *l* times. See e.g. [4] for more details. Below we shall apply the same construction to the union of some copies of a frame matroid to define gain-count matroids.

### Gain-Count Matroids

In this paper we shall consider frame matroids on gain graphs. Let ${{{\mathcal {S}}}}$ be a group and $(G,\phi )$ be an ${{{\mathcal {S}}}}$-gain graph. The *frame matroid* of $(G,\phi )$ is defined such that $F\subseteq E$ is independent if and only if each connected component of *F* contains no cycle or just one cycle, which is unbalanced if it exists [32]. If we define $g_{{{\mathcal {S}}}}:2^E\rightarrow {\mathbb {Z}}$ by

2 $$\begin{aligned} g_{{{\mathcal {S}}}}(F)=\sum _{F_i\in C(F)}(|V(F_i)|-1+\alpha _{{{\mathcal {S}}}}(F_i)), \end{aligned}$$

where

3 $$\begin{aligned} \alpha _{{{\mathcal {S}}}}(F)= {\left\{ \begin{array}{ll} 1 &{}\quad \hbox {if }F\hbox { is unbalanced}, \\ 0 &{}\quad \hbox {otherwise}, \end{array}\right. } \end{aligned}$$

then the frame matroid is the matroid induced by $g_{{{\mathcal {S}}}}$. We omit the subscript ${{{\mathcal {S}}}}$ from $\alpha _{{{\mathcal {S}}}}$ if it is clear from the context.

For an ${{{\mathcal {S}}}}$-gain graph and two positive integers *k* and *l* with $k\le l$, we define $g_{k,l}:2^E\rightarrow {\mathbb {Z}}$ by

4 $$\begin{aligned} g_{k,l}(F)=kg_{{{\mathcal {S}}}}(F)-(l-k) \quad (F\subseteq E). \end{aligned}$$

We call the matroid ${{{\mathcal {M}}}}(g_{k,l})$ induced by $g_{k,l}$ a (*k*, *l*)-*gain-count matroid* or *g-count matroid* for short. This matroid is the union of *k* copies of the frame matroid, followed by $l-k$ Dilworth truncations. In this paper, we shall investigate the (2, 3)-g-count matroid and its variants.

The independence of ${{{\mathcal {M}}}}(g_{k,l})$ can be described in a compact form (see [8] for the proof, which is a rather straightforward calculation).

#### **Lemma 3.1**

Let $(G,\phi )$ be an ${{{\mathcal {S}}}}$-gain graph with $G=(V,E)$. Then *E* is independent in ${{{\mathcal {M}}}}(g_{k,l})$ if and only if $|F|\le k|V(F)|-l+k\alpha (F)$ for any nonempty $F\subseteq E$.

In this sense, we may define (*k*, *l*)-*gain-sparsity* as in the case of (*k*, *l*)-sparsity of undirected graphs as follows.

#### **Definition 3.1**

Let *k* and *l* be positive integers with $k\le l$ and $(G,\phi )$ be an ${{{\mathcal {S}}}}$-gain graph with a graph $G=(V,E)$ and a group ${{{\mathcal {S}}}}$. An edge set $X\subseteq E$ is called (*k*, *l*)-*gain-sparse* (or (*k*, *l*)-*g-sparse* for short) if $|F|\le g_{k,l}(F)$ for any nonempty $F\subseteq X$, i.e.,

- $|F|\le k|V(F)|-l$ for every nonempty balanced $F\subseteq X$;
- $|F|\le k|V(F)|-l+k$ for every nonempty unbalanced $F\subseteq X$,

and it is called (*k*, *l*)-*gain-tight* (or (*k*, *l*)-*g-tight* for short) if it is (*k*, *l*)-g-sparse with $|X|=g_{k,l}(X)$.

If *E* is (*k*, *l*)-g-sparse then graph $(G,\phi )$ is said to be (*k*, *l*)-*g-sparse*, and $(G,\phi )$ is called *maximum* (*k*, *l*)-*g-tight* if it is (*k*, *l*)-g-sparse with $|E|=k|V|-l+k$.

#### *Remark 3.1*

Note that the value of $g_{k,l}$ is invariant under switching operations, and thus the induced matroid is uniquely determined up to equivalence of gain functions.

#### *Remark 3.2*

We can further consider the union of frame matroids of gain graphs $(G,\phi _1)$ and $(G,\phi _2)$ with the same underlying graph but distinct gain functions. We should remark that both graphic matroids and bicircular matroids are special cases of frame matroids. The union of copies of graphic, frame and bicircular matroids on an ${{{\mathcal {S}}}}$-gain graph, followed by Dilworth truncations, can be described as the matroid induced by a counting condition. For example, in the union of the graphic matroid and the frame matroid of a gain graph $(G,\phi )$, followed by a single Dilworth truncation, *E*(*G*) is independent if and only if $|F|\le 2|V(F)|-3$ for any balanced set $F\subseteq E(G)$ and $|F|\le 2|V(F)|-2$ for any nonempty $F\subseteq E(G)$. This matroid was used by Ross [17] for characterizing the generic rigidity of bar-and-joint frameworks on a torus. Tanigawa [24] proposed a more general class of matroids extending matroid union operations.

## Constructive Characterization of Maximum (2, 3)-g-Tight Graphs

### Operations Preserving (2, 3)-g-Sparsity

In this section we define three operations, called *extensions*, that preserve (2, 3)-g-sparsity. The first two operations generalize the well-known Henneberg operations [26, 28] to gain graphs.

Let $(G,\phi )$ be an ${{{\mathcal {S}}}}$-gain graph. The 0*-extension* adds a new vertex *v* and two new non-loop edges $e_{1}$ and $e_{2}$ to *G* such that the new edges are incident to *v* and the other endvertices are two not necessarily distinct vertices of *V*(*G*). If $e_{1}$ and $e_{2}$ are not parallel then their labels can be arbitrary. Otherwise the labels are assigned such that $\phi (e_{1})\ne \phi (e_{2})$, assuming that $e_1$ and $e_2$ are directed to *v*.

The 1*-extension* first chooses an edge *e* and a vertex *z*, where *e* may be a loop and *z* may be an endvertex of *e*. It subdivides *e*, with a new vertex *v* and new edges $e_{1},e_{2}$ such that the tail of $e_1$ is the tail of *e* and the tail of $e_2$ is the head of *e*. The labels of the new edges are assigned such that $\phi (e_{1})\cdot \phi (e_{2})^{-1}=\phi (e)$. The 1-extension also adds a third edge $e_{3}$ oriented to *v*. The label of $e_3$ is assigned so that it is *locally unbalanced*, i.e., every two-cycle $e_ie_j$, if it exists, is unbalanced.

**Fig. 1:**
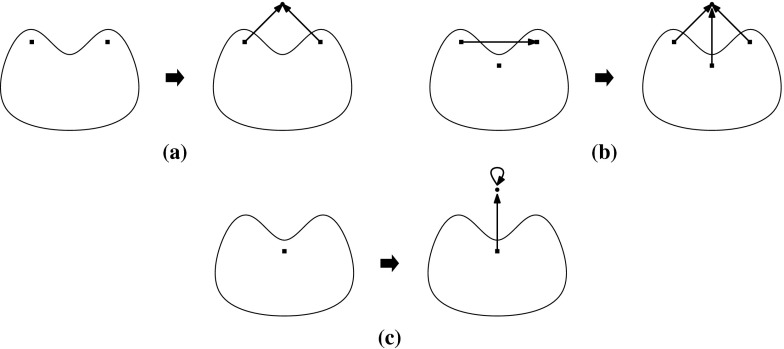
**a** 0-Extension, where the new edges may be parallel. **b** 1-Extension, where the removed edge may be a loop and the new edges may be parallel. **c** Loop-1-extension

The *loop *1*-extension* adds a new vertex *v* to *G* and connects it to a vertex $z\in V(G)$ by a new edge with any label. It also adds a new loop *l* incident to *v* with $\phi (l)\ne \mathrm {id}$ (Fig. 1).

The 0-extension and the 1-extension were already considered by Ross [17] for ${\mathbb {Z}}^2$-gain graphs. In the covering graph each operation can be seen as a graph operation that preserves the underlying symmetry. Some of them can be recognized as performing so-called Henneberg operations [26, 28] simultaneously. In the case of three-fold rotation symmetry, these operations are considered by Schulze [21].

#### **Lemma 4.1**

Let $(G,\phi )$ be a (2,3)-g-sparse graph. Applying the 0-extension, 1-extension or loop 1-extension to $(G,\phi )$ results in a (2,3)-g-sparse graph $(G',\phi ')$ with $|V(G')|=|V(G)|+1$ and $|E(G')|=|E(G)|+2$.

#### *Proof*

For a contradiction, suppose that $G'$ contains an edge set $F\subseteq E(G')$ for which $|F|>2|V(F)|-3+2\alpha (F)$. Let *v* be the new vertex added by the extension, and let $E_v$ be the set of edges incident to *v*. Since $E(G'){\setminus } E_v\subseteq E(G)$, $E_v\cap F\ne \emptyset$. In particular, $v\in V(F)$. Also, since the new labeling is assigned to be locally unbalanced, *F* is not contained in $E_v$.

If $G'$ is constructed by a 1-extension then let *e* be the subdivided edge of *G* and let $e_1$ and $e_2$ be the resulting two new edges.

Let $F'=F{\setminus } E_v$. If $G'$ is constructed by a 1-extension and $\{e_1,e_2\}\subseteq F$, then we further insert *e* to $F'$. We then have $|F'|\ge |F|-2$, $|V(F')|=|V(F)|-1$, and $\alpha (F')\le \alpha (F)$ in each case. These imply $|F'|\ge |F|-2>2|V(F)|-5+2\alpha (F)\ge 2|V(F')|-3+2\alpha (F')$, contradicting the (2, 3)-g-sparsity of *G* as $\emptyset \ne F'\subseteq E(G)$. $\square$

We shall define the inverse moves of the operations above, which are called *reductions*. For a vertex *v* and two incoming non-loop edges $e_1=(u,v)$ and $e_2=(w,v)$, we denote by $e_1\cdot e_2^{-1}$ a new edge from *u* to *w* with label $\phi (e_1)\cdot \phi (e_2)^{-1}$ (by extending $\phi$). If $u=w$ then $e_1\cdot e_2^{-1}$ is a loop. Each reduction corresponds to one of the following operations on a gain graph $(G,\phi )$.

A 0*-reduction* chooses a degree two vertex and deletes it from *G*.

A 1*-reduction* chooses a vertex *v* with $d(v)=3$ that is not incident to a loop. Let $e_1,e_2,e_3$ be the edges incident to *v*. Without loss of generality we may assume that each $e_i$ is oriented to *v*. The 1-reduction deletes *v* with the incident edges and adds one of $e_1\cdot e_2^{-1}$, $e_2\cdot e_3^{-1}$ and $e_3\cdot e_1^{-1}$ as a new edge.

A *loop *1*-reduction* chooses a vertex incident to exactly one loop and one non-loop edge and deletes the chosen vertex with the incident edges.

A 1-reduction may destroy the (2, 3)-g-sparsity of a graph. We say that a reduction (at a vertex *v*) is *admissible* if the resulting graph is (2, 3)-g-sparse.

### Constructive Characterization

#### **Lemma 4.2**

Let $(G,\phi )$ be a (2,3)-g-sparse graph and $v\in V(G)$ a vertex not incident to a loop with $d(v)=3$. Then there is an admissible 1-reduction at *v*.

#### *Proof*

Let $E=E(G)$, $G'=G-v$ and $E'=E(G')$. Let $e_1,e_2,e_3$ be the edges incident to *v* in *G*. Without loss of generality we may assume that each $e_i$ is oriented to *v*. For simplicity we put $e_{i,j}=e_i\cdot e_j^{-1}$.

Suppose for a contradiction that there is no admissible splitting at *v*, that is, none of $E'+e_{1,2}$, $E'+e_{2,3}$ and $E'+e_{3,1}$ is independent in ${\mathcal {M}}(g_{2,3})$. Equivalently, $e_{1,2},e_{2,3},e_{3,1}\in \mathrm {cl}_{g}(E')$, where $\mathrm {cl}_{g}$ denotes the closure operator of ${{{\mathcal {M}}}}(g_{2,3})$. Let $X=\{e_1,e_2,e_3,e_{1,2},e_{2,3},e_{3,1}\}$.

#### **Claim 4.3**

$$e_1\in \mathrm{cl}_g(X-e_1)$$.

#### *Proof*

We split the proof into three cases depending on the cardinality of *N*(*v*).

If $|N(v)|=3$ then, by Proposition 2.2, we may assume $\phi (e_1)=\phi (e_2)=\phi (e_3)=\mathrm {id}$. We then have $\phi (e_{1,2})=\phi (e_{2,3})=\phi (e_{3,1})=\mathrm {id}$. Therefore *X* forms a balanced $K_4$, which is a circuit of ${{{\mathcal {M}}}}(g_{2,3})$. Thus, $e_1\in \mathrm{cl}_g(X-e_1)$ holds.

If $|N(v)|=2$ then we may assume that $e_1$ and $e_2$ are parallel. By Proposition 2.2, we may assume that $\phi (e_2)=\phi (e_3)=\mathrm{id}$. This implies $\phi (e_{1,3})=\phi (e_1)$ and $\phi (e_{2,3})=\mathrm{id}$. Since *G* is (2, 3)-g-sparse, we have $\phi (e_1)\ne \mathrm{id}$ by $\phi (e_2)=\phi (e_3)=\mathrm{id}$, which implies that $e_{1,2}$ is an unbalanced loop with $\phi (e_{1,2})=\phi (e_1)$. It can be easily checked, by counting, that *X* is indeed a circuit in ${{{\mathcal {M}}}}(g_{2,3})$. Thus, $e_1\in \mathrm{cl}_g(X-e_1)$ holds.

If $|N(v)|=1$ then let $X'=\{e_1,e_2,e_3,e_{1,2}\}$. We have $|X'|=2|V(X')|$ and $X'$ is a circuit of ${{{\mathcal {M}}}}(g_{2,3})$. Therefore $e_1\in \mathrm{cl}_g(X'-e_1)\subset \mathrm{cl}_g(X-e_1)$. $\square$

Since $e_{1,2},e_{2,3},e_{3,1}\in \mathrm {cl}_g(E')$, by Claim 4.3, we have $e_1\in \mathrm {cl}_g(X-e_1)\subseteq \mathrm {cl}_g(E'+X-e_1)=\mathrm {cl}_g(E'+e_2+e_3)=\mathrm {cl}_g(E-e_1)$, which contradicts the (2, 3)-g-sparsity of *G*. $\square$

The following constructive characterization of maximum (2, 3)-g-tight graphs is a direct consequence of Lemmas 4.1 and 4.2 (see [8] for the concrete proof).

#### **Theorem 4.4**

An ${{{\mathcal {S}}}}$-gain graph $(G,\phi )$ is maximum (2,3)-g-tight if and only if it can be built up from an ${{{\mathcal {S}}}}$-gain graph with one vertex and an unbalanced loop incident to it with a sequence of 0-extensions, 1-extensions, and loop-1-extensions.

#### *Remark 4.1*

Theorem 4.4 for the case of three-fold rotation symmetry is implicit in [20]. For ${\mathbb {Z}}^2$-gain graphs, the corresponding result with a slightly different count condition (see Remark 3.2) was shown by Ross [17].

Further applications of Theorem 4.4 and other operations are recently discussed in [14, 15, 22].

## Symmetry-Forced Rigidity

In this section we define the notion of symmetry-forced infinitesimal rigidity, introduced by Schulze and Whiteley [23]. In Sect. 5.1, we first introduce ${{{\mathcal {S}}}}$-symmetric graphs, whose automorphism group has a subgroup isomorphic to ${{{\mathcal {S}}}}$. In Sect. 5.2 we shall review the conventional notion of infinitesimal rigidity. In Sect. 5.3 we introduce symmetry-forced infinitesimal rigidity, which is only concerned with infinitesimal motions invariant under the underlying symmetry. In Sect. 5.4 we introduce the orbit rigidity matrix, which is the main tool for investigating symmetry-forced infinitesimal rigidity in the subsequent sections. In Sect. 5.5 we prove a necessary condition for symmetric frameworks to be symmetry-forced infinitesimally rigid.

### ${{{\mathcal {S}}}}$-Symmetric Graphs

Let *H* be a simple graph. An *automorphism* of *H* is a permutation $\pi :V(H)\rightarrow V(H)$ such that $\{u,v\}\in E(H)$ if and only if $\{\pi (u),\pi (v)\}\in E(H)$. The set of all automorphisms of *H* forms a subgroup of the symmetric group of *V*(*H*), known as the *automorphism group*$\mathrm{Aut}(H)$ of *H*.

Let ${{{\mathcal {S}}}}$ be a group. An *action* of ${{{\mathcal {S}}}}$ on *H* is a group homomorphism $\rho :{{{\mathcal {S}}}}\rightarrow \mathrm{Aut}(H)$. An action $\rho$ is called *free* if $\rho (g)(v)\ne v$ for any $v\in V$ and any non-identity $g\in {{{\mathcal {S}}}}$. We say that a graph *H* is $({{{\mathcal {S}}}},\rho )$-*symmetric* if ${{{\mathcal {S}}}}$ acts on *H* by $\rho$. If $\rho$ is clear from the context, we will simply denote $\rho (g)(v)$ by $g\cdot v$ or *gv*. Note that, for $g\in {{{\mathcal {S}}}}$ and $u,v\in V$, $\{u,v\}\in E(H)$ if and only if $\{gu,gv\}\in E(H)$, and hence there is an induced action of ${{{\mathcal {S}}}}$ on *E*(*H*) defined by $g\cdot \{u,v\}=\{gu,gv\}$.

**Fig. 2:**
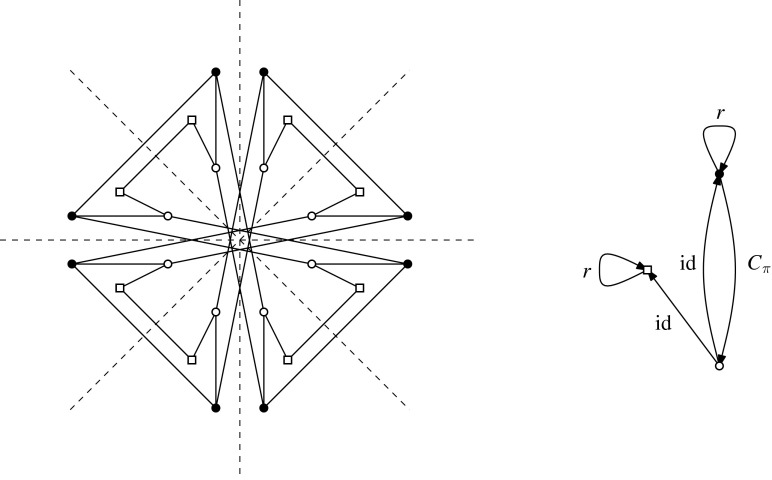
A ${{{\mathcal {D}}}}_8$-symmetric graph and the quotient gain graph

Let *H* be an $({{{\mathcal {S}}}},\rho )$-symmetric graph. The *quotient graph*$H/{{{\mathcal {S}}}}$ of *H* is a multigraph on the set $V(H)/{{{\mathcal {S}}}}$ of vertex orbits, together with the set $E(H)/{{{\mathcal {S}}}}$ of edge orbits as the edge set. An edge orbit may be represented by a loop in $H/{{{\mathcal {S}}}}$. Figure 2 is an example when ${{{\mathcal {S}}}}$ is a dihedral group.

Different graphs may have the same quotient graph. However, if we assume that $\rho$ is free, then a gain labeling makes the relation one-to-one. To see this, we arbitrarily choose a vertex *v* as a representative vertex from each vertex orbit. Then, each orbit is written by ${{{\mathcal {S}}}}v=\{gv:g\in {{{\mathcal {S}}}}\}$. If $\rho$ is a free action, an edge orbit connecting ${{{\mathcal {S}}}}u$ and ${{{\mathcal {S}}}}v$ in $H/{{{\mathcal {S}}}}$ can be written by $\{\{gu,ghv\}:g\in {{{\mathcal {S}}}}\}$ for a unique $h\in {{{\mathcal {S}}}}$. We then orient the edge orbit from ${{{\mathcal {S}}}}u$ to ${{{\mathcal {S}}}}v$ in $H/{{{\mathcal {S}}}}$ and assign to it the gain *h*. In this way, we obtain *the quotient*${{{\mathcal {S}}}}$-*gain graph*, denoted $(H/{{{\mathcal {S}}}},\phi )$.

Conversely, any ${{{\mathcal {S}}}}$-gain graph $(G,\phi )$ can be “lifted” as an $({{{\mathcal {S}}}},\rho )$-symmetric graph with a free action $\rho$. To see this, we simply denote the pair (*g*, *v*) of $g\in {{{\mathcal {S}}}}$ and $v\in V(G)$ by *gv*. The *covering graph* (also known as the derived graph) of $(G,\phi )$ is the simple graph with vertex set ${{{\mathcal {S}}}}\times V(G)=\{gv:g\in {{{\mathcal {S}}}}, v\in V(G)\}$ and the edge set $\{\{gu,g\phi (e)v\}:e=(u,v)\in E(G), g\in {{{\mathcal {S}}}}\}$. Clearly, ${{{\mathcal {S}}}}$ freely acts on the covering graph, under which the quotient gain graph comes back to $(G,\phi )$. For more properties of covering graphs, see e.g. [5].

### Infinitesimal Rigidity

Before we investigate the rigidity theory of symmetric graphs we review the basic notions of the conventional rigidity of graphs.

A *d*-*dimensional bar-and-joint framework* (or simply a framework) is a pair (*H*, *p*) of a simple graph *H* and a mapping $p:V(H)\rightarrow {\mathbb {R}}^d$, called a *joint-configuration*. We denote the set $\{p(v):v\in V(H)\}$ of points by *p*(*H*).

Infinitesimal rigidity is concerned with the dimension of the space of infinitesimal motions. An *infinitesimal motion* of a framework (*H*, *p*) is defined as an assignment $m:V(H)\rightarrow {\mathbb {R}}^d$ such that

5 $$\begin{aligned} \langle m(u)-m(v), p(u)-p(v)\rangle =0 \quad \hbox {for all } \{u,v\}\in E(H), \end{aligned}$$

where $\langle \cdot , \cdot \rangle$ denotes the standard inner product in the *d*-dimensional Euclidean space. The set of infinitesimal motions forms a linear space, denoted *L*(*H*, *p*).

In general, for a set $P\subseteq {\mathbb {R}}^d$ of points, an *infinitesimal isometry* of *P* is defined by $m:P\rightarrow {\mathbb {R}}^d$ such that

$$\begin{aligned} \langle m(x)-m(y), x-y \rangle =0 \quad \hbox {for all } x, y\in P. \end{aligned}$$

The set of infinitesimal isometries forms a linear space, denoted by $\mathrm{iso}(P)$. Notice that, for a skew-symmetric matrix *S* and $t\in {\mathbb {R}}^d$, a mapping $m:P\rightarrow {\mathbb {R}}^d$ defined by

$$\begin{aligned} m(x)=Sx+t \quad (x\in P) \end{aligned}$$

is an infinitesimal isometry of *P*. Indeed, it is well-known that any infinitesimal isometry can be described in this form, and

6 $$\begin{aligned} \dim \mathrm{iso}(P)=d(k+1)-\left( \begin{array}{c} k+1 \\ 2 \end{array} \right) , \end{aligned}$$

where *k* denotes the affine dimension of *P*.

#### *Example 5.1*

Let us consider the infinitesimal isometries of a point set *P* in the plane. According to (6), we have

$$\begin{aligned} \dim \mathrm{iso}(P)= {\left\{ \begin{array}{ll} 3 &{} \quad \hbox {if } |P|\ge 2, \\ 2 &{} \quad \hbox {if } |P|=1. \end{array}\right. } \end{aligned}$$

**Fig. 3:**
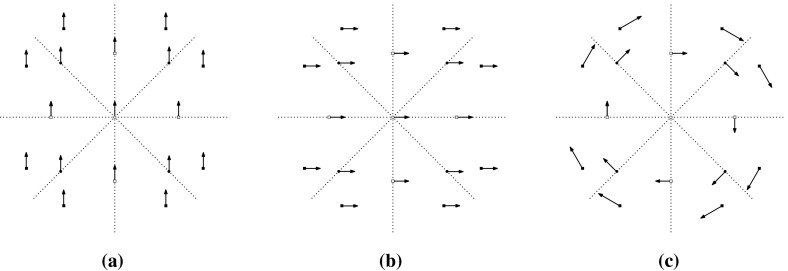
Three independent infinitesimal isometries in the plane, among which **a** is symmetric with respect to the group of a vertical reflection, **b** is symmetric with respect to the group of a horizontal reflection, and **c** is symmetric with respect to the group of rotations

For $t\in {\mathbb {R}}^2$, let $m_t(x)=t \ (x\in P)$. Then, $m_t$ is an infinitesimal isometry, called a *translation*. On the other hand, let $m_r(x)=C_{\pi /2}x \ (x\in P)$, where $C_{\pi /2}$ denotes the $2\times 2$ orthogonal matrix representing the four-fold rotation around the origin. Then, $m_r$ is also an infinitesimal isometry, which we call an *infinitesimal rotation*. It is well known that $\mathrm{iso}(P)$ is spanned by $\{m_{t}, m_{t'}, m_r\}$ for two linearly independent vectors $t,t'\in {\mathbb {R}}^2$. See Fig. 3 for examples.

An infinitesimal motion $m:V(H)\rightarrow {\mathbb {R}}^d$ of a framework (*H*, *p*) is said to be *trivial* if *m* can be expressed by

7 $$\begin{aligned} m(v)=Sp(v)+t \quad (v\in V(H)) \end{aligned}$$

for some skew-symmetric matrix *S* and $t\in {\mathbb {R}}^d$. The set of all trivial motions forms a linear subspace of *L*(*H*, *p*), denoted by $\mathrm{tri}(H,p)$. By definition, $\mathrm{tri}(H,p)$ is isomorphic to $\mathrm{iso}(p(H))$, and hence (6) gives the exact dimension of $\mathrm{tri}(H,p)$. (*H*, *p*) is called *infinitesimally rigid* if $L(H,p)=\mathrm{tri}(H,p)$.

### Symmetric Frameworks and Symmetry-Forced Infinitesimal Rigidity

A *discrete point group* (or simply a *point group*) is a finite discrete subgroup of ${{{\mathcal {O}}}}({\mathbb {R}}^d)$, the *orthogonal group* of dimension *d*, i.e., the set of $d\times d$ orthogonal matrices over ${\mathbb {R}}$. For $d=2$, point groups are classified into two classes, *groups*${{{\mathcal {C}}}}_k$*ofk*-*fold rotations* and *dihedral groups*${{{\mathcal {D}}}}_{2k}$ of order 2*k*. For a special case, ${{{\mathcal {D}}}}_2$ consists of a mirror-reflection and the identity. In the subsequent discussion of this section, ${{{\mathcal {S}}}}$ denotes a point group.

Suppose that *H* is $({{{\mathcal {S}}}},\rho )$-symmetric for a point group ${{{\mathcal {S}}}}$. A joint-configuration *p* is said to be $({{{\mathcal {S}}}},\rho )$-*symmetric* (or, simply, ${{{\mathcal {S}}}}$-symmetric) if

8 $$\begin{aligned} gp(v)=p(gv) \quad \hbox {for all } g\in {{{\mathcal {S}}}} \hbox { and for all } v\in V(H). \end{aligned}$$

Such a pair (*H*, *p*) is called an $({{{\mathcal {S}}}},\rho )$-*symmetric framework* (or simply an ${{{\mathcal {S}}}}$-symmetric framework or a symmetric framework).

We shall consider “symmetry-preserving” infinitesimal motions of symmetric frameworks. We say that an infinitesimal motion $m:V(H)\rightarrow {\mathbb {R}}^d$ is *symmetric* if

9 $$\begin{aligned} gm(v)=m(gv) \quad \hbox {for all } g\in {{{\mathcal {S}}}} \hbox { and for all } v\in V(H). \end{aligned}$$

The set of ${{{\mathcal {S}}}}$-symmetric infinitesimal motions and the set of trivial ones form linear subspaces of *L*(*H*, *p*) and $\mathrm{tri}(H,p)$, denoted $L_{{{\mathcal {S}}}}(H,p)$ and $\mathrm{tri}_{{{\mathcal {S}}}}(H,p)$, respectively. We say that (*H*, *p*) is *symmetry-forced infinitesimally rigid* if $L_{{{\mathcal {S}}}}(H,p)=\mathrm{tri}_{{{\mathcal {S}}}}(H,p)$.

A set *P* of points is called ${{{\mathcal {S}}}}$-*symmetric* if $gP=\{gp:p\in P\}=P$ for all $g\in {{{\mathcal {S}}}}$. An infinitesimal isometry $m:P\rightarrow {\mathbb {R}}^d$ of an ${{{\mathcal {S}}}}$-symmetric point set *P* is called ${{{\mathcal {S}}}}$-*symmetric* if $gm(x)=m(gx)$ for all $x\in P$ and $g\in {{{\mathcal {S}}}}$. The set of ${{{\mathcal {S}}}}$-symmetric infinitesimal isometries forms a linear subspace of $\mathrm{iso}(P)$, denoted $\mathrm{iso}_{{{\mathcal {S}}}}(P)$. Clearly, $\mathrm{tri}_{{{\mathcal {S}}}}(H, p)$ is isomorphic to $\mathrm{iso}_{{{\mathcal {S}}}}(p(H))$.

#### *Example 5.2*

Let us consider point groups in ${{{\mathcal {O}}}}({\mathbb {R}}^2)$, which will be mainly discussed in Sects. 6 and 8. Let *P* be an ${{{\mathcal {S}}}}$-symmetric point set in ${\mathbb {R}}^2$. See Fig. 3 for examples of ${{{\mathcal {C}}}}_k$-symmetric infinitesimal isometries. In general, if $|P|>1$,

$$\begin{aligned} \dim \mathrm{iso}_{{{{\mathcal {C}}}}_k}(P)= {\left\{ \begin{array}{ll} 3 &{}\quad \hbox {if } k=1, \\ 1 &{} \quad \hbox {if } k\ge 2, \end{array}\right. } \end{aligned}$$

and if $$P=\{x\}$$,

$$\begin{aligned} \dim \mathrm{iso}_{{{{\mathcal {C}}}}_k}(P)= {\left\{ \begin{array}{ll} 2 &{}\quad \hbox {if } k=1, \\ 0 &{}\quad \hbox {if } k\ge 2 \hbox { (where }x\hbox { should be the origin).} \end{array}\right. } \end{aligned}$$

Similarly, for the dihedral group ${{{\mathcal {D}}}}_{2k}$ of order 2*k*,

$$\begin{aligned} \dim \mathrm{iso}_{{{{\mathcal {D}}}}_{2k}}(P)= {\left\{ \begin{array}{ll} 1 &{}\quad \hbox {if } k=1, \\ 0 &{}\quad \hbox {if } k\ge 2. \end{array}\right. } \end{aligned}$$

A result of Schulze [21] motivates us to look at ${{{\mathcal {S}}}}$-symmetric infinitesimal rigidity, which states that if (*H*, *p*) is not symmetry-forced infinitesimally rigid on an ${{{\mathcal {S}}}}$-generic *p*, then (*H*, *p*) has a nontrivial continuous motion that preserves the $({{{\mathcal {S}}}},\rho )$-symmetry.

### The Orbit Rigidity Matrix

Let (*H*, *p*) be an $({{{\mathcal {S}}}},\rho )$-symmetric framework in ${\mathbb {R}}^d$. Due to (9), the system (5) of linear equations (with respect to *m*) is redundant. Schulze and Whiteley [23] pointed out that the system can be reduced to $|E(H)/{{{\mathcal {S}}}}|$ linear equations.

To see this, we first define a *joint-configuration*${\tilde{p}}$ of vertex orbits by ${\tilde{p}}:V(H)/{{{\mathcal {S}}}}\rightarrow {\mathbb {R}}^d$. By taking a representative vertex *v* from each vertex orbit ${{{\mathcal {S}}}}v$, we set ${\tilde{p}}({{{\mathcal {S}}}}v)=p(v)$ [then, the locations of the other non-representative vertices are uniquely determined by (8)].

In a similar way, we define an *infinitesimal motion* of $(H/{{{\mathcal {S}}}},{\tilde{p}})$ by ${\tilde{m}}:V(H)/{{{\mathcal {S}}}}\rightarrow {\mathbb {R}}^d$. By using the representative vertices determined above, we fix a one-to-one correspondence between ${{{\mathcal {S}}}}$-symmetric infinitesimal motions of *V*(*H*) and infinitesimal motions of $V(H)/{{{\mathcal {S}}}}$ by ${\tilde{m}}({{{\mathcal {S}}}}v)=m(v)$ for each vertex orbit ${{{\mathcal {S}}}}v$.

Let $(H/{{{\mathcal {S}}}},\phi )$ be the quotient ${{{\mathcal {S}}}}$-gain graph of *H*. Recall that each (oriented) edge orbit ${{{\mathcal {S}}}}e$ connecting ${{{\mathcal {S}}}}u$ and ${{{\mathcal {S}}}}v$ with gain $h_e$ can be written by ${{{\mathcal {S}}}}e=\{\{gu,gh_e v\}:g\in {{{\mathcal {S}}}}\}$. The system (5) is hence written by

10 $$\begin{aligned} \langle m(gu)-m(gh_ev), p(gu)-p(gh_ev)\rangle =0 \quad \hbox {for all } \{gu,gh_ev\}\in {{{\mathcal {S}}}}e \end{aligned}$$

over all edge orbits ${{{\mathcal {S}}}}e\in E(H)/{{{\mathcal {S}}}}$. Recall that the transpose of *g* is $g^{-1}$ for any $g\in {{{\mathcal {O}}}}({\mathbb {R}}^d)$. By (8) and (9),

$$\begin{aligned}&\langle m(gu)-m(gh_ev), p(gu)-p(gh_ev)\rangle \\&\quad =\langle m(u)-h_em(v), p(u)-h_ep(v) \rangle \\&\quad =\langle m(u), p(u)-h_e p(v) \rangle + \langle m(v), p(v)-h_e^{-1}p(u)\rangle \\&\quad =\langle {\tilde{m}}({{{\mathcal {S}}}}u), {\tilde{p}}({{{\mathcal {S}}}}u)-h_e {\tilde{p}}({{{\mathcal {S}}}}v) \rangle + \langle {\tilde{m}}({{{\mathcal {S}}}}v), {\tilde{p}}({{{\mathcal {S}}}}v)-h_e^{-1}{\tilde{p}}({{{\mathcal {S}}}}u)\rangle . \end{aligned}$$

Therefore, for ${\tilde{p}}:V(H)/{{{\mathcal {S}}}}\rightarrow {\mathbb {R}}^d$, a mapping ${\tilde{m}}:H/{{{\mathcal {S}}}}\rightarrow {\mathbb {R}}^d$ is an infinitesimal motion of $(H/{{{\mathcal {S}}}},{\tilde{p}})$ if and only if

11 $$\begin{aligned} \langle {\tilde{m}}({{{\mathcal {S}}}}u), {\tilde{p}}({{{\mathcal {S}}}}u)-h_e {\tilde{p}}({{{\mathcal {S}}}}v) \rangle + \langle {\tilde{m}}({{{\mathcal {S}}}}v), {\tilde{p}}({{{\mathcal {S}}}}v)-h_e^{-1}{\tilde{p}}({{{\mathcal {S}}}}u)\rangle =0 \end{aligned}$$

for every oriented edge orbit ${{{\mathcal {S}}}}e$ with $\phi ({{{\mathcal {S}}}}e)=h_e$. By regarding (11) as a system of linear equations of ${\tilde{m}}$, the corresponding $|E(H)/{{{\mathcal {S}}}}|\times d|V(H)/{{{\mathcal {S}}}}|$-matrix is called the *orbit rigidity matrix*.

In general, for an ${{{\mathcal {S}}}}$-gain graph $(G,\phi )$ and ${\tilde{p}}:V\rightarrow {\mathbb {R}}^d$, we shall define the *orbit rigidity matrix* as an $|E(G)|\times d|V(G)|$-matrix, in which each row corresponds to an edge, each vertex is associated with a *d*-tuple of columns, and the row corresponding to $e=(u,v)\in E(G)$ is written byif *e* is not a loop, and byif *e* is a loop. The orbit rigidity matrix of $(G,\phi ,{\tilde{p}})$ is denoted by $O(G,\phi ,{\tilde{p}})$. From the above discussion, it follows that the dimension of the ${{{\mathcal {S}}}}$-symmetric infinitesimal motions can be computed from the rank of the orbit rigidity matrix of the corresponding quotient gain graph, which is formally stated as follows:

#### **Theorem 5.1**

(Schulze and Whiteley [23]). Let (*H*, *p*) be an $({{{\mathcal {S}}}},\rho )$-symmetric framework with a free action $\rho$. Then,

$$\begin{aligned} \dim L_{{{\mathcal {S}}}}(H, p)=d|V(H)/{{{\mathcal {S}}}}|-{{\mathrm{rank}}}O(H/{{{\mathcal {S}}}},\phi , {\tilde{p}}), \end{aligned}$$

where $(H/{{{\mathcal {S}}}},\phi )$ is the quotient ${{{\mathcal {S}}}}$-gain graph and ${\tilde{p}}$ is a joint-configuration of vertex orbits corresponding to *p*.

### Necessary Condition for Symmetry-Forced Rigidity

Combining some observations given in Sect. 2, we can show a necessary condition for the row independence of orbit rigidity matrices.

#### **Lemma 5.2**

Let $(G,\phi )$ be an ${{{\mathcal {S}}}}$-gain graph with underlying graph $G=(V,E)$, and let $p:V\rightarrow {\mathbb {R}}^d$. If $O(G,\phi ,p)$ is row independent, then

$$\begin{aligned} |F|\le \sum _{F_i\in C(F)}\{d|V(F_i)|-\dim \mathrm{iso}_{\langle F_i\rangle _{\phi ,w}}(p(F_i))\} \end{aligned}$$

for all $F\subseteq E$ and $w\in V(F_i)$, where $p(F_i)=\{gp(v):v\in V(F_i),g\in {{{\mathcal {S}}}}\}$.

#### *Proof*

Let $R_F$ be the linear space spanned by the row vectors associated with *F* in $O(G,\phi ,p)$. Observe that each non-zero entry of the row vector associated with $e\in F$ is in the columns associated with *V*(*F*). This means that $R_F$ is the direct sum of $R_{F'}$ for $F'\in C(F)$, and hence it suffices to check the statement for a connected *F* with $V(F)=V$.

Clearly, $\dim R_F\le d|V|$. Since $|F|\le \dim R_F$, we now show that $\dim R_F^{\bot }\ge \dim \mathrm{iso}_{\langle F\rangle _{\phi ,w}}(p(F))$, where $R_F^{\bot }$ denotes the orthogonal complement of $R_F$.

To see this we first check that a switching operation does not change the rank of the orbit rigidity matrix. Let $\phi '$ be the gain function obtained from $\phi$ by a switching operation at $v_0$ with $g_0\in {{{\mathcal {S}}}}$. We define $p':V\rightarrow {\mathbb {R}}^d$ by

12 $$\begin{aligned} p'(u)= {\left\{ \begin{array}{ll} p(u) &{} \quad \hbox {if } u\ne v_0, \\ g_0p(u) &{}\quad \hbox {if } u=v_0. \end{array}\right. } \end{aligned}$$

Note that $p'(F)=\{gp'(v):v\in V,g\in {{{\mathcal {S}}}}\}=p(F)$. We now show

13 $$\begin{aligned} {{\mathrm{rank}}}O(G,\phi ,p)={{\mathrm{rank}}}O(G,\phi ',p'). \end{aligned}$$

Let us consider a non-loop edge $e=(u,v_0)$ oriented to $v_0$ in *G*. The row corresponding to *e* in $O(G,\phi ',p')$ is written byBy (1), we have $\phi '(e)=\phi (e)g_0^{-1}$. Thus, by using (12), the row of *e* becomesSimilarly, for a non-loop edge $e=(v_0,u)$ oriented from $v_0$ in *G*, the row of *e* becomes exactly the same form as above. By using the same calculation, for a loop *e* incident to $v_0$ in *G*, the row of *e* in $O(G',\phi ',p')$ can be written asBy performing column operations within the *d* columns associated with $v_0$, these are converted toandrespectively, which implies that ${{\mathrm{rank}}}O(G,\phi ,p)={{\mathrm{rank}}}O(G,\phi ',p')$. Therefore, the row independence of the orbit rigidity matrix is invariant under switching operations. Moreover, since $p(F)=p'(F)$, $\dim \mathrm{iso}_{\langle F\rangle _{\phi ,w}}(p(F))=\dim \mathrm{iso}_{\langle F\rangle _{\phi ',w}}(p'(F))$. So it suffices to prove the statement for $O(G,\phi ',p')$.

Let *T* be a spanning tree of *G*. Since we can freely apply switching operations, we may assume that $\phi (e)=\mathrm{id}$ for all $e\in T$. Then, by Lemma 2.3, $\langle F\rangle _{\phi ,w}=\langle \phi (e):e\in F{\setminus } T\rangle$ for a vertex $w\in V(F)$.

Let us take any $m\in \mathrm{iso}_{\langle F\rangle _{\phi ,w}}(p(F))$ and let ${\tilde{m}}:V\rightarrow {\mathbb {R}}^d$ be defined by ${\tilde{m}}(v)=m(p(v))$ for $v\in V$. We show that ${\tilde{m}}$ is in the orthogonal complement of $R_F$. To check it, let us consider any edge $e=(u,v)\in F$ with gain $h=\phi (e)$. Since $m\in \mathrm{iso}(p(F))$, we have

$$\begin{aligned} \langle p(u)-hp(v), m(p(u))-m(hp(v))\rangle =0. \end{aligned}$$

Since *m* is $\langle F\rangle _{\phi ,w}$-symmetric, we also have $m(hp(v))=hm(p(v))$. Therefore, we obtainimplying that ${\tilde{m}}$ is in the orthogonal complement of $R_F$. Consequently, $\dim R_F^{\bot }\ge \dim \mathrm{iso}_{\langle F\rangle _{\phi ,w}}(p(F))$, and hence $|F|\le \dim R_F\le d|V|-\dim \mathrm{iso}_{\langle F\rangle _{\phi ,w}}(p(F))$. $\square$

This, together with Theorem 5.1, directly implies a necessary condition for symmetric frameworks to be symmetry-forced infinitesimally rigid.

Recall that ${{{\mathcal {S}}}}$ is a finite family of orthogonal matrices. Let ${\mathbb {Q}}_{{{\mathcal {S}}}}$ be the field generated by ${\mathbb {Q}}$ and the entries of all the matrices in ${{{\mathcal {S}}}}$. Since ${{{\mathcal {S}}}}$ is finite, almost all numbers in ${\mathbb {R}}$ are transcendental over ${\mathbb {Q}}_{{{\mathcal {S}}}}$. For a given gain graph $(G,\phi )$, a mapping ${\tilde{p}}:V(G)\rightarrow {\mathbb {R}}^d$ is called ${{{\mathcal {S}}}}$-*generic* if the set of coordinates of ${\tilde{p}}(v)$ for all $v\in V(G)$ is algebraically independent over ${\mathbb {Q}}_{{{\mathcal {S}}}}$. Similarly, for a given $({{{\mathcal {S}}}},\rho )$-symmetric graph *H*, an $({{{\mathcal {S}}}},\rho )$-symmetric joint-configuration $p:V(H)\rightarrow {\mathbb {R}}^d$ is called ${{{\mathcal {S}}}}$-*generic* if the corresponding joint-configuration ${\tilde{p}}$ of the vertex orbits is ${{{\mathcal {S}}}}$-generic. An ${{{\mathcal {S}}}}$-symmetric framework is called ${{{\mathcal {S}}}}$-*generic* if the joint configuration is ${{{\mathcal {S}}}}$-generic.

In Sects. 6 and 8 we will check that the condition of Lemma 5.2 is indeed sufficient for generic symmetric frameworks in the plane with cyclic groups and dihedral groups ${{{\mathcal {D}}}}_{2k}$ with odd *k*, respectively.

## Combinatorial Characterization of Generic Rigidity with Cyclic Symmetry

In this section we shall prove a combinatorial characterization of the symmetry-forced rigidity of ${{{\mathcal {S}}}}$-generic symmetric frameworks with cyclic point groups in the plane. The following lemma is a key observation, which is an extension of the one given in [26, 28] for proving Laman’s theorem. The lemma is not limited to cyclic groups.

### **Lemma 6.1**

Let $(G,\phi )$ be an ${{{\mathcal {S}}}}$-gain graph for a point group ${{{\mathcal {S}}}}\subset {{{\mathcal {O}}}}({\mathbb {R}}^2)$. Let $(G',\phi ')$ be an ${{{\mathcal {S}}}}$-gain graph obtained from $(G,\phi )$ by a 0-extension, 1-extension, or loop-1-extension. If there is a mapping $p:V(G)\rightarrow {\mathbb {R}}^2$ such that $O(G,\phi ,p)$ is row independent, then there is a mapping $p':V(G')\rightarrow {\mathbb {R}}^2$ such that $O(G',\phi ',p')$ is row independent.

### *Proof*

If there is a *p* such that $O(G,\phi ,p)$ is row independent, then $O(G,\phi ,q)$ is row independent for all ${{{\mathcal {S}}}}$-generic *q*. Hence, we may assume that *p* is ${{{\mathcal {S}}}}$-generic. We only show the difficult case where $(G',\phi ')$ is constructed from $(G,\phi )$ by a 1-extension (see [8] for the easier case where $(G',\phi ')$ is constructed from $(G,\phi )$ by a 0-extension or a loop-1-extension).

Suppose that $(G',\phi ')$ is obtained from $(G,\phi )$ by a 1-extension, by removing an existing edge *e* and adding a new vertex *v* with three new non-loop edges $e_1, e_2, e_3$ incident to *v*. We may assume that $e_i$ is outgoing from *v*. Let $u_i$ be the other endvertex of $e_i$, and let $g_i=\phi '(e_i)$ and $p_i=p(u_i)$ for $i=1,2,3$. By the definition of 1-extension, we have $\phi (e)=g_1^{-1}g_2$.

### **Claim 6.2**

The three points $g_ip_i \ (i=1,2,3)$ do not lie on a line.

### *Proof*

Since *p* is ${{{\mathcal {S}}}}$-generic, $u_1=u_2=u_3$ should hold if they lie on a line. Then $p_1=p_2=p_3$. By the definition of 1-extensions, $g_i\ne g_j$ if $u_i=u_j$. This implies that $g_1p_1, g_2p_2, g_3p_3$ are three distinct points on a circle. Thus, they do not lie on a line. $\square$

We take $p':V(G')\rightarrow {\mathbb {R}}^2$ such that $p'(w)=p(w)$ for all $w\in V(G)$, and $p'(v)$ is a point on the line through $g_1p_1$ and $g_2p_2$ but is not equal to $g_1p_1$ or $g_2p_2$. $O(G',\phi ',p')$ is described as follows: if $u_1\ne u_2$where the right-bottom block $O(G-e,\phi ,p)$ denotes the orbit rigidity matrix obtained from $O(G,\phi ,p)$ by removing the row of *e*, whereas, if $u_1=u_2$,We consider the case when $u_1\ne u_2$ (the case when $u_1=u_2$ is similar). Since $p'(v)$ lies on the line through $g_1p_1$ and $g_2p_2$, $p'(v)-g_ip(u_i)$ is a scalar multiple of $g_1p_1-g_2p_2$ for $i=1,2$. Hence, by multiplying the rows of $e_1$ and $e_2$ by an appropriate scalar, $O(G',\phi ',p')$ becomesSubtracting the row of $e_1$ from that of $e_2$, we finally getSince $\phi (e)=g_1^{-1}g_2$, the row of $e_2$ is equal to the row of *e* in $O(G,\phi ,p)$. This means that the right-bottom block together with the row of $e_2$ forms $O(G,\phi ,p)$, which is row independent. Thus, the matrix is row independent if and only if the top-left block is row independent. Since $g_ip_i \ (i=1,2,3)$ are not on a line by Claim 6.2, the line through $p'(v)$ and $g_3p_3$ is not parallel to the line through $g_1p_1$ and $g_2p_2$. This implies that the top-left block is row independent, and consequently $O(G',\phi ',p')$ is row independent. $\square$

We are now ready to prove a combinatorial characterization. The same statement was also proved in [12] for rotation groups and in [13] for reflection group by completely different proofs.

### **Theorem 6.3**

Let ${{{\mathcal {C}}}}\subset {{{\mathcal {O}}}}({\mathbb {R}}^2)$ be a cyclic point group, that is, either a group of *k*-fold rotations or a group of a reflection, and let (*H*, *p*) be a generic $({{{\mathcal {C}}}},\rho )$-symmetric framework in the plane with a free action $\rho$. Then (*H*, *p*) is symmetry-forced infinitesimally rigid if and only if the quotient ${{{\mathcal {C}}}}$-gain graph contains a spanning maximum (2, 3)-g-tight subgraph.

### *Proof*

By Theorem 5.1 it suffices to show that for the quotient ${{{\mathcal {C}}}}$-gain graph $(H/{{{\mathcal {C}}}},\phi )$ and any ${{{\mathcal {C}}}}$-generic ${\tilde{p}}:V(H/{{{\mathcal {C}}}})\rightarrow {\mathbb {R}}^2$, $O(H/{{{\mathcal {C}}}},\phi ,{\tilde{p}})$ is row independent if and only if $(H/{{{\mathcal {C}}}},\phi )$ is (2, 3)-g-sparse. Let us simply denote $G=H/{{{\mathcal {C}}}}$.

(“If part”) It suffices to consider the case when *G* is maximum (2, 3)-g-tight. The proof is done by induction on |*V*(*G*)|. For $|V(G)|=1$, *G* consists of single vertex with an unbalanced loop. Then $O(G,\phi ,{\tilde{p}})$ consists of a nonzero row, which implies that $O(G,\phi ,{\tilde{p}})$ is row-independent.

For $|V(G)|>1$, by Theorem 4.4, *G* can be built up from a ${{{\mathcal {C}}}}$-gain graph with one vertex and an unbalanced loop with a sequence of 0-extensions, 1-extensions, and loop-1-extensions. Thus, there is a maximum (2, 3)-g-tight graph $(G',\phi ')$ from which $(G,\phi )$ is constructed by a 0-extension, 1-extension, or loop-1-extension. By induction, there is a $p'$ such that $O(G,\phi ',p')$ is row independent. Thus, Lemma 6.1 implies that there is a *p* such that $O(G,\phi ,p)$ is row independent, which in turn implies that $O(G,\phi ,q)$ is row independent for all ${{{\mathcal {C}}}}$-generic *q*.

(“Only-if part”) The necessity is based on Lemma 5.2. Suppose that $O(G,\phi ,{\tilde{p}})$ is row independent. Recall that we have seen the exact value of $\dim \mathrm{iso}_{{{\mathcal {C}}}}(P)$ for ${{{\mathcal {C}}}}\subset {{{\mathcal {O}}}}({\mathbb {R}}^2)$ and a ${{{\mathcal {C}}}}$-symmetric point set $P\subseteq {\mathbb {R}}^2$ in Example 5.2. Since ${\tilde{p}}$ is ${{{\mathcal {C}}}}$-generic, we have

$$\begin{aligned} \mathrm{iso}_{\langle F\rangle _v}({\tilde{p}}(F))= {\left\{ \begin{array}{ll} 3 &{}\quad \hbox {if }F\hbox { is balanced}, \\ 1 &{}\quad \hbox {otherwise} \end{array}\right. } \end{aligned}$$

for all connected $F\subseteq E(G)$ and $v\in V(F)$, where ${\tilde{p}}(F)=\{g{\tilde{p}}(v):v\in V(F), g\in {{{\mathcal {C}}}}\}$. Therefore, by Lemma 5.2, we have

$$\begin{aligned} |F|\le \sum _{F'\in C(F)}\{2|V(F')|-\mathrm{iso}_{\langle F'\rangle _v}({\tilde{p}}(F'))\} \le 2|V(F)|- {\left\{ \begin{array}{ll} 3 &{}\quad \hbox {if }F\hbox { is balanced}, \\ 1 &{}\quad \hbox {otherwise} \end{array}\right. } \end{aligned}$$

for all $F\subseteq E(G)$. Therefore, $(G,\phi )$ is (2, 3)-g-sparse. $\square$

## Constructive Characterization of Maximum ${{{\mathcal {D}}}}$-Tight Graphs

In the previous sections we gave a constructive characterization of (2, 3)-g-sparse graphs and their realizations as symmetry-forced rigid frameworks in the plane with cyclic point group symmetry. We next move to non-cyclic point groups, that is, dihedral groups of order 2*k* that we denote by ${{{\mathcal {D}}}}_{2k}$ (or simply by ${{{\mathcal {D}}}}$). The corresponding matroid, that we construct in the next subsection, is slightly different from the (2, 3)-g-count matroid, as we need to take into account the fact that the underlying group is not cyclic.

### ${{{\mathcal {D}}}}$-Sparsity

Let $(G,\phi )$ be a ${{{\mathcal {D}}}}$-gain graph with underlying graph $G=(V,E)$. We define a function $f_{{{\mathcal {D}}}}:2^E\rightarrow {\mathbb {Z}}$ by

$$\begin{aligned} f_{{{\mathcal {D}}}}(X)= 2|V(X)|-3+\beta (X) \quad (X\subseteq E), \end{aligned}$$

where

$$\begin{aligned} \beta (X)= {\left\{ \begin{array}{ll} 0 &{}\quad \hbox {if }X\hbox { is balanced}, \\ 2 &{}\quad \hbox {if }X\hbox { is unbalanced and cyclic}, \\ 3 &{}\quad \hbox {otherwise}, \end{array}\right. } \end{aligned}$$

and define a class of sparse graphs determined by $f_{{{\mathcal {D}}}}$ as follows.

#### **Definition 7.1**

Let $(G,\phi )$ be a ${{{\mathcal {D}}}}$-gain graph. An edge set $X\subseteq E(G)$ is called ${{{\mathcal {D}}}}$-*sparse* if $|F|\le f_{{{\mathcal {D}}}}(F)$ for any nonempty $F\subseteq X$, and it is called ${{{\mathcal {D}}}}$-*tight* if it is ${{{\mathcal {D}}}}$-sparse with $|X|=f_{{{\mathcal {D}}}}(X)$.

If *E*(*G*) is ${{{\mathcal {D}}}}$-sparse then also $(G,\phi )$ is said to be ${{{\mathcal {D}}}}$-*sparse*, and $(G,\phi )$ is called *maximum*${{{\mathcal {D}}}}$-*tight* if it is ${{{\mathcal {D}}}}$-sparse with $|E(G)|=2|V(G)|$.

By a simple degree of freedom counting argument based on Example 5.2 and Lemma 5.2, it is not difficult to see that ${{{\mathcal {D}}}}$-sparsity is a necessary condition for orbit rigidity matrices to be row independent in the case of dihedral symmetry (a formal proof will be given in Lemma 8.1). To prove the sufficiency, the first question is whether ${{{\mathcal {D}}}}$-sparsity defines a collection of independent sets of a matroid. This will be proved in this subsection.

We will use the following technical lemmas on properties of ${{{\mathcal {D}}}}$-tight sets.

#### **Lemma 7.1**

Let $(G,\phi )$ be a ${{{\mathcal {D}}}}$-sparse graph with $G=(V,E)$ and $F\subseteq E$ be a ${{{\mathcal {D}}}}$-tight set. Then, the following holds.

(i) If *F* is cyclic, then *F* is connected.
(ii) If *F* is balanced with $|F|> 1$, then *F* has neither parallel edges nor loops and is 2-connected and essentially 3-edge-connected.

#### *Proof*

Since *G* is ${{{\mathcal {D}}}}$-sparse and $\beta$ is monotone nondecreasing, we have $|F|\le \sum _{F'\in C(F)}f_{{{\mathcal {D}}}}(F')\le 2|V(F)|-(3-\beta (F))c$, where *c* denotes the number of connected components in *F*. Hence, if *F* is not connected and $\beta (F)<3$, then $|F|<2|V(F)|-3+\beta (F)$, implying that *F* is not ${{{\mathcal {D}}}}$-tight. Therefore if $\beta (F)<3$ then *F* is connected.

Suppose further that *F* is balanced. Then we have $\beta (X)=0$ for any $X\subseteq F$. This means that $|X|\le f_{2,3}(X)$ for any nonempty $X\subseteq F$, and $|F|=f_{{{\mathcal {D}}}}(F)=2|V(F)|-3=f_{2,3}(F)$. In other words, *F* is independent in the generic 2-rigidity matroid ${{{\mathcal {M}}}}(f_{2,3})$ of *G*[*F*]. It is known that, in the generic 2-rigidity matroid, an independent set *F* with $|F|=f_{2,3}(F)$ and $|F|>1$ has neither parallel edges nor a loop and is 2-connected and essentially 3-edge-connected (see e.g. [7]). $\square$

#### **Lemma 7.2**

Let $(G,\phi )$ be a ${{\mathcal {D}}}$-sparse graph with $G=(V,E)$. Let $X,Y\subseteq E$ be ${{{\mathcal {D}}}}$-tight edge sets with $X\cap Y\ne \emptyset$. Then $X\cup Y$ is ${{{\mathcal {D}}}}$-tight.

#### *Proof*

Without loss of generality, assume $\beta (X)\ge \beta (Y)$.

Let $d=2|V(X\cup Y)|-|X\cup Y|$. Note that $X\cup Y$ is ${{{\mathcal {D}}}}$-tight if one of the following holds: (i) $d=0$, (ii) $d\le 1$ and $X\cup Y$ is cyclic, or (iii) $d\le 3$ and $X\cup Y$ is balanced.

Let $c_0$ be the number of isolated vertices in the graph $(V(X)\cap V(Y), X\cap Y)$ and $c_1$ be the number of connected components in $X\cap Y$. We have $|X|=2|V(X)|-3+\beta (X)$ and $|Y|=2|V(Y)|-3+\beta (Y)$. We also have

14 $$\begin{aligned} |X\cap Y|&\le \sum _{F\in C(X\cap Y)} f_{{{\mathcal {D}}}}(F)= 2|V(X\cap Y)|-3c_1+\sum _{F\in C(X\cap Y)}\beta (F) \nonumber \\&=2|V(X)\cap V(Y)|-2c_0-3c_1+\sum _{F\in C(X\cap Y)}\beta (F) \nonumber \\&\le 2|V(X)\cap V(Y)|-2c_0-3c_1+\beta (Y)c_1 \end{aligned}$$

since $\beta$ is monotone non-decreasing. Therefore,

15 $$\begin{aligned} d&=2|V(X\cup Y)|-|X\cup Y|=2|V(X\cup Y)|-(|X|+|Y|-|X\cap Y|)\nonumber \\&\le 6-\beta (X)-\beta (Y)-2c_0-3c_1+\beta (Y)c_1 \nonumber \\&\le 3-\beta (X)-2c_0-(3-\beta (Y))(c_1-1). \end{aligned}$$

Note that $c_1\ge 1$ by $X\cap Y\ne \emptyset$ and hence $(3-\beta (Y))(c_1-1)\ge 0$.

If $\beta (X)=3$, then (15) implies that $d=0$ and hence $X\cup Y$ is ${{{\mathcal {D}}}}$-tight.

Therefore we assume $\beta (X)<3$. Then *X* and *Y* are connected by Lemma 7.1. We split the proof into two cases depending on the value of $\beta (X)$.

(Case 1) If $\beta (X)=2$, then (15) implies that $d\le 1$. Since $d=0$ implies the ${{{\mathcal {D}}}}$-tightness of $X\cup Y$, let us assume $d=1$ and prove that $X\cup Y$ is cyclic. If $d=1$, then the inequalities of (14) and (15) hold with equalities, and in particular $c_0=0$, $c_1=1$ and

16 $$\begin{aligned} |X\cap Y|=2|V(X\cap Y)|-3+\beta (Y). \end{aligned}$$

By $c_0=0$ and $c_1=1$, the number of connected components in the graph $(V(X)\cap V(Y),X\cap Y)$ is one. If $\beta (Y)=2$, then $X\cap Y$ is unbalanced cyclic by (16) and hence Lemma 2.4(3) implies that $X\cup Y$ is cyclic. If $\beta (Y)=0$, then *Y* is balanced and, again, Lemma 2.4(2) implies that $X\cup Y$ is cyclic. Thus $X\cup Y$ is ${{{\mathcal {D}}}}$-tight.

(Case 2) If $\beta (X)=0$, then $\beta (Y)=0$ and we have $d\le 6-2c_0-3c_1$ by (15). By $c_1\ge 1$, we have three possible pairs $(c_0,c_1)=(0,1), (1,1), (0,2)$. If $(c_0,c_1)=(0,1)$, then $d\le 3$ and Lemma 2.4 implies that $X\cup Y$ is balanced. Thus, $X\cup Y$ is a balanced ${{{\mathcal {D}}}}$-tight set. If $(c_0,c_1)=(1,1)$ or $(c_0,c_1)=(0,2)$, then $d\le 1$ and Lemma 2.5 implies that $X\cup Y$ is cyclic. Thus, $X\cup Y$ is a cyclic ${{{\mathcal {D}}}}$-tight set.

This completes the proof. $\square$

#### **Lemma 7.3**

Let $(G,\phi )$ be a ${{{\mathcal {D}}}}$-gain graph with $G=(V,E)$ and *X* and *Y* be ${{{\mathcal {D}}}}$-tight sets with $X\subseteq Y\subseteq E$. For $e\in E{\setminus } Y$, if $f_{{{\mathcal {D}}}}(X)=f_{{{\mathcal {D}}}}(X+e)$, then $f_{{{\mathcal {D}}}}(Y)=f_{{{\mathcal {D}}}}(Y+e)$.

#### *Proof*

Since $f_{{{\mathcal {D}}}}(X)=f_{{{\mathcal {D}}}}(X+e)$, the endvertices of *e* are contained in *V*(*X*), implying $V(Y+e)=V(Y)$. If *X* or *Y* is not cyclic, then we have $\beta (Y)=\beta (Y+e)=3$, meaning that $f_{{{\mathcal {D}}}}(Y)=f_{{{\mathcal {D}}}}(Y+e)$.

We hence assume that *X* and *Y* are cyclic, and they are connected by Lemma 7.1. Take a spanning tree *T* in *G*[*Y*] such that $X\cap T$ is a spanning tree of *G*[*X*]. By Proposition 2.2, there is an equivalent gain function $\phi '$ to $\phi$ such that $\phi '(f)=\mathrm{id}$ for $f\in T$. By Lemma 2.3, there is a cyclic subgroup ${{{\mathcal {C}}}}$ of ${{{\mathcal {D}}}}$ such that $\phi '(f)\in {{{\mathcal {C}}}}$ for every $f\in Y$, where ${{{\mathcal {C}}}}$ is the trivial group if *Y* is balanced. Since $f_{{{\mathcal {D}}}}(X)=f_{{{\mathcal {D}}}}(X+e)$ and $X\subseteq Y$, we have $\phi '(e)\in \bar{{{\mathcal {C}}}}$, and hence $f_{{{\mathcal {D}}}}(Y)=f_{{{\mathcal {D}}}}(Y+e)$ holds. $\square$

We are ready to prove that the family of ${{{\mathcal {D}}}}$-sparse edge subsets is a family of independent sets of a matroid on ground-set *E*. We shall also characterize the rank function of this matroid.

#### **Theorem 7.4**

Let $(G,\phi )$ be a ${{{\mathcal {D}}}}$-gain graph with $G=(V,E)$ and ${{{\mathcal {I}}}}$ be the family of all ${{{\mathcal {D}}}}$-sparse edge subsets in *E*. Then ${{{\mathcal {M}}}}_{{{\mathcal {D}}}}(G,\phi )=(E,{{{\mathcal {I}}}})$ is a matroid on ground-set *E*. The rank of a set $E'\subseteq E$ in ${{{\mathcal {M}}}}_{{{\mathcal {D}}}}(G,\phi )$ is equal to

$$\begin{aligned} \min \Biggl \{ \sum _{i=1}^t f_{{{\mathcal {D}}}}(E_i'):\{E_1',\ldots , E_t'\}\ \hbox { is a partition of } E' \Biggr \}. \end{aligned}$$

#### *Proof*

For a partition ${{{\mathcal {P}}}}=\{E_1',\ldots , E_t'\}$ of $E'\subseteq E$, we denote $\mathrm{val}({{{\mathcal {P}}}})=\sum _{i=1}^tf_{{{\mathcal {D}}}}(E_t')$. We shall check the following independence axiom of matroids: (I1) $\emptyset \in {{{\mathcal {I}}}}$; (I2) for any $X, Y\subseteq E$ with $X\subseteq Y$, $Y\in {{{\mathcal {I}}}}$ implies $X\in {{{\mathcal {I}}}}$; (I3) for any $E'\subseteq E$, maximal subsets of $E'$ belonging to ${{{\mathcal {I}}}}$ have the same cardinality.

${{{\mathcal {I}}}}$ obviously satisfies (I1) and (I2). To see (I3), let $E'\subseteq E$ and let $F\subseteq E'$ be a maximal subset of $E'$ in ${\mathcal {I}}$. Since $F\in {\mathcal {I}}$ we have $|F|\le \mathrm{val}({\mathcal {P}})$ for all partitions ${\mathcal {P}}$ of $E'$. We shall prove that there is a partition ${\mathcal {P}}$ of $E'$ with $|F|=\mathrm{val}({\mathcal {P}})$, from which (I3) follows.

Let $J=(V,F)$ denote the subgraph with the edge set *F*. Consider the family $\{F_{1},F_{2},\ldots ,F_{t}\}$ of all maximal ${{{\mathcal {D}}}}$-tight sets in *J*. Since each edge $f\in F$ forms a ${{{\mathcal {D}}}}$-tight set, $\bigcup _{i=1}^{t}F_i=F$ holds. Since $F_{i}\cap F_{j}=\emptyset$ for every pair $1\le i<j\le t$ by Lemma 7.2 and the maximality, ${\mathcal {P}}_F=\{F_{1},F_{2},\ldots ,F_{t}\}$ is a partition of *F* and $|F|=\mathrm{val}({\mathcal {P}}_F)$ follows.

Based on ${{{\mathcal {P}}}}_F$, we construct a partition ${\mathcal {P}}$ of $E'$ with $\mathrm{val}({\mathcal {P}})=\mathrm{val}({\mathcal {P}}_F)=|F|$. Consider an edge $(u,v)=e\in E'-F$. Since *F* is a maximal subset of $E'$ in ${\mathcal {I}}$ we have $F+e\not \in {\mathcal {I}}$. Hence there must be a ${{\mathcal {D}}}$-tight set $X_e$ in *J* with $u,v\in V(X_e)$ and $X_e+e\not \in {\mathcal {I}}$. $X_e\subseteq F_{i}$ for some $1\le i\le t$. Choose such an $F_i$ for every $e\in E'-F$ and define $E_i=F_i\cup \{e:F_i \hbox { was chosen for }e\}$. Clearly ${\mathcal {P}}=\{E_{1},E_{2},\ldots ,E_{t}\}$ is a partition of $E'$. By Lemma 7.3, $f_{{\mathcal {D}}}(F_i)=f_{{\mathcal {D}}}(E_i)$ for every $1\le i\le t$ and hence $\mathrm{val}({\mathcal {P}})=\mathrm{val}({\mathcal {P}}_F)=|F|$. $\square$

The matroid which was introduced and denoted by ${{{\mathcal {M}}}}_{{{\mathcal {D}}}}(G,\phi )$ in Theorem 7.4 is called the ${{{\mathcal {D}}}}$-*sparsity matroid* of $(G,\phi )$.

### Constructive Characterization of Maximum ${{{\mathcal {D}}}}$-Tight Graphs

We now present a constructive characterization of maximum ${{{\mathcal {D}}}}$-tight graphs. Notice that the average vertex degree in a maximum ${{{\mathcal {D}}}}$-tight graph $(G,\phi )$ is four, which means that *G* has a vertex of degree at most 3 if and only if *G* is not 4-regular. Thus we shall take special care with 4-regular ${{{\mathcal {D}}}}$-sparse graphs.

#### 0-Extension, 1-Extension, and Loop-1-Extension

Before looking at 4-regular graphs and vertices of degree four, we consider the 0-extension, 1-extension, and loop-1-extension operations. Recall that the corresponding inverse operations are called reductions. A reduction is *admissible* if the resulting graph is ${{{\mathcal {D}}}}$-sparse.

##### **Lemma 7.5**

Let $(G,\phi )$ be a ${{{\mathcal {D}}}}$-sparse graph with $G=(V,E)$. Applying a 0-extension, 1-extension or loop-1-extension to $(G,\phi )$ results in a ${{{\mathcal {D}}}}$-sparse graph with $|V|+1$ vertices and $|E|+2$ edges.

Conversely, for any vertex *v* of degree 2 or 3, the 0-reduction, loop-1-reduction, or some of the 1-reductions at *v* are admissible if $|V|\ge 2$.

##### *Proof*

The proof of the first claim is exactly the same as the proof of Lemma 4.1 (indeed, we just need to change $2\alpha (F)$ with $\beta (F)$ in the proof of Lemma 4.1).

To see that some reduction is admissible at a vertex *v* of degree three, we just need to observe that each circuit of ${{{\mathcal {M}}}}(g_{2,3})$ appearing in the proof of Claim 4.3 is also a circuit in ${{{\mathcal {M}}}}_{{{\mathcal {D}}}}(G,\phi )$. We can thus apply exactly the same proof as in Lemma 4.2 to conclude that some reduction is admissible at *v*. $\square$

#### 2-Extension and Loop-2-Extension

Besides 0-extensions, 1-extensions and loop-1-extensions, we shall introduce 2-*extensions* and *loop*-2-*extensions* for constructing 4-regular ${{{\mathcal {D}}}}$-sparse graphs.

**Fig. 4:**
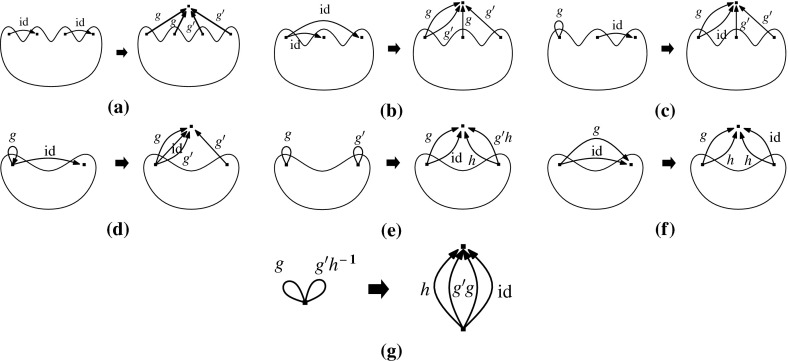
2-Extensions

In a 2-*extension*, we take two existing edges $e=(v_1,v_2)$ and $f=(v_3,v_4)$ and pinch them by inserting a new vertex *v*. More precisely, a 2-extension removes *e* and *f*, inserts a new vertex *v* with four new edges, $e_i$ from $v_i$ to *v* for each $i=1,\ldots , 4$. The gain function $\phi$ is extended on $E\cup \{e_1,\ldots , e_4\}$ so that $\phi (e_1)\cdot \phi (e_2)^{-1}=\phi (e)$, $\phi (e_3)\cdot \phi (e_4)^{-1}=\phi (f)$, and it is locally ${{{\mathcal {D}}}}$-sparse, i.e., $\{e_1,\ldots , e_4\}$ is ${{{\mathcal {D}}}}$-sparse. Depending on the multiplicity of the $v_i$’s we have seven cases as shown in Fig. 4.

**Fig. 5:**
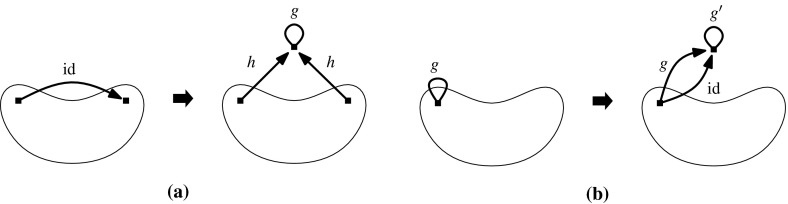
Loop-2-extensions

In a *loop*-2-*extensions*, we remove an existing edge $e=(v_1,v_2)$, insert a new vertex *v*, a new loop *l* at *v* and two new edges, $e_i$ from $v_i$ to *v* for each $i=1,2$. $\phi$ is extended on $E\cup \{e_1,e_2,l\}$ so that $\phi (e_1)\cdot \phi (e_2)^{-1}=\phi (e)$, $\phi (l)\ne \mathrm{id}$, and it is locally ${{{\mathcal {D}}}}$-sparse. Depending on whether *e* is a loop or not, we have two cases as shown in Fig. 5.

The following lemma shows that these operations preserve ${{{\mathcal {D}}}}$-sparsity.

##### **Lemma 7.6**

Let $(G,\phi )$ be a ${{{\mathcal {D}}}}$-sparse graph. Then, any ${{{\mathcal {D}}}}$-gain graph $(G',\phi ')$ obtained from *G* by a 2-extension or a loop-2-extension is ${{{\mathcal {D}}}}$-sparse.

##### *Proof*

Suppose that $(G',\phi ')$ is obtained by a 2-extension. Let us denote the removed edges by *e* and *f* and the new edges by $e_1,\ldots , e_4$ as above. Suppose that there is $F\subseteq E(G')$ that violates the ${{{\mathcal {D}}}}$-sparsity condition. Let $F'=F{\setminus } \{e_1,\ldots , e_4\}$. Since $\{e_1,\ldots , e_4\}$ satisfies the ${{{\mathcal {D}}}}$-sparsity condition, $F'\ne \emptyset$. Let us add *e* to $F'$ if $\{e_1,e_2\}\subseteq F$ and add *f* to $F'$ if $\{e_3,e_4\}\subseteq F$. Observe that $|F'|\ge |F|-2$, $|V(F)|\ge |V(F')|+1$ and $\beta (F)\ge \beta (F')$. Since $|F|>f_{{{\mathcal {D}}}}(F)$, we obtain $|F'|\ge |F|-2>f_{{{\mathcal {D}}}}(F)-2=2|V(F)|-3+\beta (F)-2\ge 2|V(F')|-3+\beta (F')=f_{{{\mathcal {D}}}}(F')$. This contradicts the ${{{\mathcal {D}}}}$-sparsity of *G* since $\emptyset \ne F'\subseteq E(G)$. Therefore $(G',\phi ')$ is ${{{\mathcal {D}}}}$-sparse.

In the same manner, it can be easily checked that a loop-2-extension also preserves ${{{\mathcal {D}}}}$-sparsity. $\square$

We shall define the inverse moves of these operations. Recall that, for a vertex *v* and two incoming non-loop edges $e_1=(u,v)$ and $e_2=(w,v)$, we denote by $e_1\cdot e_2^{-1}$ a new edge from *u* to *w* with gain $\phi (e_1)\cdot \phi (e_2)^{-1}$.

Let *v* be a vertex of degree four, not incident to a loop, and $e_i=(v_i,v)$ for $i=1,\ldots , 4$ be the edges incident to *v*, assuming that all of them are oriented to *v*. The 2-*reduction* (at *v*) deletes *v* and adds one of $\{e_1\cdot e_2^{-1}, e_3\cdot e_4^{-1}\}$, $\{e_1\cdot e_3^{-1}, e_2\cdot e_4^{-1}\}$ and $\{e_1\cdot e_4^{-1}, e_2\cdot e_3^{-1}\}$. We sometimes refer to a specific one: the 2-*reduction atvthrough*$(e_i, e_j)$*and*$(e_k, e_l)$ deletes *v* and adds $\{e_i\cdot e_j^{-1}, e_k\cdot e_l^{-1}\}$.

Let *v* be a vertex of degree four, incident to a loop *l*, and $e_i=(v_i,v)$ for $i=1,2$ be the non-loop edges incident to *v*, assuming that all of them are oriented to *v*. The *loop*-2-*reduction* (at *v*) deletes *v* and adds $e_1\cdot e_2^{-1}$.

A 2-reduction or a loop-2-reduction is said to be *admissible* if the resulting graph is ${{{\mathcal {D}}}}$-sparse.

#### Base Graphs

Our main theorem asserts that these operations are sufficient to construct all 4-regular ${{{\mathcal {D}}}}$-sparse graphs from certain classes of ${{{\mathcal {D}}}}$-sparse graphs. Here, the classes can be categorized into three groups: the first group includes special small graphs as in the conventional constructive characterizations, the second group is a class of graphs, which are obtained from cycles by duplicating each edge, and the third one consists of *near-cyclic* 4-*regular graphs*.

**Fig. 6:**
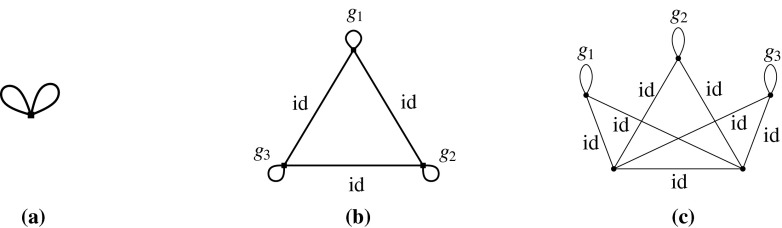
Special graphs: **a** A trivial graph. **b** A fancy triangle. **c** A fancy hat

The first group consists of three types of special ${{{\mathcal {D}}}}$-tight graphs, called *trivial graphs*, *fancy triangles*, and *fancy hats*. A *trivial graph* is a ${{{\mathcal {D}}}}$-sparse graph with a single vertex and with two loops as shown in Fig. 6a. The gain function is assigned so that the gains of two loops generate a non-cyclic group.

A *fancy triangle* is a ${{{\mathcal {D}}}}$-gain graph whose underlying graph is obtained from a triangle by adding a loop to each vertex, as shown in Fig. 6b. The gain function is assigned so that it is ${{{\mathcal {D}}}}$-sparse and the triangle is balanced.

A *hat* is a graph obtained from $K_{2,3}$ by adding an edge to the class of cardinality two, and the *fancy hat* is a ${{{\mathcal {D}}}}$-gain graph obtained from the hat by adding a loop to each degree two vertex, as shown in Fig. 6c. The gain function is assigned so that it is ${{{\mathcal {D}}}}$-sparse and the hat is balanced.

**Fig. 7:**
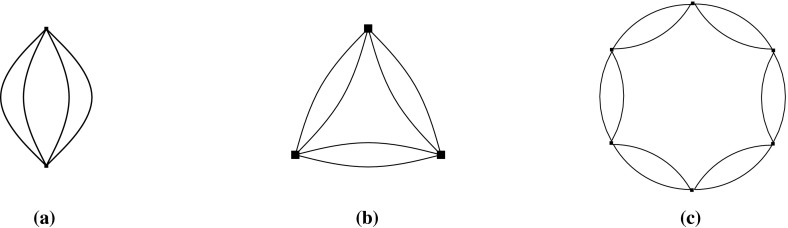
Double cycles: **a** $$C_2^2$$. **b** $$C_3^2$$. **c** $$C_6^2$$

The second group consists of ${{{\mathcal {D}}}}$-sparse graphs whose underlying graphs are double cycles, where, for $n\ge 2$, the *double cycle*$C_n^2$ is defined as the graph obtained from the cycle on *n* vertices by replacing each edge by two parallel edges as shown in Fig. 7. As we will see later, key properties of this group depend on the parity of *k* of the underlying dihedral group ${{{\mathcal {D}}}}_{2k}$.

The third group consists of *near-cyclic* graphs, which, intuitively speaking, are the ${{{\mathcal {D}}}}$-tight graphs closest to (2, 3)-g-tight graphs. More precisely, we say that a ${{{\mathcal {D}}}}$-sparse graph $(G,\phi )$ is *near-cyclic* if there is an edge *e* such that $(G-e,\phi )$ is cyclic.

The following lemma shows how to construct near-cyclic graphs.

##### **Lemma 7.7**

Let $(G,\phi )$ be a (2, 3)-g-sparse ${{{\mathcal {D}}}}$-gain graph with $G=(V,E)$, and suppose that there is a cyclic subgroup ${{{\mathcal {C}}}}$ of ${{{\mathcal {D}}}}$ such that $\phi (e)\in {{{\mathcal {C}}}}$ for all $e\in E$. If we add a new edge *e* having a gain in ${{{\mathcal {D}}}}{\setminus } \bar{{{\mathcal {C}}}}$, then $(G+e,\phi )$ is ${{{\mathcal {D}}}}$-sparse.

##### *Proof*

Suppose that $(G+e,\phi )$ is not ${{{\mathcal {D}}}}$-sparse. Then there is a subset $F\subseteq E$ such that $|F+e|>f_{{{\mathcal {D}}}}(F+e)$. Since *F* is cyclic, either (i) $\beta (F)=0$ or (ii) $\beta (F)=2$.

By Lemma 7.1(i), *F* is connected, and clearly the endvertices of *e* are contained in *V*(*F*). Moreover, every cycle in $F+e$ that passes through *e* has a gain not contained in $\bar{{{\mathcal {C}}}}$, as the gain of *e* is not in $\bar{{{\mathcal {C}}}}$. Thus, $F+e$ contains a cycle whose gain is not contained in $\bar{{{\mathcal {C}}}}$. This means that (i) $\beta (F)=0$ implies $\beta (F+e)=1$, and (ii) $\beta (F)=2$ implies $\beta (F+e)=3$. In each case, we obtain $\beta (F+e)-\beta (F)\ge 1$. Therefore, $|F|=|F+e|-1>f_{{{\mathcal {D}}}}(F+e)-1 \ge f_{{{\mathcal {D}}}}(F)$, and this contradicts the ${{{\mathcal {D}}}}$-sparsity of $(G,\phi )$ as $\emptyset \ne F\subseteq E$. $\square$

#### Constructive Characterizations

We are ready to state our constructive characterization of 4-regular ${{{\mathcal {D}}}}$-sparse graphs. We say that a 4-regular ${{{\mathcal {D}}}}$-sparse graph is a *base graph* if it is a trivial graph, a fancy triangle, a fancy hat, or a near-cyclic graph.

##### **Theorem 7.8**

Let $(G,\phi )$ be a ${{{\mathcal {D}}}}$-gain graph. Then, $(G,\phi )$ is 4-regular and ${{{\mathcal {D}}}}$-sparse if and only if it can be built up from a disjoint union of base graphs and ${{{\mathcal {D}}}}$-sparse double cycles by a sequence of 2-extension and loop-2-extension operations.

We have proved that these operations preserve ${{{\mathcal {D}}}}$-sparsity in Lemma 7.6. The proof of the converse direction will be given in Sect. 9, where we will show that a 2-reduction or a loop-2-reduction is admissible at some vertex if the graph is neither a base graph nor a double cycle.

Combining Theorem 7.8 and Lemma 7.5, we obtain the following:

##### **Theorem 7.9**

Let $(G,\phi )$ be a ${{{\mathcal {D}}}}_{2k}$-gain graph with $k\ge 2$. Then, $(G,\phi )$ is maximum ${{{\mathcal {D}}}}_{2k}$-tight if and only if it can be built up from a disjoint union of base graphs and ${{{\mathcal {D}}}}_{2k}$-sparse double cycles by a sequence of 0-extension, 1-extension, loop-1-extension, 2-extension and loop-2-extension operations.

The theorems can be strengthened if *k* is odd, in which case every ${{{\mathcal {D}}}}_{2k}$-sparse double cycle can be reduced to a trivial graph. To see this, let us prove the following technical lemma.

##### **Lemma 7.10**

Let ${{{\mathcal {D}}}}_{2k}$ be a dihedral group with odd $k\ge 3$. Let $g_1,g_2,g_3,g_4$ be elements of ${{{\mathcal {D}}}}_{2k}$ such that

- $g_1, g_2$ and $g_3$ are distinct non-identity elements,
- $\{g_1,g_2,g_3\}$ generates a non-cyclic group, and
- $$g_4=\mathrm{id}$$.

Then, at least one of $\{g_1g_2^{-1}, g_3g_4^{-1}\}$, $\{g_1g_3^{-1}, g_2g_4^{-1}\}$ and $\{g_1g_4^{-1}, g_2g_3^{-1}\}$ generates a non-cyclic group.

##### *Proof*

Since $\{g_1,g_2,g_3\}$ generates a non-cyclic group, we may assume that $g_1$ is a reflection *r* along a line. Suppose that $\{g_1,g_2g_3^{-1}\}$ is cyclic. Then, $g_2g_3^{-1}=\mathrm{id}$ or $g_2g_3^{-1}=r$. Since $g_2\ne g_3$, we have $g_2=rg_3$.

If $g_3$ is also a reflection $r'$, which is different from *r*, then consider $\{g_1g_2^{-1},g_3\}=\{rr'r^{-1}, r'\}$. Clearly $rr'r^{-1}\ne \mathrm{id}$. If $rr'r^{-1}=r'$ or equivalently $(rr')^{2}=\mathrm{id}$ then *k* has to be even which is a contradiction. Thus $\{g_1g_2^{-1},g_3\}$ generates a non-cyclic group.

If $g_3$ denotes a rotation *C*, then consider $\{g_1g_3^{-1},g_2\}=\{rC^{-1}, rC\}$. Since $rC^{-1}$ and *rC* are non-identity and reflections, if they generate a cyclic group, then $rC^{-1}=rC$, implying $C^{2}=\mathrm{id}$. This contradicts the parity of *k*. $\square$

##### **Lemma 7.11**

Let ${{{\mathcal {D}}}}_{2k}$ be a dihedral group with odd $k\ge 3$, and $(G,\phi )$ be a ${{{\mathcal {D}}}}_{2k}$-sparse double cycle $C_n^2$ with $n\ge 2$. Then, a 2-reduction is admissible at some vertex.

##### *Proof*

Let *v* be a vertex, and we denote the edges incident to *v* by $e_i$ for $i=1,\ldots , 4$. Without loss of generality, we assume that all of $e_i$ are oriented to *v*, $e_1$ and $e_2$ are parallel, and $e_3$ and $e_4$ are parallel.

**Fig. 8:**
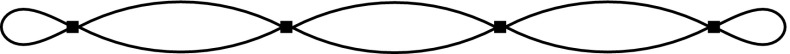
$G'$ in the proof of Lemma 7.11

We first perform the 2-reduction at *v* through $(e_1, e_2)$ and $(e_3,e_4)$. Then, the resulting graph $(G',\phi ')$ is, as shown in Fig. 8, a path of parallel edges with loops at its endvertices. Using the fact that each 2-cycle is unbalanced in *G*, it is easy to check that $|F|\le 2|V(F)|-3$ for any balanced $F\subseteq E(G')$ and $|F|\le 2|V(F)|-1$ for any proper subset $F\subset E(G')$. Thus, $(G',\phi ')$ is ${{{\mathcal {D}}}}_{2k}$-sparse if $E(G')$ is not cyclic.

Suppose that $E(G')$ is cyclic. Then, by Lemma 2.3, we may assume that there is a cyclic subgroup ${{{\mathcal {C}}}}$ of ${{{\mathcal {D}}}}_{2k}$ such that all gains of $E(G')$ are contained in ${{{\mathcal {C}}}}$. Let $a=\phi (e_1\cdot e_2^{-1})$ and $a'=\phi (e_3\cdot e_4^{-1})$. Since any 2-cycle of *G* is unbalanced, *a* and $a'$ are non-identity. Moreover, $\phi (e_1)\cdot \phi (e_2)^{-1}=a\in {{{\mathcal {C}}}}$ and $\phi (e_3)\cdot \phi (e_4)^{-1}=a'\in {{{\mathcal {C}}}}$. Hence, by using some elements $b_1,b_2\in {{{\mathcal {D}}}}_{2k}$, we can express $\phi (e_i)$ by

$$\begin{aligned} \phi (e_1)=ab_1, \quad \phi (e_2)=b_1,\quad \phi (e_3)=a'b_2,\quad \phi (e_4)=b_2. \end{aligned}$$

Let us perform the switching operation at *v* with $b_2$. Then we have

17 $$\begin{aligned} \phi (e_1)=ab,\quad \phi (e_2)=b,\quad \phi (e_3)=a',\quad \phi (e_4)=\mathrm{id}, \end{aligned}$$

where $b=b_1b_2^{-1}$. Recall that $\phi (e)\in {{{\mathcal {C}}}}$ for all $e\in E(G){\setminus } \{e_1,e_2\}$. Since $(G,\phi )$ is maximum ${{{\mathcal {D}}}}_{2k}$-tight, we must have $b\notin \bar{{{\mathcal {C}}}}$.

We now consider the remaining two possible 2-reductions at *v*. In each reduction, the resulting underlying graph is $C_{n-1}^2$, and it can be easily checked that the 2-reduction is admissible if one of the resulting ${{{\mathcal {D}}}}_{2k}$-gain graphs $(G_1,\phi _1)$ and $(G_2,\phi _2)$ is not cyclic.

To see that $(G_1,\phi _1)$ or $(G_2,\phi _2)$ is not cyclic, let $g_i=\phi (e_i)$ for $i=1,\ldots , 4$. Observe that $\{g_1,\ldots , g_4\}$ satisfies the condition of Lemma 7.10. Since $\{g_1\cdot g_2^{-1}, g_3\cdot g_4^{-1}\}$ generates a cyclic group, this implies, by Lemma 7.10, that $\{g_1\cdot g_3^{-1}, g_2\cdot g_4^{-1}\}$ or $\{g_1\cdot g_4^{-1}, g_2\cdot g_3^{-1}\}$ is not cyclic, implying that $(G_1,\phi _1)$ or $(G_2,\phi _2)$ is not cyclic. $\square$

Combining Theorem 7.9 and Lemma 7.11, we obtain the following constructive characterization.

##### **Theorem 7.12**

Let ${{{\mathcal {D}}}}_{2k}$ be a dihedral group with odd $k\ge 3$. Then a ${{{\mathcal {D}}}}_{2k}$-gain graph $(G,\phi )$ is maximum ${{{\mathcal {D}}}}_{2k}$-tight if and only if it can be built up from a disjoint union of base graphs by a sequence of 0-extension, 1-extension, loop-1-extension, 2-extension and loop-2-extension operations.

**Fig. 9:**
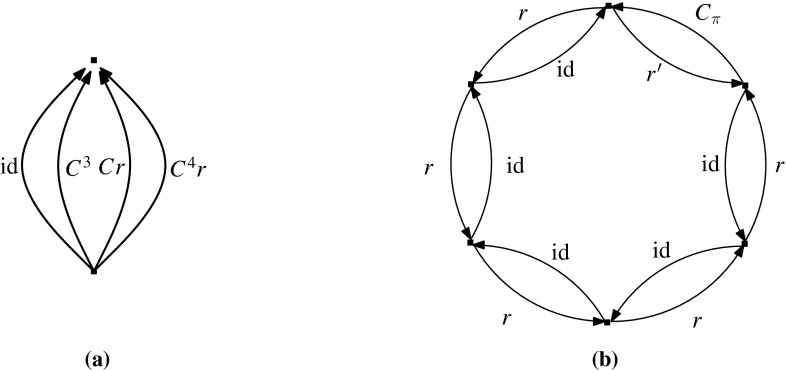
Double cycles without admissible 2-reductions. **a** A ${{{\mathcal {D}}}}_6$-sparse $C_2^2$, where *C* denotes a six-fold rotation and *r* denotes a reflection. **b** A ${{{\mathcal {D}}}}_2$-sparse $C_6^2$, where $C_{\pi }$ denotes a two-fold rotation and *r* and $r'$ denote distinct reflections

Lemma 7.11 does not hold for dihedral groups ${{{\mathcal {D}}}}_{2k}$ with even *k*. See Fig. 9 for examples. In the next section we will see how the combinatorial properties given in the preceeding two lemmas lead to substantial differences between the rigidity properties of frameworks with odd or even *k*.

## Combinatorial Characterization of Generic Rigidity with Dihedral Symmetry

In this section we discuss our combinatorial characterization of symmetry-forced infinitesimal rigidity with dihedral symmetry. We begin with a necessary condition based on Lemma 5.2.

### **Lemma 8.1**

Let ${{{\mathcal {D}}}}_{2k}$ be a dihedral group with $k\ge 2$, and (*H*, *p*) be a generic $({{{\mathcal {D}}}}_{2k},\rho )$-symmetric framework with a free action $\rho$. If (*H*, *p*) is symmetry-forced infinitesimally rigid, then the quotient gain graph contains a spanning maximum ${{{\mathcal {D}}}}_{2k}$-tight subgraph.

### *Proof*

Let $(H/{{{\mathcal {D}}}}_{2k},\phi )$ be the quotient gain graph of *H* and ${\tilde{p}}$ be a joint configuration of the vertex orbits $V(H/{{{\mathcal {D}}}}_{2k})$ corresponding to *p*. By Theorem 5.1, it suffices to prove that if $O(H/{{{\mathcal {D}}}}_{2k},\phi ,{\tilde{p}})$ is row independent, then $(H/{{{\mathcal {D}}}}_{2k},\phi )$ is ${{{\mathcal {D}}}}_{2k}$-sparse.

Since ${\tilde{p}}$ is generic, according to the exact value given in Example 5.2, we have

$$\begin{aligned} \mathrm{iso}_{\langle F\rangle _{\phi ,u}}({\tilde{p}}(F))= {\left\{ \begin{array}{ll} 3 &{}\quad \hbox {if }F\hbox { is balanced}, \\ 1 &{}\quad \hbox {if }F\hbox { is unbalanced and cyclic}, \\ 0 &{}\quad \hbox {otherwise} \end{array}\right. } \end{aligned}$$

for any connected $F\subseteq E(H/{{{\mathcal {D}}}}_{2k})$ and $u\in V(F)$, where ${\tilde{p}}(F)=\{g{\tilde{p}}(v):v\in V(F),g \in {{{\mathcal {D}}}}_{2k}\}$. By this and Lemma 5.2, we have that $|F|\le f_{{{{\mathcal {D}}}}_{2k}}(F)$ for any $F\subseteq E(H/{{{\mathcal {D}}}}_{2k})$. In other words, $(H/{{{\mathcal {D}}}}_{2k},\phi )$ is ${{{\mathcal {D}}}}_{2k}$-sparse. $\square$

In Sect. 8.1 we shall prove that ${{{\mathcal {D}}}}_{2k}$-sparsity is also sufficient for row independence when $k\ge 3$ is odd. On the other hand, in Sect. 8.2 we give a family of examples showing that this implication does not always hold when *k* is even.

### Combinatorial Characterization of Symmetry-Forced Rigidity with ${{{\mathcal {D}}}}_{2k}$-Symmetry for Odd *k*

Our goal of this subsection is to prove the following characterization of symmetry-forced infinitesimal rigidity.

#### **Theorem 8.2**

Let ${{{\mathcal {D}}}}_{2k}$ be a dihedral group with odd $k\ge 3$, and (*H*, *p*) be a generic $({{{\mathcal {D}}}}_{2k},\rho )$-symmetric framework with a free action $\rho$. Then (*H*, *p*) is symmetry-forced infinitesimally rigid if and only if the quotient gain graph contains a spanning maximum ${{{\mathcal {D}}}}_{2k}$-tight subgraph.

Necessity follows from Lemma 8.1. Therefore, by Theorem 5.1, it suffices to prove that, for a maximum ${{{\mathcal {D}}}}_{2k}$-tight graph $(G,\phi )$, there is a mapping $p:V(G)\rightarrow {\mathbb {R}}^2$ such that $O(G,\phi ,p)$ is row independent. The proof of this claim is based on the constructive characterization of maximum ${{{\mathcal {D}}}}_{2k}$-tight graphs formulated in Sect. 7.

By Theorem 7.12, $(G,\phi )$ can be constructed from a disjoint union of base graphs by 0-extension, 1-extension, loop-1-extension, 2-extension, and loop-2-extension operations. Therefore, what we have to prove is that (i) the orbit rigidity matrix of each base graph is row independent and (ii) each extension preserves the row independence of the orbit rigidity matrix by extending *p* appropriately. (i) will be solved in Lemma 8.4 whereas (ii) will be solved in Lemmas 8.5 and 8.7. Note that there is no parity condition for *k* in these lemmas.

In the rest of this section, we identify ${{{\mathcal {D}}}}_{2k}$ with the symmetry group of a regular *k*-gon, which consists of *k*-fold rotations around the origin and reflections along (fixed) lines. For a line *L* through the origin, we denote by $L^{\bot }$ the orthogonal complement of *L*, that is, the line orthogonal to *L* and through the origin. We first note an elementary fact from geometry.

#### **Lemma 8.3**

Let $$g\in {{{\mathcal {O}}}}({\mathbb {R}}^2)$$.

- If *g* is the reflection along a line *L*, then $(I_2-g)p\in L^{\bot }{\setminus } \{0\}$ for any $p\in {\mathbb {R}}^2{\setminus } L$.
- If *g* is a rotation, then $(2I_2-g-g^{-1})p\in \mathrm{span}\{p\}{\setminus } \{0\}$ for any $p\in {\mathbb {R}}^2{\setminus } \{0\}$.

#### **Lemma 8.4**

Let $(G,\phi )$ be a base graph. Then, there is a mapping $p:V(G)\rightarrow {\mathbb {R}}^2$ such that $O(G,\phi ,p)$ is row independent.

#### *Proof*

(Case 1) Suppose that $(G,\phi )$ is a trivial graph. Let *v* be the vertex. Take $p:V(G)\rightarrow {\mathbb {R}}^2$ such that *p*(*v*) does not lie on reflection lines *L* in ${{{\mathcal {D}}}}_{2k}$ and their orthogonal complements $L^{\bot }$. Then, $O(G,\phi ,p)$ consists of two row vectors, which are linearly independent by Lemma 8.3.

(Case 2) Suppose that $(G,\phi )$ is a fancy triangle or a fancy hat. Since these two gain graphs are small, one can check the statement by choosing *p* randomly and computing the rank of $O(G,\phi ,p)$ (see [8] for finding such *p* deterministically).

(Case 3) Suppose that $(G,\phi )$ is near-cyclic. Then there is an edge *e* such that $G-e$ is cyclic. Let ${{{\mathcal {C}}}}=\langle E-e\rangle _v$ for a vertex $v\in V(G)$, and denote $g_e=\phi (e)$. We may assume that the labels of the edges in $E-e$ are all contained in ${{{\mathcal {C}}}}$. Then $g_e\notin \bar{{{\mathcal {C}}}}$.

By Theorem 6.3, $O(G-e,\phi ,p)$ is row independent for any ${{{\mathcal {D}}}}_{2k}$-generic joint configuration $p:V(G)\rightarrow {\mathbb {R}}^2$, and the kernel space of $O(G-e,\phi ,p)$ is one-dimensional.

Let $m:v\in V(G)\mapsto m_i\in {\mathbb {R}}^2$ be a nonzero infinitesimal motion. Also, denote $p_v=p(v)$ for $v\in V(G)$. Then either (i) ${{{\mathcal {C}}}}$ is the group of the reflection along a line *L*, in which case there is a $t\in L$ such that $m_v=t$ for all $v\in V(G)$, or (ii) ${{{\mathcal {C}}}}$ is a group of rotations, in which case $m_v=C_{\pi /2}p_v$ for $v\in V(G)$. We show that *m* does not satisfy the equation associated with $e=(i,j)$:

18 $$\begin{aligned} \langle p_i-g_ep_j,m_i-g_em_j\rangle = 0. \end{aligned}$$

First suppose that ${{{\mathcal {C}}}}$ is the group of the reflection along a line *L*. Then (18) implies

$$\begin{aligned} 0=\langle p_i-g_ep_j,t-g_et\rangle = \langle (I_2-g_e^{-1})p_i+(I_2-g_e)p_j,t\rangle . \end{aligned}$$

Thus $(I_2-g_e^{-1})p_i+(I_2-g_e)p_j\in L^{\bot }$. As *p* is generic, the only possible situation is that $p_i=p_j$ and $g_e$ is the reflection along *L* by Lemma 8.3. This however implies that *G* is cyclic, a contradiction. Thus, *m* does not satisfy (18).

Next suppose that ${{{\mathcal {C}}}}$ is a group of rotations. If *e* is a loop (and hence $p_i=p_j$), then the left side of (18) becomes

$$\begin{aligned} \langle (I_2-g_e)p_i,(I_2-g_e)m_i \rangle = \langle (I_2-g_e)p_i,(I_2-g_e)C_{\pi /2}p_i \rangle . \end{aligned}$$

Note that $g_e$ is a reflection by $g_e\notin \bar{{{\mathcal {C}}}}$, and thus this inner product is nonzero by Lemma 8.3. If *e* is not a loop, by $\langle p_i-g_ep_j,C_{\pi /2}(p_i-g_ep_j)\rangle =0$, (18) becomes

$$\begin{aligned} 0&=\langle p_i-g_ep_j,m_i-g_em_j\rangle = \langle p_i-g_ep_j,C_{\pi /2}p_i-g_eC_{\pi /2}p_j\rangle \\&= \langle p_i-g_ep_j, (C_{\pi /2}g_e-g_eC_{\pi /2})p_j\rangle . \end{aligned}$$

Since *p* is generic and $p_i\ne p_j$, we have $C_{\pi /2}g_e=g_eC_{\pi /2}$. Since $g_e$ is a reflection, $g_eC_{\pi /2}=C_{\pi /2}^{-1}g_e$ holds. These equalities imply $C_{\pi /2}=C_{\pi /2}^{-1}$, a contradiction. $\square$

The next two lemmas show that loop-2-extensions and 2-extensions preserve the independence.

#### **Lemma 8.5**

Let $(G,\phi )$ be a maximum ${{{\mathcal {D}}}}_{2k}$-tight graph with $k\ge 2$ and $(G',\phi ')$ a maximum ${{{\mathcal {D}}}}_{2k}$-tight graph obtained from $(G,\phi )$ by a loop-2-extension. If there is a mapping $p:V(G)\rightarrow {\mathbb {R}}^2$ such that $O(G,\phi ,p)$ is row independent, then there is a mapping $p':V(G')\rightarrow {\mathbb {R}}^2$ such that $O(G',\phi ',p')$ is row independent.

#### *Proof*

We may assume that *p* is ${{{\mathcal {D}}}}_{2k}$-generic. Suppose that $G'$ is obtained from *G* by a loop-2-extension, by removing an existing edge *e*, adding a new vertex *v* with new non-loop edges $e_1$ and $e_2$ and a new loop *l* incident to *v* (see Fig. 5). We may assume that $e_1$ and $e_2$ are outgoing from *v*. Let $u_i$ be the other endvertex of $e_i$ and let $g_i=\phi '(e_i)$ for $i=1,2$. By the definition of loop-2-extension, $\phi (e)=g_1^{-1}g_2$. Also, denote $h=\phi '(l)$.

Let $p_i=p(u_i)$ for $i=1,2$. Note that $g_1p_1\ne g_2p_2$, as $G'$ is ${{{\mathcal {D}}}}_{2k}$-sparse and *p* is ${{{\mathcal {D}}}}_{2k}$-generic. Let *L* be the line through $g_1p_1$ and $g_2p_2$. We take a point $q\in L{\setminus } \{g_1p_1,g_2p_2\}$, and define $p':V(G')\rightarrow {\mathbb {R}}^2$ such that $p'(w)=p(w)$ for each $w\in V(G)$ and $p'(v)=q$. Then $O(G',\phi ',p')$ is described as follows: if $u_1\ne u_2$whereas, if $u_1=u_2$ (and hence $p_1=p_2$),Since $q\in L{\setminus } \{g_1p_1, g_2p_2\}$, $q-g_ip_i$ is a scalar multiple of $g_1p_1-g_2p_2$ for $i=1,2$. Hence, as in the proof of Lemma 6.1, by multiplying the rows of $e_1$ and $e_2$ by appropriate scalars and then subtracting the row of $e_1$ from that of $e_2$, $O(G',\phi ',p')$ becomes one of the following matrices:depending on whether $u_1\ne u_2$ or $u_1=u_2$. The right-bottom block together with the row of $e_2$ forms $O(G,\phi ,p)$, which is row independent. Hence, $O(G',\phi ',p')$ is row independent if and only if $\{(2I_2-h-h^{-1})q, g_1p_1-g_2p_2\}$ is linearly independent. We have the cases where $\{(2I_2-h-h^{-1})q, g_1p_1-g_2p_2\}$ can be linearly dependent.

#### **Claim 8.6**

If there is no point $q\in L{\setminus }\{g_1p_1,g_2p_2\}$ such that $\{(2I_2-h-h^{-1})q, g_1p_1-g_2p_2\}$ is linearly independent, then either

1. $u_1=u_2$ and *h* is the reflection along the line orthogonal to *L* with $h=g_2g_1^{-1}$, or
2. $u_1=u_2$, *h* is a rotation, and $g_2g_1^{-1}$ is the two-fold rotation.

#### *Proof*

We split the proof into two cases.

Suppose that *h* is the reflection along some line *R* through the origin. By Lemma 8.3, $(2I_2-h-h^{-1})q$ is orthogonal to *R*. This means that, if $\{(2I_2-h-h^{-1})q, g_1p_1-g_2p_2\}$ is dependent, *L* is orthogonal to *R*. Since *p* is ${{{\mathcal {D}}}}_{2k}$-generic, *L* cannot be orthogonal to reflection line *R* if $p_1\ne p_2$. Thus, $p_1=p_2$ (and hence $u_1=u_2$), and $h=g_2g_1^{-1}$ as *p* is ${{{\mathcal {D}}}}_{2k}$-generic.

Suppose that *h* is a rotation. By Lemma 8.3, $(2I_2-h-h^{-1})q$ is a scalar multiple of *q*. Hence, if $\{(2I_2-h-h^{-1})q, g_1p_1-g_2p_2\}$ is dependent for any $q\in L{\setminus } \{g_1p_1,g_2p_2\}$, *L* passes through the origin. Since *p* is ${{{\mathcal {D}}}}_{2k}$-generic, *L* passes through the origin if and only if $p_1=p_2$ (and hence $u_1=u_2$) and $g_2p_1$ is the antipodal point of $g_1p_1$. Observe that $g_2p_1$ is the antipodal point of $g_1p_1$ if and only if $g_2g_1^{-1}$ is the two-fold rotation as *p* is ${{{\mathcal {D}}}}_{2k}$-generic. $\square$

By Claim 8.6 we may focus on cases (1) and (2) of Claim 8.6.

Case (1). Suppose that $u_1=u_2$ and *h* is the reflection along *R* with $h=g_2g_1^{-1}$, where *R* is the line orthogonal to *L* and through the origin. Note that $\phi (e)=g_1^{-1}g_2$ is a reflection since $g_2g_1^{-1}$ is a reflection (a conjugate of a reflection is also a reflection).

We take a point $x\in {\mathbb {R}}^2{\setminus } (L\cup R)$ and redefine $p':V(G')\rightarrow {\mathbb {R}}^2$ such that $p'(w)=p(w)$ for each $w\in V(G)$ and $p'(v)=x$. Then, the orbit rigidity matrix becomesBy subtracting the row of $e_1$ from that of $e_2$, it changes toBy Lemma 8.3, $(I_2-h)x$ is orthogonal to *R*. Since *R* is orthogonal to *L*, we deduce that $(I_2-h)x$ is a scalar multiple of $g_1p_1-g_2p_1$. Thus, by subtracting a scalar multiple of the first row, the row of $e_2$ is changed to the following form:Moreover, $g_1^{-1}x-g_2^{-1}x=(I_2-g_2^{-1}g_1)g_1^{-1}x=(I_2-\phi (e))g_1^{-1}x$, which is a scalar multiple of $(2I_2-\phi (e)-\phi (e)^{-1})p_1$ by Lemma 8.3 (using the fact that $\phi (e)$ is a reflection). Thus, by multiplying the row of $e_2$ by another scalar, the matrix is changed towhere the right-bottom block together with the row of $e_2$ forms $O(G,\phi ,p)$, which is row independent, and the left-top block is also row independent as $(I_2-h)x\in R^{\bot }$ and $x-g_1p_1\notin R^{\bot }$ by $x\notin L$ and $g_1p_1\in L$. Thus, $O(G',\phi ',p')$ is row independent.

Case (2). Suppose that $u_1=u_2$, *h* is a rotation, and $g_2g_1^{-1}$ is the two-fold rotation. We redefine $p':V(G')\rightarrow {\mathbb {R}}^2$ such that $p'(w)=p(w)$ for each $w\in V(G)$ and $p'(v)=0$. Let us consider the rank of $O(G',\phi ',p')$. Since $p'(v)=0$, the row of *l* is a zero vector in $O(G',\phi ',q')$. We put $d\in {\mathbb {R}}^2$ in place of $(2I-h-h^{-1})p'(v)$, where *d* is a vector linearly independent from $(g_2-g_1)p_1$, and the resulting matrix is denoted by ${\bar{O}}(G',\phi ',p')$. Then ${\bar{O}}(G',\phi ',p')$ is written in the following way:We first compute the rank of ${\bar{O}}(G',\phi ',p')$. To do this first we recall that $g_2g_1^{-1}$ is the two-fold rotation. This means that *L* contains the origin, and $g_1p_1+g_2p_1=0$. Also, since $\phi (e)=g_1^{-1}g_2$ is a rotation, $p_1$ is proportional to $(2I_2-\phi (e)-\phi (e)^{-1})p_1$ by Lemma 8.3. Therefore, by appropriate row operations, ${\bar{O}}(G',\phi ',p')$ will look like this:where the right-bottom block together with the row of $e_2$ forms $O(G,\phi ,p)$, which is row independent, and the left-top block is also row independent by the choice of *d*. Thus, ${\bar{O}}(G',\phi ',p')$ is row independent.

To avoid the situation where $p'(v)=0$, we continuously perturb $p'(v)$ in the direction of *d*. To see the perturbation more precisely, for each $t\in {\mathbb {R}}$, let us define $p_t':V\cup \{v\}\rightarrow {\mathbb {R}}^2$ by $p_t'(v)=td$ and $p_t'(u)=p'(u)$ for each $u\in V$. Then, observe that for all $t\in {\mathbb {R}}{\setminus } \{0\}$ the row of *l* in $O(G',\phi ',p_t')$ is a nonzero scalar multiple of that of *l* in ${\bar{O}}(G',\phi ',p')$ by Lemma 8.3. Therefore, $\mathrm{rank} \ O(G',\phi ',p_t')=\mathrm{rank}\ {\bar{O}}(G',\phi ',p_t')$ for all $t\in {\mathbb {R}}{\setminus }\{0\}$. Since ${\bar{O}}(G',\phi ',p_0')={\bar{O}}(G',\phi ',p')$ and the latter matrix is row independent, it follows that ${\bar{O}}(G',\phi ',p_t')$ is row independent for almost all *t*. This in turn implies that $O(G',\phi ',p_t')$ is row independent for almost all $t\in {\mathbb {R}}{\setminus } \{0\}$.

This completes the proof of the lemma. $\square$

#### **Lemma 8.7**

Let $(G,\phi )$ be a maximum ${{{\mathcal {D}}}}_{2k}$-tight graph with $k\ge 2$ and $(G',\phi ')$ a maximum ${{{\mathcal {D}}}}_{2k}$-tight graph obtained from $(G,\phi )$ by a 2-extension. If there is a mapping $p:V(G)\rightarrow {\mathbb {R}}^2$ such that $O(G,\phi ,p)$ is row independent, then there is a mapping $p':V(G')\rightarrow {\mathbb {R}}^2$ such that $O(G',\phi ',p')$ is row independent.

#### *Proof*

We may assume that *p* is ${{{\mathcal {D}}}}_{2k}$-generic. Suppose that $G'$ is obtained from *G* by a 2-extension, by removing two existing edges *e* and *f* and adding a new vertex *v* with new non-loop edges $e_1, e_2,e_3,e_4$ incident to *v* (see Fig. 4). We may assume that $e_i$ is outgoing from *v*, and $e=e_1^{-1}\cdot e_2$ and $f=e_3^{-1}\cdot e_4$. Let $u_i$ be the other endvertex of $e_i$ and let $g_i=\phi '(e_i)$. We then have $\phi (e)=g_1^{-1}g_2$ and $\phi (f)=g_3^{-1} g_4$.

Let $p_i=p(u_i)$ for $i=1,\ldots , 4$, *L* be the line through $g_1p_1$ and $g_2p_2$, and $L'$ be the line through $g_3p_3$ and $g_4p_4$. We have the following elementary geometric observation.

#### **Claim 8.8**

(i) No three points among $\{g_ip_i:i=1,\ldots , 4\}$ are collinear.

(ii) If *L* and $L'$ are parallel, then the following holds:

- $$L\ne L'$$,
- $$u_1=u_2$$ and $$u_3=u_4$$, and
- $g_2g_1^{-1}$ is the reflection along $L^{\bot }$ with $g_2g_1^{-1}=g_4g_3^{-1}$.

#### *Proof*

The first claim follows from the proof of Claim 6.2.

For the second claim, suppose that *L* and $L'$ are parallel. Without loss of generality, we have the following four cases: (i) $p_1\notin \{ p_2,p_3,p_4\}$, (ii) $p_1=p_2=p_3=p_4$, (iii) $p_1=p_2\ne p_3=p_4$, and (iv) $p_1=p_3\ne p_2=p_4$.

In case (i), the ${{{\mathcal {D}}}}_{2k}$-genericity of *p* implies that $g_1p_1$ has no relation to the other three points, and hence *L* and $L'$ intersect at a point.

In case (ii), $g_1p_1,\ldots , g_4p_4$ lie on a circle *C*. Moreover, since $u_1=u_2=u_3=u_4$, *e* and *f* are loops attached to a vertex (i.e., the 2-extension is type (g) of Fig. 4). This implies that the group generated by $\{g_1^{-1}g_2, g_3^{-1}g_4\}$ is not cyclic by the ${{{\mathcal {D}}}}_{2k}$-sparsity of *G*.

Now, *L* is the line through $g_1p_1$ and $g_2p_1$ while $L'$ is the line through $g_3p_1$ and $g_4p_1$. We have two subcases depending on whether $g_2g_1^{-1}$ is a reflection or a rotation.

(ii-1) If $g_2g_1^{-1}$ is a reflection, then it is the reflection along the bisector $L^{\bot }$ of $g_1p_1$ and $g_2p_1$. If *L* and $L'$ are parallel, then this reflection also sends $g_3p_3$ to $g_4p_4$. This means that $g_2g_1^{-1}$ is the reflection along $L^{\bot }$ with $g_2g_1^{-1}=g_4g_3^{-1}$, which implies the statement.

(ii-2) If $g_2g_1^{-1}$ is a rotation, $g_1p_1$ and $g_2p_1$ are vertices of a regular *k*-gon inscribing *C*. Since *p* is generic, if $L'$ is parallel to *L*, $g_3p_1$ and $g_4p_1$ are also vertices of this regular *k*-gon, and hence $g_4g_3^{-1}$ is also a rotation. Since a conjugate of a rotation is also a rotation, we deduce that $g_1^{-1}g_2$ and $g_3^{-1}g_4$ are rotations as well. This however contradicts the fact that $\langle g_1^{-1}g_2,g_3^{-1}g_4\rangle$ is not cyclic.

In case (iii), *L* is the line through $g_1p_1$ and $g_2p_1$ while $L'$ is the line through $g_3p_3$ and $g_4p_3$. Observe that, if $g_2g_1^{-1}$ is a rotation, the line *L* can have any slope, by moving $p_1$. Therefore, if *L* and $L'$ are parallel for generic *p*, then $g_2g_1^{-1}$ and $g_4g_3^{-1}$ are both reflections. When $g_2g_1^{-1}$ is a reflection, it is the reflection along the bisector $L^{\bot }$ of $g_1p_1$ and $g_2p_1$. As $g_4g_3^{-1}$ is a reflection and *L* and $L'$ are parallel, $g_4g_3^{-1}$ is the reflection along $L^{\bot }$, which implies the statement.

In case (iv), *L* is the line through $g_1p_1$ and $g_2p_2$ and $L'$ is the line through $g_3p_1$ and $g_4p_2$. Also, $u_1=u_3\ne u_2=u_4$ implies that $\{e, f\}$ forms a 2-cycle in $G'$ (i.e, the 2-extension is type (f) in Fig. 4). Hence, $\phi (e)\ne \phi (f)$, or equivalently, $g_1^{-1}g_2\ne g_3^{-1}g_4$. This implies

19 $$\begin{aligned} g_1g_3^{-1}\ne g_2g_4^{-1}. \end{aligned}$$

We prove that *L* and $L'$ cannot be parallel if *p* is generic.

**Fig. 10:**
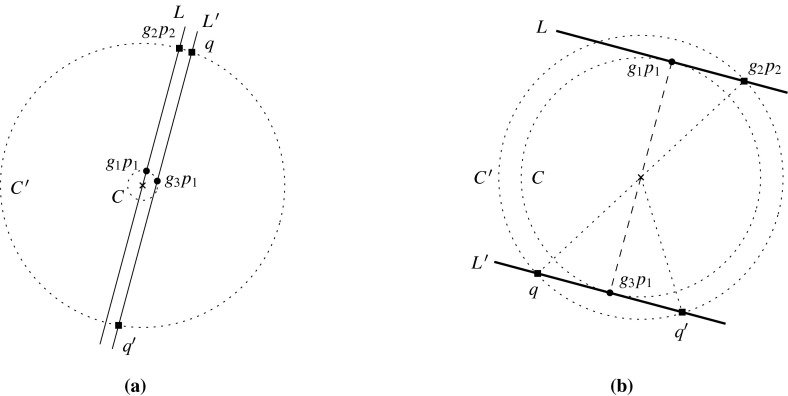
Proof of case (iv) in Claim 8.8

Let *C* be the circle whose center is the origin and which passes through $g_1p_1$ (and hence through $g_3p_1$). We split the proof into two cases depending on whether $g_3g_1^{-1}$ is the two-fold rotation $C_{\pi }$ or not.

(iv-1) Suppose that $g_3g_1^{-1}\ne C_{\pi }$. Let $C'$ be a circle whose center is the origin and the diameter is much larger than that of *C*. We shall relocate $g_2p_2$ on $C'$ such that $g_2p_2$ is on the line through $g_1p_1$ and the origin as shown in Fig. 10a. Then, if *L* and $L'$ are parallel, we have only two possible locations *q* and $q'$ for $g_4p_2$ (as shown in Fig. 10a). Since the diameter of $C'$ can be arbitrarily large, ${{{\mathcal {D}}}}_{2k}$ has no element that sends $g_2p_2$ to *q* or $q'$. In other words, if *p* is generic, *L* and $L'$ are not parallel.

(iv-2) Suppose that $g_3g_1^{-1}=C_{\pi }$. Then $g_3p_1$ is the antipodal point of $g_1p_1$ in *C* as shown in Fig. 10b. Let $C'$ be a circle whose center is the origin and the diameter is slightly larger than that of *C*. We shall relocate $g_2p_2$ on $C'$ such that *L* is the tangent of *C* at $g_1p_1$ (see Fig. 10b). Then, we have only two possible locations *q* and $q'$ for $g_4p_2$ as *L* and $L'$ are parallel and $g_4p_2$ is on $C'$, where *q* is the antipodal point of $g_2p_2$ with respect to the origin and $q'$ is the reflection of $q_2p_2$ along the line parallel to *L* and through the origin. When *p* is generic, *L* is not parallel to any reflection lines in ${{{\mathcal {D}}}}_{2k}$, implying $g_4p_2\ne q'$. Hence, $g_4p_2=q$. This means that $g_4g_2^{-1}$ is also the two-fold rotation $C_{\pi }$.

Recall that $C_{\pi }$ is in the center of ${{{\mathcal {O}}}}({\mathbb {R}}^2)$, i.e., $gC_{\pi }=C_{\pi }g$ for any $g\in {{{\mathcal {O}}}}({\mathbb {R}}^2)$. Hence, by $g_3g_1^{-1}=C_{\pi }$, we have $g_1^{-1}g_3=g_1^{-1}C_{\pi }g_1=C_{\pi }$. Symmetrically, by $g_4g_2^{-1}=C_{\pi }$, we have $g_2^{-1}g_4=C_{\pi }$. This however implies that $g_1^{-1}g_3=g_2^{-1}g_4$, which contradicts (19). $\square$

Following the statement of Claim 8.8, we shall split the proof into two cases.

(Case 1) Suppose that *L* and $L'$ are not parallel. Let *q* be the intersection of *L* and $L'$. By Claim 8.8(i), we have $q\ne g_ip_i$. We define $p':V(G')\rightarrow {\mathbb {R}}^2$ by $p'(w)=p(w)$ for $w\in V(G)$ and $p'(v)=q$ for the added vertex *v*. Then, $O(G',\phi ',p')$ can be written as follows:where $O(G-e-f,\phi ,p)$ is the matrix obtained from $O(G,\phi ,p)$ by removing the rows of *e* and *f*. Consider the rows associated with $e_1$ and $e_2$. Since *q* is on *L*, $q-g_ip_i$ is a scalar multiple of $g_1p_1-g_2p_2$, and hence these two rows can be transformed to the following form by row operations: if $u_1\ne u_2$and, if $u_1=u_2$,Notice that, in each case, the row of $e_2$ is converted to that of *e* in $O(G,\phi ,p)$. In a symmetric manner, the rows of $e_3$ and $e_4$ can be converted to the above form, simply by replacing 1 and 2 with 3 and 4, respectively. Thus, $O(G',\phi ',p')$ is converted toThe right-bottom block $O(G,\phi ,p)$ is row independent while the left-top block is also row independent since *L* and $L'$ are not parallel. In other words, $O(G',\phi ',p')$ is row independent.

(Case 2) Suppose that *L* and $L'$ are parallel. By Claim 8.8, $L\ne L'$, $p_1=p_2$, $p_3=p_4$, and $g_1^{-1}g_2$ and $g_3^{-1}g_4$ are reflections. Let *q* be any point on *L* with $q\ne g_1p_1$ and $q\ne g_2p_1$. We define $p':V(G')\rightarrow {\mathbb {R}}^2$ by $p'(w)=p(w)$ for $w\in V(G)$ and $p'(v)=q$ for the new vertex *v*. Then, the orbit rigidity matrix is described as follows:Since *q* is on the line *L*, $q-g_ip_i$ is a scalar multiple of $(g_1-g_2)p_1$ for $i=1,2$. Hence, the rows of $e_1$ and $e_2$ can be converted toand then toSince $g_1^{-1}g_2$ is a reflection, we have $g_1^{-1}g_2=g_2^{-1}g_1$. Hence, by adding the half of the second row to the first row, we obtainNext we consider the rows of $e_3$ and $e_4$. By subtracting the row of $e_3$ from that of $e_4$, we obtainSince *L* and $L'$ are parallel, $\{(g_1-g_2)p_1, (g_3-g_4)p_3\}$ is linearly dependent. Thus, by subtracting the row of $e_1$ from that of $e_4$, we haveMoreover, since $g_4^{-1}g_3$ is a reflection, Lemma 8.3 implies that $(I_2-g_4^{-1}g_3)g_3^{-1}q$ is a scalar multiple of $(I_2-g_4^{-1}g_3)p_3$, and hence $(g_3^{-1}-g_4^{-1})q$ is a scalar multiple of $(I_2-g_4^{-1}g_3)p_3$. Therefore, by using $g_3^{-1}g_4=g_4^{-1}g_3$, the row of $e_4$ can be converted by a scalar multiplication toIn total, $O(G',\phi ',p')$ is changed to the following form by row-operations: The right-bottom block together with the rows of $e_2$ and $e_4$ forms $O(G,\phi ,p)$, which is row independent. Also, since *q* is on *L*, but not on $L'$, $\{(g_1-g_2)p_1,q-g_3p_3\}$ is linearly independent. Therefore, $O(G',\phi ',p)$ is row independent. $\square$

Combining Theorem 7.12, Lemmas 6.1, 8.1, 8.4, 8.5, and 8.7, we can now complete the proof of Theorem 8.2.

### Symmetry-Forced Infinitesimal Motions with ${{{\mathcal {D}}}}_{2k}$-Symmetry for Even *k*

Notice that all the lemmas given in the last subsection are independent of the parity of *k*. Therefore, we obtain the following statement even for a dihedral group ${{{\mathcal {D}}}}_{2k}$ with even *k*: for a generic $({{{\mathcal {D}}}}_{2k},\rho )$-symmetric framework (*H*, *p*) with even *k* and a free action $\rho$, (*H*, *p*) is symmetry-forced infinitesimally rigid if the quotient gain graph can be constructed from a disjoint union of base graphs by 0-extensions, 1-extensions, loop-1-extensions, 2-extensions and loop-2-extensions. However, as we have seen in Fig. 9, there are infinitely many gain graphs that cannot be constructed from base graphs. By Theorem 7.9, minimal examples are ${{{\mathcal {D}}}}_{2k}$-sparse double cycles $C_{n}^2$. Below, we show that some of them indeed have symmetric infinitesimal motions.

For $C_n^2$, the vertex set is denoted by $\{1,\ldots , n\}$ and the edges of the 2-cycle between *i* and $i+1\ (\mathrm{mod}\ n)$ are denoted by $e_{i,1}$ and $e_{i,2}$ for $i=1,\ldots , n$.

#### **Theorem 8.9**

Let ${{{\mathcal {D}}}}_4$ be the dihedral group of order 4, which consists of the identity $I_2$, the two-fold rotation $C_{\pi }$, and two reflections *r* and $r'$. Let $(G,\phi )$ be a ${{{\mathcal {D}}}}_4$-sparse $C_n^2$ such that

- $$\phi (e_{i,1})=\mathrm{id}$$ and $$\phi (e_{i,2})=r'$$ for $$i=1,\ldots , n-1$$;
- $$\phi (e_{n,1})=C_{\pi }$$ and $$\phi (e_{n,2})=r$$.

Then, for any ${{{\mathcal {D}}}}_4$-generic $p:V(G)\rightarrow {\mathbb {R}}^2$, ${{\mathrm{rank}}}O(G,\phi ,p)=2n$ if and only if *n* is odd.

#### *Proof*

Let $p:i\in V(G)\mapsto (x_i,y_i)\in {\mathbb {R}}^2$ be a ${{{\mathcal {D}}}}_4$-generic mapping. Then $C_{\pi }p(i)=(-x_i,-y_i)$, $rp(i)=(-x_i,y_i)$, $r'p(i)=(x_i,-y_i)$. The rows of $O(G,\phi ,p)$ are as follows:andwhere the left and the right half sides correspond to *x*- and *y*-coordinates, respectively. For each *i*, we subtract the first row from the second row and then multiply the first row by an appropriate scalar. We then have, for each $i=1,\ldots , n-1$,andIn other words, $O(G,\phi ,p)$ is converted to the following form:The determinant of this matrix is $2(1-(-1)^{n-1})\prod _{i=1}^n y_i$, which is equal to zero if and only if *n* is even. $\square$

**Fig. 11:**
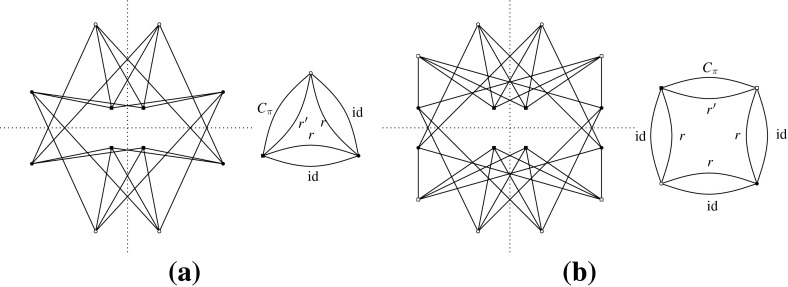
Examples of symmetric frameworks given in Theorem 8.9, where **b** has a symmetric infinitesimal motion while **a** does not

See Fig. 11 for examples of frameworks given in Theorem 8.9. For $n=2$, the covering graph is $K_{4,4}$ and the corresponding framework is known as Bottema’s mechanism (see [23, Sect. 7.2.1]).

## Proof of Theorem 7.8

In this section we prove Theorem 7.8. For simplicity of the description, a ${{{\mathcal {D}}}}$-gain graph is called *essential* if it is ${{{\mathcal {D}}}}$-sparse, 4-regular, not a base graph, and not a double cycle. Lemma 7.6 shows that 2-extensions and loop-2-extensions preserve ${{{\mathcal {D}}}}$-sparsity, and hence what we have to prove is the following theorem.

### **Theorem 9.1**

Any essential ${{{\mathcal {D}}}}$-gain graph $(G,\phi )$ has a vertex at which a 2-reduction or a loop-2-reduction is admissible.

For simplicity, in the subsequent discussion we omit gain functions $\phi$ when referring to gain graphs if it is clear from the context. Also an edge (*u*, *v*) from *u* to *v* is simply denoted by *uv*, and a ${{{\mathcal {D}}}}$-tight set is called a tight set.

The proof of Theorem 9.1 consists of four parts. In Sect. 9.1, we shall prove useful lemmas for subsequent discussion. In Sect. 9.2, we prove Theorem 9.1 for the following graphs:

- graphs consisting of only *special* vertices (Lemma 9.5), where a vertex is called special if it is incident with a loop or two parallel classes of edges;
- graphs that are not 2-connected (Lemma 9.6),
- “almost” near-cyclic graphs (Lemma 9.8), defined below,
- graphs that are not essentially 4-edge-connected (Lemma 9.9),
- graphs having a vertex *v* with $|N(v)|=2$.

In Sect. 9.3 we discuss graphs not belonging to the above classes. In Sect. 9.4 we put everything together to complete the proof of Theorem 9.1.

### Preliminary Facts

The following fundamental properties of 4-regular graphs will be frequently used.

- A 4-regular graph is Eulerian. Hence, a 4-regular connected graph is 2-edge-connected.
- Let $G=(V,E)$ be a graph with maximum degree at most 4. Then, for any $X\subseteq V$, $i_G(X)\le 2|X|-\lfloor d_G(X)/2\rfloor$, where $i_G(X)$ denotes the number of edges induced by *X*. In particular, if *G* is 4-regular, $i_G(X)=2|X|-d_G(X)/2$.

The next lemma asserts that if the maximum degree is at most 4, then ${{{\mathcal {D}}}}$-sparsity is equivalent to the following simpler properties:

(C1): $|F|\le 2|V(F)|-3$ for every nonempty balanced set $F\subseteq E$;
(C2): *G* is not cyclic.

#### **Lemma 9.2**

Let $G=(V,E)$ be a ${{{\mathcal {D}}}}$-gain graph with maximum vertex degree at most 4. If *G* is connected, then *G* is ${{{\mathcal {D}}}}$-sparse if and only if

(i) *G* is not 4-regular and condition (C1) is satisfied, or
(ii) *G* is 4-regular and conditions (C1) and (C2) are satisfied.

If *G* is not connected, then *G* is ${{{\mathcal {D}}}}$-sparse if and only if each connected component is ${{{\mathcal {D}}}}$-sparse.

#### *Proof*

If the maximum degree is at most 4, $|F|\le 2|V(F)|$ for any $F\subseteq E$. In particular, if *G* is connected, we have $|F|\le i_G(V(F)) \le 2|V(F)|-\lfloor d_G(V(F))/2 \rfloor \le 2|V(F)|-1$ for any $F\subseteq E$ with $V(F)\ne V$. Therefore, $|F|\ge 2|V(F)|$ holds if and only if *G* is 4-regular and $F=E$. $\square$

Thus, to prove Theorem 9.1, we shall investigate whether (C1) and (C2) are satisfied after the reductions. The next lemma will be used when (C2) is not satisfied. We say that $(G,\phi )$ is *almost near-cyclic* if there are two incident edges *e* and *f* such that $G-e-f$ is cyclic.

#### **Lemma 9.3**

Let $(G,\phi )$ be a connected 4-regular ${{{\mathcal {D}}}}$-sparse graph with $G=(V,E)$ and *v* be a vertex in *G* that is not incident to a loop. Let $e_1,e_2,e_3,e_4$ be the edges incoming to *v*, and suppose that $G-v+e_1\cdot e_2^{-1}+e_3\cdot e_4^{-1}$ is connected and cyclic. Then, there is an equivalent gain function $\phi '$ to $\phi$ and a cyclic subgroup ${{{\mathcal {C}}}}$ of ${{{\mathcal {D}}}}$ such that

- $\phi '(e)\in {{{\mathcal {C}}}}$ for every $e\in E{\setminus } \{e_3,e_4\}$, and
- $$\phi '(e_3)\notin \bar{{{\mathcal {C}}}}$$ and $$\phi '(e_4)\notin \bar{{{\mathcal {C}}}}$$.

In particular, *G* is almost near-cyclic.

#### *Proof*

Let $G'=G-v+e_1 \cdot e_2^{-1}+e_3\cdot e_4^{-1}$. Since $G'$ is connected and cyclic, by Lemma 2.3, there is an equivalent gain function $\phi '$ to $\phi$ and a cyclic subgroup ${{{\mathcal {C}}}}$ of ${{{\mathcal {D}}}}$ such that $\phi '(e)\in {{{\mathcal {C}}}}$ for all $e\in E(G')$. Let $a=\phi '(e_1\cdot e_2^{-1})\in {{{\mathcal {C}}}}$ and $a'=\phi '(e_3\cdot e_4^{-1})\in {{{\mathcal {C}}}}$. Then, by using some elements $b_1,b_2\in {{{\mathcal {D}}}}$, we can express $\phi '(e_i)$ by

$$\begin{aligned} \phi '(e_1)=ab_1, \quad \phi '(e_2)=b_1,\quad \phi '(e_3)=a'b_2,\quad \phi '(e_4)=b_2. \end{aligned}$$

We further perform a switching operation at *v* with $b_1$. We consequently have an equivalent gain function $\phi '$ to $\phi$ such that

$$\begin{aligned} \phi '(e_1)=a,\quad \phi '(e_2)=\mathrm{id},\quad \phi '(e_3)=a'b,\quad \phi '(e_4)=b, \end{aligned}$$

where $b=b_2b_1^{-1}$. Notice that $\phi '(e)\in {{{\mathcal {C}}}}$ for all $e\in E{\setminus } \{e_3,e_4\}$. Since *G* is not cyclic, we must have $b\notin \bar{{{\mathcal {C}}}}$, implying that $\phi '(e_3)\notin \bar{{{\mathcal {C}}}}$ and $\phi '(e_4)\notin \bar{{{\mathcal {C}}}}$. $\square$

The following technical lemma is one of the key observations. A vertex in a 4-regular graph is called *special* if it is incident with a loop or two parallel classes of edges with $|N(v)|=2$.

#### **Lemma 9.4**

Let $(G,\phi )$ be a connected 4-regular ${{{\mathcal {D}}}}$-sparse graph with $G=(V,E)$ and $|V|\ge 3$, *v* be a vertex in *G* that is not special, and $e_1,e_2,e_3,e_4$ be the edges incoming to *v*. If $G-e_3-e_4$ or $G-v+e_1\cdot e_2^{-1}+e_3\cdot e_4^{-1}$ is connected and cyclic, then at least one of the following holds:

- *G* is near-cyclic.
- $$G-v+e_1\cdot e_3^{-1}+e_2\cdot e_4^{-1}$$ is $${{{\mathcal {D}}}}$$-sparse.
- *v* is a cut-vertex in *G* and $G-v+e_1\cdot e_3^{-1}+e_2\cdot e_4^{-1}$ is connected.

#### *Proof*

For simplicity, we denote $e_{i,j}=e_i\cdot e_j^{-1}$ for $i,j\in \{1,2,3,4\}$. We assume that neither (a) nor (b) occur and show that (c) holds.

We claim that there are an equivalent gain function $\phi '$ to $\phi$ and a cyclic subgroup ${{{\mathcal {C}}}}$ of ${{{\mathcal {D}}}}$ such that $\phi '(e)\in {{{\mathcal {C}}}}$ holds for $e\in E{\setminus } \{e_3,e_4\}$ and $\phi '(e_3)\notin \bar{{{\mathcal {C}}}}$ and $\phi '(e_4)\notin \bar{{{\mathcal {C}}}}$.

To see this, first observe that if $G-v+e_1\cdot e_2^{-1}+e_3\cdot e_4^{-1}$ is connected and cyclic, then Lemma 9.3 implies the claim. On the other hand, if $G-e_3-e_4$ is connected and cyclic, then by Lemma 2.3, there is an equivalent $\phi '$ to $\phi$ and a cyclic subgroup ${{{\mathcal {C}}}}$ of ${{{\mathcal {D}}}}$ such that $\phi '(e)\in {{{\mathcal {C}}}}$ for $e\in E{\setminus } \{e_3,e_4\}$. Since *G* is neither cyclic nor near-cyclic, we have $\phi '(e_3)\notin \bar{{{\mathcal {C}}}}$, and $\phi '(e_4)\notin \bar{{{\mathcal {C}}}}$.

Note that $\phi '(e_{1,3})\notin \bar{{{\mathcal {C}}}}$ and $\phi '(e_{2,4})\notin \bar{{{\mathcal {C}}}}$.

Let us consider $G-v$. Since $G-v$ is cyclic with $|E(G-v)|=2|V(G-v)|-2$, $G-v$ is (2, 3)-g-sparse. Applying Lemma 7.7 with $\phi '(e_{1,3})\notin \bar{{{\mathcal {C}}}}$, we deduce that $G-v+e_{1,3}$ is ${{{\mathcal {D}}}}$-sparse. Let $G'=G-v+e_{1,3}+e_{2,4}$. Since (b) does not hold, $G'$ is not ${{{\mathcal {D}}}}$-sparse. By Lemma 9.2, $G'$ (or a connected component of $G'$) violates (C1) or (C2).

Case 1: If (C1) is violated, then $G-v+e_{1,3}$ contains a balanced tight set *F* such that *V*(*F*) contains the endvertices of $e_{2,4}$ and $F+e_{2,4}$ is balanced. Let *s* and *t* be the endvertices of $e_{2,4}$, which are possibly the same vertex. By Lemma 7.1, if $|F|>1$, *F* contains a path from *s* to *t* that does not pass through $e_{1,3}$. Recall that the gain of each edge in this path is included in ${{{\mathcal {C}}}}$, and the concatenation of the path and $e_{2,4}$ forms an unbalanced closed walk in $F+e_{2,4}$, contradicting that $F+e_{2,4}$ is balanced. Therefore, $|F|=1$ holds; in particular, since $s,t\in V(F)$ and $F+e_{2,4}$ is balanced, it follows that $F=\{e_{1,3}\}$ and $\{e_{1,3},e_{2,4}\}$ forms a balanced 2-cycle in $G'$. This implies that *v* is special in *G*, contradicting the assumption of the lemma.

Case 2: We next consider the case when (C2) is violated in $G'$. Suppose that *v* is not a cut-vertex. Note that, since $|E(G-v)|=2|V(G-v)|-2$, $G-v$ contains an unbalanced cycle *C*, whose gain is included in ${{{\mathcal {C}}}}$. Let *s* and *t* be the endvertices of $e_{2,4}$, which are possibly the same vertex. Since $G-v$ is connected, there is a path *P* from *s* to a vertex in *V*(*C*). We consider a closed walk $W_1$ that first passes through *P* starting at *s*, then goes around *C*, and comes back to *s* through $P^{-1}$. We then have $\phi '(W_1)\in {{{\mathcal {C}}}}$. Also, since $G-v$ is connected, $G-v$ has a path $P'$ connecting *s* and *t*. The concatenation of $P'$ with $e_{2,4}$ forms a closed walk $W_2$ starting at *s* with $\phi (W_2)\notin \bar{{{\mathcal {C}}}}$. Thus, $\{\phi '(W_1), \phi '(W_2)\}$ generates a non-cyclic group. Hence, $G'$ satisfies (C2), a contradiction. Thus, *v* is a cut-vertex in *G*.

Suppose that $G'$ is not connected. Then, by the 4-regularity of *G*, $G'$ consists of two connected components, denoted $G_1'$ and $G_2'$ with $e_{1,3}\in E(G_1')$ and $e_{2,4}\in E(G_2')$. We have already seen that $G-v+e_{1,3}$ is ${{{\mathcal {D}}}}$-sparse, and hence its subgraph $G_1'$ is ${{{\mathcal {D}}}}$-sparse. However, since $G_1'$ is 4-regular, $G_1'$ is indeed maximum ${{{\mathcal {D}}}}$-tight. By the symmetry between $e_{1,3}$ and $e_{2,4}$, $G_2'$ is also maximum ${{{\mathcal {D}}}}$-tight, and thus $G'$ is maximum ${{{\mathcal {D}}}}$-tight, a contradiction. Thus $G'$ should be connected, which implies (c). $\square$

### Special Cases

**Fig. 12:**
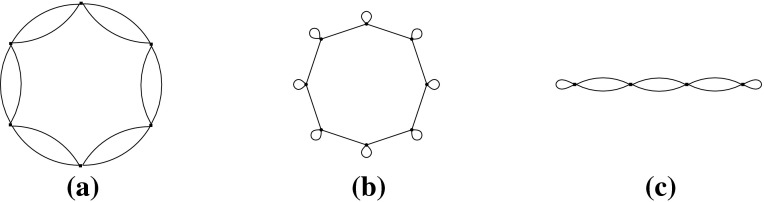
Special graphs: **a** $$C_6^{2}$$. **b** $$C_8^{\circ }$$. **c** $$P_4^{2}$$

Recall that a vertex is said to be special if it is incident with a loop or two parallel classes of edges. A graph which consists of only special vertices is called a *special graph*. Special graphs are classified into the following three classes $C_n^{2}$, $C_n^{\circ }$ and $P_n^{2}$ for $n\ge 2$ (Fig. 12): As defined in Sect. 7.2, $C_n^{2}$ is the graph obtained from the cycle of *n* vertices by replacing each edge by two parallel copies; $C_n^{\circ }$ is the cycle of *n* vertices, each of which is incident to a loop; $P_n^{2}$ is the graph obtained from a path of *n* vertices by replacing each edge by two parallel copies and adding one loop to each endvertex of the path.

#### **Lemma 9.5**

Let $(G,\phi )$ be an essential ${{{\mathcal {D}}}}$-gain graph whose underlying graph $G=(V,E)$ is special. Then there is a vertex at which a 2-reduction or a loop-2-reduction is admissible.

#### *Proof*

Since $(G,\phi )$ is essential, the underlying graph is either $P_n^2$ or $C_n^{\circ }$.

Suppose that the underlying graph is $P_n^{2}$. We perform the loop-2-reduction at a vertex incident to a loop *l*. The resulting graph is $P_{n-1}^{2}$ and clearly it satisfies (C1). If it does not satisfy (C2), then the resulting graph is cyclic and there is a cyclic subgroup ${{{\mathcal {C}}}}$ of ${{{\mathcal {D}}}}$ such that the gain of every cycle in *G* except for the loop *l* is in ${{{\mathcal {C}}}}$. This in turn implies that $G-l$ is cyclic, contradicting the assumption that *G* is essential.

Suppose that the underlying graph is $C_n^{\circ }$. We may assume $n\ge 3$ since $C_2^{\circ }=P_2^{2}$. We perform the 2-reduction at a vertex incident to a loop *l*. The resulting ${{{\mathcal {D}}}}$-gain graph, denoted $G'$, has the underlying graph $C_{n-1}^{\circ }$.

If $G'$ does not satisfy (C2), then the gain of each cycle in *G* except for the loop *l* is included in a cyclic subgroup ${{{\mathcal {C}}}}$ of ${{{\mathcal {D}}}}$, which again contradicts the fact that *G* is essential.

It can be easily observed that $G'$ satisfies (C1) if $n>3$. For $n=3$, (C1) is violated if the 2-cycle of $G'$ is balanced, but in such a case the triangle in the original graph *G* is balanced, and *G* turns out to be a fancy triangle, contradicting the fact that *G* is essential. $\square$

The next lemma solves the case when the graph is not 2-connected.

#### **Lemma 9.6**

Let $G=(V,E)$ be a connected essential ${{{\mathcal {D}}}}$-gain graph with $|V|\ge 2$. Suppose that *G* is not 2-connected. Then a 2-reduction is admissible at some vertex.

#### *Proof*

By Lemma 9.5, we may assume that *G* is not equal to $P_{|V|}^{2}$. Then *G* has a cut-vertex *v* which is not special. We show that a 2-reduction at *v* is admissible. Note that $G-v$ consists of two connected components by the 4-regularity of *G*. Let $e_1,e_7,e_3,e_4$ be the edges incident to *v*, all of them are directed to *v*. From the 2-edge-connectivity of *G*, we can assume, without loss of generality, that the endvertices of $e_1$ and $e_3$ are included in a connected component of $G-v$ while those of $e_2$ and $e_4$ are included in the other component.

Consider the 2-reduction at *v* through $(e_1,e_2)$ and $(e_3,e_4)$. Let $G'$ be the resulting graph. Note that $G'$ is connected. Let us check that $G'$ satisfies (C1). To see this, recall that any balanced tight set consisting of more than one edge is 2-connected by Lemma 7.1. Note also that $e_3\cdot e_4^{-1}$ is not parallel to $e_1\cdot e_2^{-1}$ as *v* is not special. Since the endvertices of $e_3\cdot e_4^{-1}$ belong to different connected components in $G-v$ and $e_1\cdot e_2^{-1}$ is the bridge in $G-v+e_1\cdot e_2^{-1}$, $G-v+e_1\cdot e_2^{-1}$ has no balanced tight set *F* such that *V*(*F*) contains both endvertices of $e_3\cdot e_4^{-1}$. This implies that $G'$ satisfies (C1).

Therefore, if $G'$ satisfies (C2), then $G'$ is ${{{\mathcal {D}}}}$-sparse by Lemma 9.2, and a 2-reduction is admissible at *v*. Suppose that $G'$ does not satisfy (C2). Then, $G'$ is connected and cyclic. To apply Lemma 9.4, we next consider the 2-reduction at *v* through $(e_1,e_3)$ and $(e_2,e_4)$. The resulting graph, denoted by $G''$, is disconnected. Lemma 9.4 thus implies that $G''$ is ${{{\mathcal {D}}}}$-sparse (recall that *G* is not near-cyclic since *G* is essential). $\square$

Thus, in the subsequent discussion, we may focus on 2-connected graphs. The next lemma solves the case when *G* has a special vertex not incident to a loop.

#### **Lemma 9.7**

Let $G=(V,E)$ be a 2-connected essential ${{{\mathcal {D}}}}$-gain graph. Suppose that *G* has a special vertex not incident to a loop. Then, *G* has a vertex at which a 2-reduction is admissible.

#### *Proof*

**Fig. 13:**
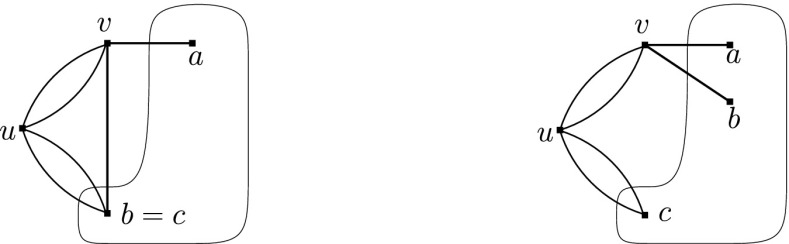
Proof of Lemma 9.7

Let *w* be a special vertex not incident to a loop. By definition of special vertices, $|N(w)|=2$ and *w* is incident to two parallel classes of edges. Since $G\ne C_n^{2}$, *G* contains two adjacent vertices *u* and *v* such that *v* is not special and *u* is special and not incident to a loop (where *u* is possibly equal to *w*). Depending on the size of $N(\{u,v\})$, we have two possible cases as shown in Fig. 13.

Let us denote the edges incident to *u* by $e_1, e_2,e_3,e_4$, where $e_1$ and $e_2$ are linking from *v* to *u* and $e_3$ and $e_4$ are linking from a vertex in $V{\setminus } \{u,v\}$ to *u*. We perform the 2-reduction at *u* through $(e_1,e_2)$ and $(e_3,e_4)$. Since both new edges are unbalanced loops and adding unbalanced loops does not violate (C1), the resulting graph $G'$ satisfies (C1). Therefore, if the 2-reduction is not admissible at *u*, then $G'$ does not satisfy (C2), and hence $G-e_1-e_2$ is cyclic by Lemma 9.3.

Let $a,b,c\in V$ such that $N(v)=\{u,a,b\}$ and $N(u)=\{v,c\}$. Since $|N(u)|=2$ with $v\in N(u)$, without loss of generality we may assume $a\notin N(u)$ (where $b=c$ possibly holds). Recall that $G-e_1-e_2$ is connected and cyclic, and hence we can apply Lemma 9.4 to deduce that the 2-reduction at *v* through $(bv, e_1)$ and $(av, e_2)$ is admissible. Indeed, since *G* is not near-cyclic and *v* is neither a cut-vertex nor a special vertex, Lemma 9.4 implies that this 2-reduction at *v* is admissible. $\square$

The next lemma solves the case when *G* is almost near-cyclic.

#### **Lemma 9.8**

Let $G=(V,E)$ be a 2-connected essential ${{{\mathcal {D}}}}$-gain graph with at least two vertices. Suppose that *G* is almost near-cyclic. Then a 2-reduction or a loop-2-reduction is admissible at some vertex in *G*.

#### *Proof*

Since *G* is almost near-cyclic, there are two edges $e_1$ and $e_2$ for which $e_1$ and $e_2$ are incident to a vertex *v* and $G-e_1-e_2$ is cyclic.

Suppose that *v* is not special. Then, since *v* is not a cut-vertex, a 2-reduction is admissible at *v* by Lemma 9.4. Therefore, let us consider the case when *v* is special. If *v* is not incident to a loop, then Lemma 9.7 directly implies the claim. We can thus assume that *v* is incident to a loop.

Suppose that both $e_1$ and $e_2$ are non-loop edges. By Lemma 2.3, we may assume that the label of each edge in $G-e_1-e_2$ is contained in a cyclic subgroup ${{{\mathcal {C}}}}$ of ${{{\mathcal {D}}}}$. By further performing a switching operation at *v* with $\phi (e_1)$, $\phi$ is converted such that $\phi (e_1)=\mathrm{id}$ and $\phi (e)\in {{{\mathcal {C}}}}$ for all edges *e* not incident to *v*. This implies that if we remove $e_2$ and the loop incident to *v* from *G*, the resulting graph is cyclic. In other words, it suffices to consider the case when $e_1$ or $e_2$ is a loop.

We hence assume that $e_1$ is the loop incident to *v*. Let $e_3$ be the remaining non-loop edge incident to *v*, where $\phi (e_3)\in {{{\mathcal {C}}}}$. Observe that the gain of the non-loop edge $e_2$ is not included in $\bar{{{\mathcal {C}}}}$, since otherwise $G-e_1$ becomes cyclic, contradicting the assumption that *G* is essential. Therefore, $\phi (e_2\cdot e_3^{-1})\notin \bar{{{\mathcal {C}}}}$, and the loop-2-reduction at *v* adds the edge $e_2\cdot e_3^{-1}$ to the cyclic (2, 3)-g-sparse graph $G-v$. By Lemma 7.7, the resulting gain graph is ${{{\mathcal {D}}}}$-sparse. $\square$

By using Lemma 9.8, we can now prove an important consequence for graphs that are not essentially 4-edge-connected.

#### **Lemma 9.9**

Let $G=(V,E)$ be a 2-connected essential ${{{\mathcal {D}}}}$-gain graph with $|V|=n\ge 4$. Suppose that *G* is not essentially 4-edge-connected. Then, *G* has a vertex at which a 2-reduction or a loop-2-reduction is admissible.

#### *Proof*

Since *G* is 2-edge-connected and is not essentially 4-edge-connected, there exists a subset *X* of *V* for which $|X|>1$, $|V{\setminus } X|>1$ and $d_G(X)=2$. Since *G* is not $C_n^{\circ }$, we can suppose that *B*(*X*) contains a vertex *v* not incident to a loop, where *B*(*X*) denotes a set of vertices of *X* adjacent to some vertices of $V{\setminus } X$. By the 2-connectivity, *v* is not a cut-vertex. Hence, denoting the four edges incident to *v* by $e_1,\ldots ,e_4$, we may assume that $e_1,e_2,e_3$ are included in the subgraph induced by *X* while $e_4$ is not.

Note that *v* is a vertex of degree 3 in $G-e_4$, and hence, by Lemma 7.5, a 1-reduction at *v* is admissible in $G-e_4$. Without loss of generality, we may assume that $G-v+e_1\cdot e_2^{-1}$ (obtained by a 1-reduction at *v* in $G-e_4$) is ${{{\mathcal {D}}}}$-sparse.

We now consider adding $e_3\cdot e_4^{-1}$ to $G-v+e_1\cdot e_2^{-1}$ to complete the 2-reduction at *v*. Let $G'=G-v+e_1\cdot e_2^{-1}+e_3\cdot e_4^{-1}$, and suppose that $G'$ does not satisfy (C1). Since any balanced tight set *F* is 2-edge-connected if $|F|>1$, there is no balanced tight set *F* for which *V*(*F*) contains both endvertices of $e_3\cdot e_{4}^{-1}$ unless $|F|=1$. If $G-v+e_1\cdot e_2^{-1}$ has a balanced set *F* such that $|F|=1$ and *V*(*F*) contains both endvertices of $e_3\cdot e_{4}^{-1}$, then the edge in *F*, denoted by *f*, is incident to $e_3$ and $e_4$ and connects between *X* and $V{\setminus } X$. However, since $d_G(X)=2$, $|X|>1$ and $|V{\setminus } X|>1$, the vertex incident to $e_4$ and *f* turns out to be a cut-vertex of *G*, contradicting the 2-connectivity of *G*. Thus, $G'$ satisfies (C1).

If $G'$ does not satisfy (C2), it is cyclic. By Lemma 9.3, *G* is almost near-cyclic, and we can apply Lemma 9.8 to conclude that a 2-reduction or a loop-2-reduction is admissible at some vertex *v*. $\square$

The final special case is when *G* has a vertex *v* with $|N(v)|=2$.

#### **Lemma 9.10**

Let $G=(V,E)$ be a 2-connected essential ${{{\mathcal {D}}}}$-gain graph. Suppose that *G* has a vertex *v* with $|N(v)|=2$ that is not incident to a loop. Then, there is a vertex at which a 2-reduction is admissible.

#### *Proof*

If *v* is special, Lemma 9.7 implies the claim.

If *v* is not special, then there are three parallel edges between *v* and a neighbor of *v*. By the 4-regularity, if $|V|\ge 4$, *G* is not essentially-4-edge-connected, and thus Lemma 9.9 implies the statement.

If $|V|=3$, *G* is equal to the graph (shown in Fig. 15) of three vertices $V=\{u,v,w\}$, three parallel edges $e_1,e_2,e_3$ between *u* and *v*, a loop *l* attached to *w*, and two remaining edges *uw* and *vw*, denoted by $f_1$ and $f_2$, respectively. We may assume $\phi (f_1)=\phi (f_2)=\mathrm{id}$. Let ${{{\mathcal {C}}}}$ be the subgroup generated by $\phi (l)$. Since *G* is not cyclic, there is an unbalanced cycle whose gain is not included in $\bar{{{\mathcal {C}}}}$.

If a triangle, say $e_1f_1f_2$ has a gain not included in $\bar{{{\mathcal {C}}}}$, then the 2-reduction at *u* through $(e_1,f_1)$ and $(e_2,e_3)$ results in a ${{{\mathcal {D}}}}$-sparse $P_2^{2}$. Otherwise, removing $e_2$ and $e_3$ results in a cyclic graph. Then *G* is almost near-cyclic, and Lemma 9.8 implies the statement. $\square$

**Fig. 14:**
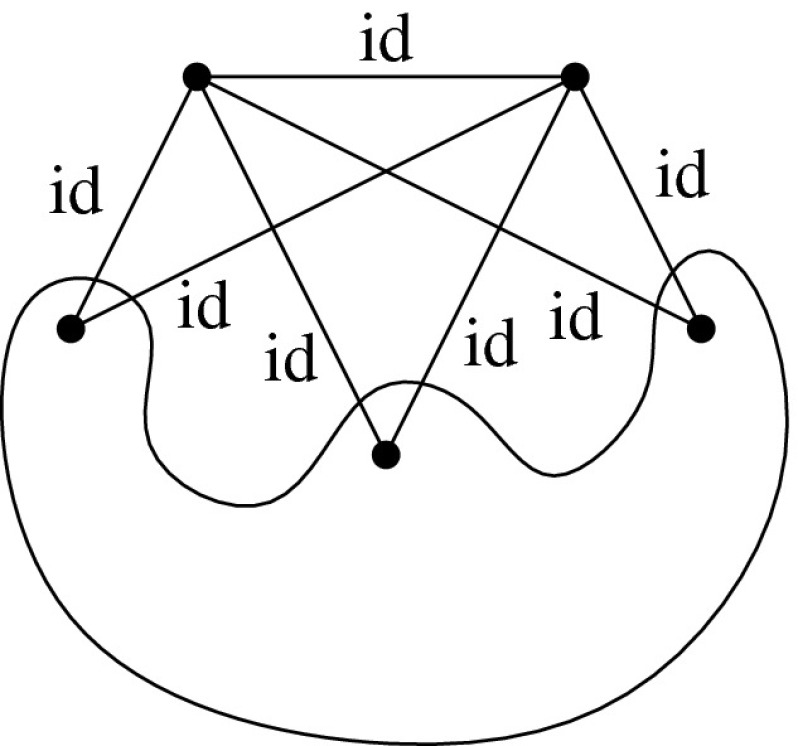
A hat subgraph

### The Remaining Cases

In a graph *G*, the *star* of a vertex *v* means the subgraph of *G* whose vertex set is $N(v)\cup \{v\}$ and the edge set is the set of edges incident to *v*. A *hat subgraph* is a balanced subgraph whose underlying graph is a hat. See Fig. 14 for an example. The following claim, together with the previous lemmas, will complete the proof of Theorem 9.1.

#### **Theorem 9.11**

Let $G=(V,E)$ be a 2-connected, essentially 2-edge-connected, and essential graph with $|V|\ge 3$. Suppose also that *G* is not almost near-cyclic. Then, for every vertex $v\in V$ that is not incident to a loop with $|N(v)|\ge 3$, either a 2-reduction at *v* is admissible or the star of *v* is contained in a hat subgraph.

In Sect. 9.3.1, we focus on the case of $|N(v)|=4$. Lemma 9.12 says that if the 2-reduction is not admissible then *G* has an *obstacle* around *v*. We will investigate intersection properties of obstacles. The corresponding results for the case of $|N(v)|=3$ will be given in Sect. 9.3.2. In Sect. 9.3.3, we prove Theorem 9.11 based on the intersection properties of obstacles.

In the rest of this section, $\mathrm{cl}_{{{\mathcal {D}}}}$ denotes the closure operator of the underlying matroid ${{{\mathcal {M}}}}_{{{\mathcal {D}}}}(G,\phi )$.

**Fig. 15:**
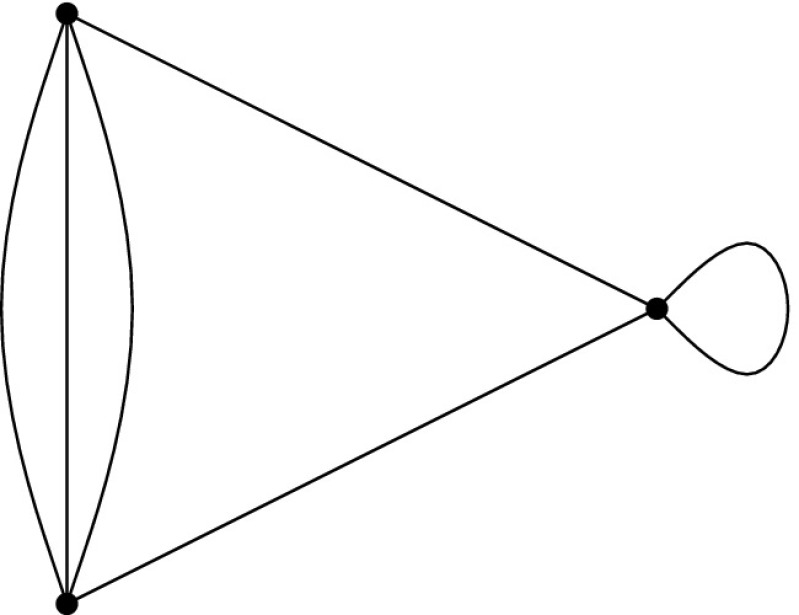
The special graph given in the proof of Lemma 9.10

#### Obstacles Around a Vertex *v* with $|N(v)|=4$

Throughout Sect. 9.3.1, $(G,\phi )$ denotes a ${{{\mathcal {D}}}}$-gain graph satisfying the assumptions of Theorem 9.11, *v* denotes a vertex with $|N(v)|=4$, $N(v)=\{a,b,c,d\}$, and $E_v$ denotes the set of edges incident to *v*.

An edge subset *F* is called *sub-tight* if $|F|=2|V(F)|-4$ and *F* is balanced. We first make a simple observation which describes the situation where 2-reductions are not admissible.

##### **Lemma 9.12**

Suppose that the 2-reduction through (*av*, *vb*) and (*cv*, *vd*) is not admissible. Then there is an edge subset $F\subseteq E{\setminus } E_v$ satisfying one of the following properties:

(i) *F* is balanced tight with $a,b\in V(F)$ and $av\cdot vb\in \mathrm {cl}_{{{\mathcal {D}}}}(F)$;
(ii) *F* is balanced tight with $c,d\in V(F)$ and $cv\cdot vd\in \mathrm {cl}_{{{\mathcal {D}}}}(F)$;
(iii) *F* is sub-tight with $a,b,c,d\in V(F)$, $F+av\cdot vb$ is balanced tight, and $cv\cdot vd\in \mathrm {cl}_{{{\mathcal {D}}}}(F+av\cdot vb)$.

##### *Proof*

Let us first consider the graph $G'=G-v+av\cdot vb$. If $G'$ is not ${{{\mathcal {D}}}}$-sparse, then, by Lemma 9.2, $E{\setminus } E_v$ has a balanced tight set *F* with $a,b\in V(F)$ and $av\cdot vb\in \mathrm {cl}_{{{\mathcal {D}}}}(F)$, which satisfies property (i).

Hence, let us assume that $G'$ is ${{{\mathcal {D}}}}$-sparse. If $G'+cv\cdot vd$ is cyclic, Lemma 9.3 implies that *G* is almost near-cyclic, contradicting the assumption that *G* is not almost near-cyclic. Therefore, $G'+cv\cdot vd$ satisfies (C2). By Lemma 9.2, there exists a balanced tight set $F'\subseteq E{\setminus } E_v\cup \{av\cdot vb\}$ with $c,d\in V(F')$ and $cv\cdot vd\in \mathrm {cl}_{{{\mathcal {D}}}}(F')$. Depending on whether $av\cdot vb\in F'$ or not, we find a desired subset of the statement; if $av\cdot vb\not \in F'$ then $F'$ is the one satisfying property (ii); otherwise $F'-av\cdot vb$ satisfies property (iii) (we remark that, in the latter case, $V(F'-av\cdot vb)$ contains *a*, *b*, *c*, *d* since $F'$ is 2-edge-connected). $\square$

Since the first and the second cases of the statement of Lemma 9.12 are symmetric, we basically have two types of *obstacles*: for a vertex *v* and $N(v)=\{a,b,c,d\}$, $F\subseteq E{\setminus } E_v$ is called an *obstacle of type* 1 (for the 2-reduction through (*av*, *vb*) and (*cv*, *vd*)) if *F* satisfies (i) or (ii) of Lemma 9.12; *F* is called an *obstacle of type* 2 if *F* satisfies (iii).

As noted above, we have three possible ways for a 2-reduction at *v*, through (*av*, *vb*) and (*cv*, *vd*), through (*av*, *vc*) and (*bv*, *vd*), and through (*av*, *vd*) and (*bv*, *vc*). By Lemma 9.12, if none of them are admissible, $E{\setminus } E_v$ contains three corresponding obstacles *X*, *Y*, *Z*. We now investigate properties of these obstacles.

We begin with a property of type 2 obstacles.

##### **Lemma 9.13**

Suppose that *X* is an obstacle of type 2 for the 2-reduction through (*av*, *vb*) and (*cv*, *vd*). Then, the following holds for *X*:

- $$|X\cup E_v|=2|V(X\cup E_v)|-2$$;
- There is an equivalent gain function $\phi '$ to $\phi$ such that $\phi '(e)=\mathrm{id}$ for $e\in X\cup \{va,vb\}$, and $\phi '(vc)=\phi '(vd)\ne \mathrm{id}$;
- $$X\cup E_v$$ is cyclic.

##### *Proof*

By definition, $|X|=2|V(X)|-4$, and hence $|X\cup E_v|=2|V(X\cup E_v)|-2$ by $N(v)\subseteq V(X)$.

Since $cv\cdot vd\in \mathrm{cl}_{{{\mathcal {D}}}}(X+av\cdot vb)$ and $X+av\cdot vb$ is balanced, $X+av\cdot vb+cv\cdot vd$ is also balanced. Hence, by Lemma 2.3, there is an equivalent gain function $\phi '$ to $\phi$ such that $\phi '(e)=\mathrm{id}$ for $e\in X$ and $\phi '(av\cdot vb)=\phi '(cv\cdot vd)=\mathrm{id}$. We thus have $\phi '(av)=\phi '(bv)=g$ and $\phi '(cv)=\phi '(dv)=g'$ for some $g,g'\in {{{\mathcal {D}}}}$. By performing a switching operation at *v* with *g* if necessary, we may assume that $\phi '(av)=\phi '(bv)=\mathrm{id}$ and $\phi '(cv)=\phi '(dv)=g'g^{-1}$. If $g'g^{-1}=\mathrm{id}$, $X\cup E_v$ becomes a balanced set with $|X\cup E_v|>2|V(X\cup E_v)|-3$, contradicting the ${{{\mathcal {D}}}}$-sparsity of *G*. Thus, $\phi '(cv)=\phi '(dv)\ne \mathrm{id}$, and $X\cup E_v$ is cyclic. $\square$

In the same manner we also have the following technical lemma.

##### **Lemma 9.14**

Let *X* and *Y* be obstacles for the 2-reduction through (*av*, *vb*) and (*cv*, *vd*) and through (*av*, *vc*) and (*bv*, *vd*), respectively. Suppose that *X* is type 2 and $X\cup Y$ is cyclic. Then, $X\cup Y\cup E_v$ is cyclic.

##### *Proof*

Since *X* is balanced and $X\cup Y$ is cyclic, for some cyclic subgroup ${{{\mathcal {C}}}}$ of ${{{\mathcal {D}}}}$, there is an equivalent gain function $\phi '$ to $\phi$ such that $\phi '(e)=\mathrm{id}$ for every $e\in X$ and $\phi '(e)\in {{{\mathcal {C}}}}$ for every $e\in Y$ by Lemma 2.3. Moreover, since $X+av\cdot vb$ and $X+av\cdot vb+cv\cdot vd$ are balanced, we have $\phi '(av\cdot vb)=\phi '(cv\cdot vd)=\mathrm{id}$. As in the previous proof, by applying a switching operation at *v*, we may assume that $\phi '(va)=\phi '(vb)=\mathrm{id}$ and $\phi '(vc)=\phi '(vd)$.

By the definition of the obstacles (whether type 1 or type 2), $$Y+$$$$Y+av\cdot vc$$ or $$Y+bv\cdot vd$$ is connected and balanced. Hence $$\phi '(av\cdot vc)\in \bar{{{\mathcal {C}}}}$$ or $$\phi '(bv\cdot vd)\in \bar{{{\mathcal {C}}}}$$, which implies $$\phi '(vc)=\phi '(vd)\in \bar{{{\mathcal {C}}}}$$. Thus, every label of $$X\cup Y\cup E_v$$ is included in $$\bar{{{\mathcal {C}}}}$$. $$\square $$

The following lemmas describe different relations among obstacles.

##### **Lemma 9.15**

Let *X* and *Y* be obstacles for the 2-reduction through (*av*, *vb*) and (*cv*, *vd*) and through (*av*, *vc*) and (*bv*, *vd*), respectively. If $X\cap Y\ne \emptyset$, then $X\cup Y$ is not a balanced set.

##### *Proof*

Suppose for a contradiction that $X\cup Y$ is a balanced set with $X\cap Y\ne \emptyset$.

(Case 1) If both *X* and *Y* are of type 1, $X\cup Y$ is tight by Lemma 7.2 and hence $|X\cup Y|=2|V(X\cup Y)|-3$. Without loss of generality, we may assume that $a,b,c\in V(X\cup Y)$, $av\cdot vb\in \mathrm {cl}_{{{\mathcal {D}}}}(X)$ and $av\cdot vc\in \mathrm {cl}_{{{\mathcal {D}}}}(Y)$. Since $X\cup Y$ is balanced, there is an equivalent gain function $\phi '$ to $\phi$ such that $\phi '(e)=\mathrm{id}$ for $e\in X\cup Y$. Moreover, since $av\cdot vb\in \mathrm {cl}_{{{\mathcal {D}}}}(X)$ and $av\cdot vc\in \mathrm {cl}_{{{\mathcal {D}}}}(Y)$, we have $\phi '(av)=\phi '(bv)=\phi '(cv)$. This implies that $X\cup Y\cup \{av,bv,cv\}$ is a balanced set. However, since $|X\cup Y\cup \{av,bv,cv\}|>2|V(X\cup Y\cup \{av,bv,cv\})|-3$, the existence of such a balanced set contradicts the ${{{\mathcal {D}}}}$-sparsity of *G*.

(Case 2) Let us consider the case when *X* is type 2. By definition of obstacles (whether type 1 or type 2), $Y+av\cdot vc$ or $Y+bv\cdot vd$ is balanced and 2-edge-connected. Without loss of generality, we assume that $Y+av\cdot vc$ is balanced and 2-edge-connected. By Lemma 9.13, there exists an equivalent gain function $\phi '$ to $\phi$ such that $\phi '(e)=\mathrm{id}$ for $e\in X\cup \{va, vb\}$ and $\phi '(vc)=\phi '(vd)\ne \mathrm{id}$. Moreover, since $X\cup Y$ is balanced, we may assume that $\phi '(e)=\mathrm{id}$ for $e\in Y$. Since $\phi '(av\cdot vc)\ne \mathrm{id}$ but $\phi '(e)=\mathrm{id}$ for $e\in Y$, $Y+av\cdot vc$ is unbalanced, a contradiction. $\square$

##### **Lemma 9.16**

Let *X* and *Y* be obstacles for the 2-reductions through (*av*, *vb*) and (*cv*, *vd*) and through (*av*, *vc*) and (*bv*, *vd*), respectively. If $|X|>1$ and $|Y|>1$, then $X\cap Y\ne \emptyset$.

##### *Proof*

Without loss of generality, we assume $a\in V(X)\cap V(Y)$. Recall that each balanced tight set is 2-connected if the size is more than one. By the 4-regularity of *G*, each vertex of *N*(*v*) has degree three in $G-v$. Hence, if *X* and *Y* are type 1 with $|X|>1$ and $|Y|>1$, then $X\cap Y$ contains an edge incident to *a*.

If *X* is type 2, then $X+av\cdot vb$ is balanced tight with $a,b,c,d \in V(X+av\cdot vb)$ by definition. Hence, if *Y* is type 1, then $X\cap Y$ contains an edge incident to *c* or *d*.

If both *X* and *Y* are type 2, then $X\cap Y$ contains an edge incident to *d*. $\square$

##### **Lemma 9.17**

Let *X*, *Y*, *Z* be obstacles for the 2-reductions through (*av*, *vb*) and (*cv*, *vd*), through (*av*, *vc*) and (*bv*, *vd*), and through (*av*, *vd*) and (*bv*, *vc*), respectively. If there is no hat subgraph containing the star of *v*, then $X\cap Y\ne \emptyset$, $Y\cap Z\ne \emptyset$ or $Z\cap X\ne \emptyset$ holds.

##### *Proof*

Note that a type 2 obstacle consists of more than one edge. If two of *X*, *Y* and *Z* are not singleton sets, then the lemma follows from Lemma 9.16. Hence we may assume that $|Y|=|Z|=1$, and denote $Y=\{e_y\}$ and $Z=\{e_z\}$. Clearly, $e_y\ne e_z$.

(Case 1) Let us first consider the case when *X* is also a singleton set. Let $X=\{e_x\}$. Depending on the relative position of $e_x, e_y$ and $e_z$, we have two situations: (I) $e_x, e_y$ and $e_z$ share a vertex or (II) $e_x, e_y$ and $e_z$ form a triangle.

In case (I), the star of *v* is included in a hat subgraph. Indeed, if denoting without loss of generality $e_x=ab$, $e_y=ac$, and $e_z=ad$, $\{e_x,e_y,e_z,va,vb,vc,vd\}$ forms a hat if it is balanced. Since *X*, *Y* and *Z* are obstacles, we have $\phi (e_x)=\phi (av \cdot vb)$, $\phi (e_y)=\phi (av \cdot vc)$ and $\phi (e_z)=\phi (av\cdot vd)$, and hence this subgraph is indeed balanced.

In case (II), without loss of generality, we assume $e_x=ab, e_y=bc$ and $e_z=ca$. Then $\{e_x,e_y,e_z,va,vb,vc\}$ forms $K_4$. Since $\phi (e_x)=\phi (av \cdot vb)$, $\phi (e_y)=\phi (bv \cdot vc)$ and $\phi (e_z)=\phi (cv \cdot va)$, this $K_4$ does not have any unbalanced cycle. Therefore, Case (II) cannot happen because of the ${{{\mathcal {D}}}}$-sparsity of *G*, as a balanced $K_4$ is not ${{{\mathcal {D}}}}$-sparse.

(Case 2) Next, we consider the case when $|X|>1$. We further split the proof into two subcases depending on whether *X* is type 1 or type 2.

If *X* is type 2, then $|X\cup E_v|=2|V(X\cup E_v)|-2$ by Lemma 9.13. Also, by Lemma 9.13, there exists an equivalent gain function $\phi '$ to $\phi$ such that $\phi '(e)=\mathrm{id}$ for $e\in X\cup \{va, vb\}$ and $\phi '(vc)=\phi '(vd)\ne \mathrm{id}$. Denote $\phi '(vc)$ by *g*. Since *Y* and *Z* are obstacles, we have $\phi '(e_y)=\phi '(e_z)=g$, which in particular implies $e_y,e_z\not \in X$. By $N(v)\subseteq V(X)$ and $e_y\ne e_z$, $|X\cup Y\cup Z\cup E_v|=2|V(X\cup Y\cup Z\cup E_v)|$, which in turn implies $E=X\cup Y\cup Z\cup E_v$. Notice that the label of each edge in $X\cup Y\cup Z\cup E_v$ is either the identity or *g*. In other words, $X\cup Y\cup Z\cup E_v$ is cyclic, contradicting the ${{{\mathcal {D}}}}$-sparsity of *G*.

The remaining case is when *X* is type 1. Without loss of generality we assume $a,b\in V(X)$. By $|X|>1$ and Lemma 7.1, $d_X(a)\ge 2$ and $d_X(b)\ge 2$. Since $e_y$ is either *ac* or *bd* and $e_z$ is either *ad* or *bc*, it suffices to consider the following two cases by symmetry: (i)$(e_y, e_z)=(ac,ad)$, and (ii)$(e_y,e_z)=(ac,bc)$.

In subcase (i), $X\cap Y$ or $X\cap Z$ contains an edge incident to *a* as $d_X(a)\ge 2$ and $d_{G-v}(a)=3$.

In subcase (ii), notice that, $\{av,bv,cv, e_y,e_z, av\cdot vb\}$ is a circuit of the underlying ${{{\mathcal {D}}}}$-sparsity matroid since it forms a balanced $K_4$. By $av\cdot vb\in \mathrm {cl}_{{{\mathcal {D}}}}(X)$, we have $cv\in \mathrm {cl}_{{{\mathcal {D}}}}(X+av+bv+e_y+e_z)\subseteq \mathrm {cl}_{{{\mathcal {D}}}}(E-cv)$, contradicting the independence of *E*. Therefore, this case does not occur and the proof is complete. $\square$

#### Obstacles Around a Vertex *v* with $|N(v)|=3$

In this subsection we shall investigate *obstacles* for a 2-reduction at a vertex *v* with $|N(v)|=3$. Most of the arguments are similar to the previous subsection. Throughout Sect. 9.3.2, $(G,\phi )$ denotes a ${{{\mathcal {D}}}}$-gain graph satisfying the assumptions of Theorem 9.11, *v* denotes a vertex with $|N(v)|=3$, $N(v)=\{a,b,c\}$, and $E_v$ denotes the set of edges incident to *v*. Without loss of generality, we assume that there are parallel edges $e_1$ and $e_2$ between *v* and *a*, and we denote $E_v=\{e_1,e_2,vb,vc\}$.

We again have three possible ways for a 2-reduction at *v*. In each case, there exists an obstacle if the operation is not admissible. The proof of the following claim is identical to that of Lemma 9.12 and hence is omitted.

##### **Lemma 9.18**

Suppose that the 2-reduction through $(e_1,vb)$ and $(e_2,vc)$ is not admissible. Then there is an edge subset $F\subseteq E{\setminus } E_v$ satisfying one of the following properties:

(i) *F* is balanced tight with $a,b\in V(F)$ and $e_1\cdot vb\in \mathrm {cl}_{{{\mathcal {D}}}}(F)$;
(ii) *F* is balanced tight with $a,c\in V(F)$ and $e_2\cdot vc\in \mathrm {cl}_{{{\mathcal {D}}}}(F)$;
(iii) *F* is sub-tight with $a,b,c\in V(F)$, $F+e_1\cdot vb$ is balanced tight, and $e_2\cdot vc\in \mathrm {cl}_{{{\mathcal {D}}}}(F+e_1\cdot vb)$.

For the 2-reduction through $(e_1,e_2)$ and (*bv*, *vc*), we encounter an even simpler situation.

##### **Lemma 9.19**

Suppose that the 2-reduction through $(e_1,e_2)$ and (*bv*, *vc*) is not admissible. Then there is a balanced tight set $F\subseteq E{\setminus } E_v$ with $b,c\in V(F)$ and $bv\cdot vc\in \mathrm {cl}_{{{\mathcal {D}}}}(F)$.

##### *Proof*

Note that $e_1\cdot e_2^{-1}$ is a loop. $G-v+e_1\cdot e_2^{-1}$ is ${{{\mathcal {D}}}}$-sparse by Lemma 9.2 since adding an unbalanced loop does not affect (C1). Note that $G-v+e_1\cdot e_2^{-1}+bv\cdot vc$ is connected. If $G-v+e_1\cdot e_2^{-1}+bv\cdot vc$ does not satisfy (C2), then Lemma 9.3 implies that *G* is almost near-cyclic, which contradicts our assumption on *G*. If $G-v+e_1\cdot e_2^{-1}+bv\cdot vc$ does not satisfy (C1), then $G-v+e_1\cdot e_2^{-1}$ contains a balanced tight set *F* with $b,c\in V(F)$ and $bv\cdot vc\in \mathrm{cl}_{{{\mathcal {D}}}}(F)$. Since a balanced tight set does not contain a loop by Lemma 7.1, we have $F\subseteq E{\setminus } E_v$. $\square$

According to Lemmas 9.18 and 9.19, we can define *the type of an obstacle* as in the previous subsection. Lemma 9.19 also says that we only encounter type 1 obstacles for the 2-reduction through $(e_1,e_2)$ and (*bv*, *vc*). The next two lemmas are counterparts of Lemmas 9.14 and 9.15, respectively, with identical proofs, which are omitted.

##### **Lemma 9.20**

Let *X* and *Y* be obstacles for distinct 2-reductions at *v*. If *X* is type 2 and $X\cup Y$ is cyclic, then $X\cup Y\cup E_v$ is cyclic.

##### **Lemma 9.21**

Let *X* and *Y* be obstacles for distinct 2-reductions at *v*. Then, if $X\cap Y\ne \emptyset$, then $X\cup Y$ is balanced.

To prove the counterpart of Lemma 9.17, we need the following two additional lemmas.

##### **Lemma 9.22**

Suppose that *Z* is an obstacle of type 1 for the 2-reduction through $(e_1,e_2)$ and (*bv*, *vc*). Then, there is an equivalent gain function $\phi '$ to $\phi$ such that $\phi '(e)=\mathrm{id}$ for $e\in Z\cup \{vb,vc\}$.

##### *Proof*

$Z+bv\cdot vc$ is balanced. Hence, by Lemma 2.3, there is an equivalent gain function $\phi '$ to $\phi$ such that $\phi '(e)=\mathrm{id}$ for $e\in Z+bv\cdot vc$. By performing a switching operation at *v* with $\phi '(bv)$ if necessary, we may assume that $\phi '(bv)=\phi '(vc)=\mathrm{id}$. $\square$

##### **Lemma 9.23**

Let *X* be an obstacle of type 2 for the 2-reduction through $(e_1,vb)$ and $(e_2,vc)$. Suppose further that there is no obstacle of type 1 for the 2-reduction through $(e_1,vb)$ and $(e_2,vc)$. Then $d_X(a)+d_X(b)+d_X(c)\ge 5$ holds.

##### *Proof*

Let $X'=X+e_1\cdot vb$. By definition, $X'$ is balanced tight with $a,b,c\in V(X')$ and $|X'|>1$. Such a balanced tight set is 2-connected and essentially 3-edge-connected by Lemma 7.1. We thus have $d_{X'}(u)\ge 2$ for $u\in \{a,b,c\}$.

Suppose that $d_{X'}(a)=d_{X'}(b)=2$. Since $X'$ is essentially 3-edge-connected and $e_1\cdot vb$ is incident to *a* and *b*, $X'$ must be a triangle on *a*, *b*, *c*. This means that *X* contains an edge linking from *a* to *c*, denoted by $e'$. Recall that $X'+e_2\cdot vc$ is balanced by definition of type 2 obstacles. However, since $e'$ and $e_2\cdot vc$ are parallel, for $X'+e_2\cdot vc$ to be balanced, $\{e',e_2\cdot vc\}$ has to be a balanced 2-cycle, that is, $\{e'\}$ is a type 1 obstacle for the 2-reduction through $(e_1,vb)$ and $(e_2,vc)$, contradicting the assumption of the lemma.

Therefore, $d_{X'}(a)\ge 3$ or $d_{X'}(b)\ge 3$, implying $d_{X'}(a)+d_{X'}(b)+d_{X'}(c)\ge 7$. Since $X'=X+e_1\cdot vb$, we obtain $d_X(a)+d_X(b)+d_X(c)\ge 5$. $\square$

##### **Lemma 9.24**

Let *X*, *Y*, *Z* be obstacles for the 2-reductions through $(e_1,vb)$ and $(e_2,vc)$, through $(e_1,vc)$ and $(e_2,vb)$, and through $(e_1,e_2)$ and (*bv*, *vc*), respectively. Then, $X\cap Y\ne \emptyset$, $Y\cap Z\ne \emptyset$, or $Z\cap X\ne \emptyset$ holds.

##### *Proof*

We split the proof into two cases depending on whether a type 1 obstacle exists for the 2-reduction through $(e_1,vb)$ and $(e_2,vc)$.

(Case 1) Suppose that there is no type 1 obstacle for the 2-reduction through $(e_1,vb)$ and $(e_2,vc)$. Then, *X* is type 2. By Lemma 9.23, $d_X(a)+d_X(b)+d_X(c)\ge 5$ holds. If $d_X(a)\ge 2$, then $X\cap Y$ contains an edge incident to *a* since $d_{G-v}(a)=2$ and $d_Y(a)\ge 1$. If $d_X(a)=1$, then we have $d_X(b)\ge 2$ and $d_X(c)\ge 2$. Since $d_{G-v}(b)=d_{G-v}(c)=3$, $|Z|=1$ holds if $X\cap Z= \emptyset$. However, in this case, we have $d_{X\cup Z}(b)=d_{X\cup Z}(c)=3$, and thus $X\cap Y$ or $Y\cap Z$ contains an edge incident to *b* or *c*.

**Fig. 16:**
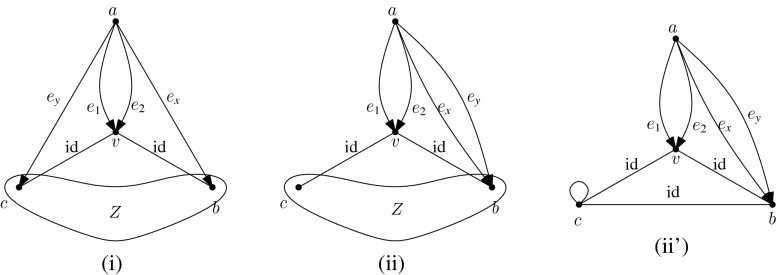
Proof of Lemma 9.24

In a symmetric manner, we are done in the case when a type 1 obstacle does not exist for the 2-reduction through $(e_1,vc)$ and $(e_2,vb)$.

(Case 2) We now consider the case when both *X* and *Y* are type 1. If $|X|>1$ or $|Y|>1$, then *X* or *Y* is 2-connected, and hence $X\cap Y$ contains an edge incident to *a* as $d_{G-v}(a)=2$. We thus assume $|X|=|Y|=1$ and $X\ne Y$. Let us denote $X=\{e_x\}$ and $Y=\{e_y\}$. Without loss of generality, we assume that $e_x$ connects from *a* to *b*. Also, by Lemma 9.22, we may assume $\phi (e)=\mathrm{id}$ for $e\in Z\cup \{vb,vc\}$. Since $e_1\cdot vb\in \mathrm {cl}_{{{\mathcal {D}}}}(X)$, we have $\phi (e_x)=\phi (e_1\cdot vb)=\phi (e_1)$. The proof is completed by a further case analysis: (i) $e_y$ connects from *a* to *c* or (ii) $e_y$ connects from *a* to *b* (see Fig. 16).

In case (i), we have $e_1\cdot vc\in \mathrm {cl}_{{{\mathcal {D}}}}(Y)$ by definition. Therefore, $\phi (e_y)=\phi (e_1\cdot vc)=\phi (e_1)$. Notice that $\{e_1,vb,vc,e_x,e_y, bv\cdot vc\}$ forms a $K_4$ without unbalanced cycles by $\phi (e_y)=\phi (e_1)=\phi (e_x)$. Moreover, since $bv\cdot vc\in \mathrm {cl}_{{{\mathcal {D}}}}(Z)$, we obtain $e_1\in \mathrm {cl}_{{{\mathcal {D}}}}(\{vb,vc,e_x,e_y,bv\cdot vc\})\subseteq \mathrm {cl}_{{{\mathcal {D}}}}(E-e_1)$. This contradicts the independence of *E* in the underlying ${{{\mathcal {D}}}}$-sparsity matroid.

Let us consider case (ii). If $|Z|>1$, then $X\cap Z$ or $Y\cap Z$ contains an edge incident to *b* as *Z* is type 1 and $d_Z(b)\ge 2$. Suppose that $|Z|=1$, $X\cap Y=\emptyset$, $X\cap Z=\emptyset$ and $Y\cap Z=\emptyset$. Then $X\cup Y\cup Z\cup E_v$ induces a subgraph in which *v*, *a* and *b* have degree four. So, if $|V|>4$, then *c* becomes a cut-vertex, contradicting the 2-connectivity of *G*. On the other hand, if $|V|=4$, then *G* becomes the graph shown in Fig. 16(ii’). In this case removing $e_2$ and $e_y$ results in a cyclic graph (where any cycle except the loop is balanced by $\phi (e_1)=\phi (e_x)$). This means that *G* is almost near-cyclic, a contradiction. $\square$

#### Proof of Theorem 9.11

##### *Proof of Theorem 9.11*

Suppose that no 2-reduction is admissible at *v*. Then we have three obstacles *X*, *Y* and *Z* for the three possible 2-reductions at *v*. Suppose further that the star of *v* is not contained in a hat subgraph. Then, by Lemmas 9.17 and 9.24, we may assume without loss of generality that $X\cap Y\ne \emptyset$ holds.

If $|X\cup Y|\ge 2|V(X\cup Y)|-1$, then $V(X\cup Y)\cup \{v\}=V$ must hold since *G* is essentially 4-edge-connected. We then have $|X\cup Y\cup E_v|\ge 2|V|+1$, contradicting the ${{{\mathcal {D}}}}$-sparsity of *G*.

Therefore we have

20 $$\begin{aligned} |X\cup Y|\le 2|V(X\cup Y)|-2. \end{aligned}$$

To derive a contradiction, we next show that the number of connected components in $(V(X)\cap V(Y),X\cap Y)$ is equal to two. To see this, let $c_0$ be the number of trivial connected components (i.e., singleton vertex components) in $(V(X)\cap V(Y), X\cap Y)$ and let $c_1$ be the number of nontrivial connected components in it. Then,

21 $$\begin{aligned} |X|+|Y|&\ge 2|V(X)|-4\,{+}\,2|V(Y)|-4=2|V(X\cup Y)|\,{+}\,2|V(X\cap Y)|\,{+}\,2c_0-8, \end{aligned}$$

22 $$\begin{aligned} |X \cap Y|&\le 2|V(X\cap Y)|-3c_1, \end{aligned}$$

where the last inequality comes from $|F|\le 2|V(F)|-3$ for any non-empty $F\subseteq X\cap Y$. From (20–22), we obtain $2c_0+3c_1\le 6$. On the other hand by $X\cap Y\ne \emptyset$ we also have $c_1\ge 1$. Hence we get $c_1+c_2\le 2$, and the number of connected components in the graph $(V(X)\cap V(Y),X\cap Y)$ is at most two.

If the number of connected components in $(V(X)\cap V(Y),X\cap Y)$ is one, then, since *X* and *Y* are connected and balanced, Lemma 2.4(1) implies that $X\cup Y$ is balanced, which contradicts Lemmas 9.15 and 9.21.

Thus the number of connected components in $(V(X)\cap V(Y),X\cap Y)$ is two. Then $2c_0+3c_1\ge 5$. Hence by (21) and (22) we have

23 $$\begin{aligned} |X\cup Y|\ge 2|V(X\cup Y)|-3. \end{aligned}$$

Also by Lemma 2.5$X\cup Y$ is cyclic. This implies that $X\cup Y$ is not tight, as $X\cup Y$ cannot be cyclic tight by (20).

If both *X* and *Y* are type 1, then $X\cup Y$ is tight by Lemma 7.2, which does not happen. Hence *X* or *Y* is type 2, and Lemmas 9.14 and 9.20 imply that $X\cup Y\cup E_v$ is also cyclic. Also by (23) and $N(v)\subseteq X\cup Y$ (as *X* or *Y* is type 2) we obtain $|X\cup Y\cup E_v|\ge 2|V(X\cup Y\cup E_v)|-1$. Thus, due to the essential 4-edge-connecitivity of *G*, $|V(X\cup Y\cup E_v)|\ge |V|-1$ must hold.

If $V(X\cup Y\cup E_v)=V$, then $|X\cup Y\cup E_v|=|E|-1$, and hence *G* is near cyclic, as $X\cup Y\cup E_v$ is cyclic. On the other hand, if $V(X\cup Y\cup E_v)=V-u$ for some $u\in V$, then *u* is incident to a loop and two non-loop edges by 4-regularity. Observe that removing this loop and one of the two non-loop edges results in a cyclic graph. This means that *G* is almost near-cyclic.

In both cases *G* turns out to be almost near-cyclic, which contradicts the assumption on *G*. This completes the proof. $\square$

### Proof of the Main Theorem

We are now ready to prove Theorem 9.1, which also completes the proof of Theorem 7.8.

#### *Proof of Theorem 9.1*

By Lemmas 9.5, 9.6, 9.8 and 9.9, we may assume that *G* is 2-connected, essentially 4-edge-connected, not special, and not almost near-cyclic. Also, by Lemma 9.10, we may assume that every vertex *v* with $|N(v)|=2$ is incident to a loop.

Since *G* is not special, *G* has a vertex *v* that is not incident to a loop. Then $|N(v)|\ge 3$. By Theorem 9.11, either the 2-reduction at *v* is admissible or the star of *v* is contained in a hat subgraph *H*. Suppose the latter holds. We denote the vertices of *H* by $a_1, a_2, b_1,b_2,b_3$, and assume that $a_1$ and $a_2$ have degree four in *H* (and hence $a_1$ or $a_2$ is *v*). Since *H* is balanced, we may assume that all labels in *H* are identity. Moreover, since *G* is not a fancy hat, we may assume that $b_1$ is not incident to a loop.

We prove that some 2-reduction at $b_1$ is admissible. Suppose that no 2-reduction is admissible at $b_1$. Then, by Theorem 9.11, the star of $b_1$ is contained in a hat subgraph $H'$. Note that $H'$ is different from *H*.

We claim that $H'$ contains a triangle on $b_1,a_i,b_j$ for some $i\in \{1,2\}$ and $j\in \{2,3\}$. To see this first suppose that $a_1a_2\notin E(H')$. Then, since each vertex has degree at least 2 in $H'$, we have $a_1b_2\in E(H')$ or $a_1b_3\in E(H')$ by $N_G(a_1)=\{a_2,b_1,b_2,b_3\}$ and $a_1a_2\not \in E(H')$. This also implies $b_1b_2\in E(H')$ or $b_1b_3\in E(H')$, respectively, as $b_1$ is incident to all the vertices of $H'$. Thus $H'$ has a triangle on $b_1,a_1,b_j$ for some $j\in \{2,3\}$.

If $a_1a_2\in E(H')$, then $H'$ contains a triangle on $b_1,a_1,a_2$. In a hat subgraph, two vertices of each triangle have degree four, which implies $N(a_i)\subseteq V(H')$ for some $i\in \{1,2\}$. Therefore, $a_ib_2\in E(H')$ and $b_1b_2\in E(H')$, and hence $b_1b_2a_i$ forms a triangle.

Consequently, without loss of generality, we may assume that $H'$ contains a triangle on $b_1, b_2, a_1$. Recall that a hat subgraph is balanced. Since $\phi (a_1b_1)=\phi (a_1b_2)=\mathrm{id}$, we obtain $\phi (b_1b_2)=\mathrm{id}$ as $H'$ contains a triangle on $a_1, b_1, b_2$. Observe then that $\{a_1,a_2,b_1,b_2\}$ induces a $K_4$ in which the label of each edge is the identity. This contradicts the ${{{\mathcal {D}}}}$-sparsity of *G*. Consequently, the 2-reduction at $b_1$ is admissible. $\square$

## Concluding Remarks

The main results of this paper (Theorems 6.3 and 8.2) give rise to efficient algorithms for testing generic symmetric rigidity with rotation symmetry or dihedral symmetry ${{{\mathcal {D}}}}_{2k}$ with odd *k*. This can be done by computing the rank of the quotient graphs in the corresponding matroids ${{{\mathcal {M}}}}(g_{2,3})$ or ${{{\mathcal {M}}}}_{{{\mathcal {D}}}}(G,\phi )$. Here we briefly describe the main algorithmic ideas and show that testing independece in these matroids can be done in polynomial time.

Let $(G,\phi )$ be a gain graph with $G=(V,E)$. First consider ${{{\mathcal {M}}}}(g_{2,3})$, in which *E* is independent if and only if (i) *G* is (2, 1)-sparse and (ii) every nonempty balanced subset $F\subseteq E$ is (2, 3)-sparse, cf. Lemma 3.1. There exist efficient algorithms for testing (*k*, *l*)-sparsity for any pair of integers *k*, *l*, see e.g. [2, 10], so checking (i) is easy. Observe that *G* satisfies (ii) if and only if every minimally non-(2, 3)-sparse graph (also called a (2, 3)-circuit or an *M*-circuit) is unbalanced. Suppose that *G* satisfies (i) and consider one of its *M*-components, i.e. a subgraph *H* of *G* induced by a connected component of the (2, 3)-sparsity matroid of *G* (see [2, 7] for more details on *M*-components). Each (2, 3)-circuit is a subgraph of some *M*-component, so we may deal with them separately. The key observation is that within *H* the complements of the (2, 3)-circuits are pairwise edge-disjoint. Since the *M*-components are pairwise edge-disjoint, this shows that the number of (2, 3)-circuits in *G* is *O*(*n*) and they can easily be enumerated. Then it remains to test whether each of these circuits is unbalanced, which can be done by switching and using Lemma 2.3 (similar arguments are given in [1]).

Next consider ${{{\mathcal {M}}}}_{{{\mathcal {D}}}}(G,\phi )$, in which *E* is independent if and only if (i) *G* is (2, 0)-sparse and (ii) every cyclic subset $F\subseteq E$ is (2, 1)-sparse, and (iii) every balanced subset $F\subseteq E$ is (2, 3)-sparse. As above, testing (2, 0)-sparsity is easy. We can again observe that *G* satisfies (ii) if and only if every minimally non-(2, 1)-sparse graph (a (2, 1)-circuit) is non-cyclic. Suppose that *G* satisfies (i). Then it is easy to see that these circuits are edge-disjoint, which shows that we have *O*(*n*) circuits to check. As above, they can easily be enumerated, and we can use switching and Lemma 2.3 to see whether they are all non-cyclic. So suppose *G* satisfies (ii) as well. As above, it remains to check whether every (2, 3)-circuit is unbalanced. Let *H* be an *M*-component of *G*. It is not hard to see that $H-e$ is (2, 1)-sparse for all $e\in E(H)$. Thus, by using the arguments above, it follows that we have $O(n^2)$ circuits to enumerate and test, which can also be done efficiently by the same techniques.
